# Utilisation and Shortages of Dental Medicines in Australia From 2014 to 2025: A Retrospective Time‐Series Study

**DOI:** 10.1155/ijod/7203795

**Published:** 2026-10-08

**Authors:** Jack Janetzki, Joon Soo Park, Amy Page, Debra Rowett, Wern Chern Chai, Lisa Kalisch Ellett

**Affiliations:** ^1^ School of Pharmacy and Biomedical Sciences College of Health Adelaide University City Campus West, North Terrace Adelaide Australia adelaide.edu.au; ^2^ Quality Use of Medicines and Pharmacy Research Centre College of Health Adelaide University City Campus West, North Terrace Adelaide Australia adelaide.edu.au; ^3^ Centre for Optimisation of Medicines The University of Western Australia Crawley Western Australia Australia uwa.edu.au; ^4^ International Research Collaborative—Health and Equity The University of Western Australia Crawley 6009 Western Australia Australia uwa.edu.au; ^5^ Health and Biomedical Innovation College of Health Adelaide University Adelaide Australia adelaide.edu.au

**Keywords:** analgesia, antibiotic, dental prescribing, dispensing, medicine shortage, utilisation

## Abstract

**Background:**

Dental prescribing in Australia under the Pharmaceutical Benefits Scheme (PBS) has evolved alongside antimicrobial stewardship initiatives, opioid regulation, and medicine shortages. Understanding national utilisation trends is important to inform policy and safe prescribing practices.

**Methods:**

A retrospective analysis of publicly available PBS and Repatriation PBS (RPBS) Section 85 and Section 100 Date of Supply data was conducted from February 2014 to August 2025. Dental schedule item codes were identified via the PBS Publications Archive. Monthly dispensing rates per 100,000 population were calculated using Australian Bureau of Statistics population estimates. Medicines were classified by Anatomical Therapeutic Chemical (ATC) classification third level code, with trends assessed by therapeutic class and formulation. Therapeutic Goods Administration (TGA) shortage reports were used to contextualise utilisation changes.

**Results:**

Fifty‐nine dental medicines were included in the PBS dental schedule during the study period, with 50 remaining in August 2025. Antibiotics were commonly utilised, with amoxicillin 500 mg capsules consistently most dispensed. Paracetamol with codeine remained the second most dispensed medicine despite regulatory changes. Amoxicillin with clavulanic acid use increased, while clindamycin declined except during metronidazole shortages. Oxycodone dispensing rose markedly from 2023, whereas ibuprofen use declined.

**Conclusions:**

Dental medicine utilisation shifted over time, likely reflecting stewardship efforts and supply disruptions. Ongoing review of the PBS Dental Schedule is warranted to ensure alignment with contemporary clinical guidance and optimise safe prescribing.

**Highlights**

• Amoxicillin was consistently the most dispensed dental medicine nationally.
• Antibiotics accounted for most dental Pharmaceutical Benefits Scheme (PBS) dispensings across the study period.
• Dispensing of amoxicillin with clavulanic acid increased steadily over time.
• Medicine shortages caused short‐term shifts, including higher clindamycin use.
• Oxycodone dispensing rose notably in recent years due to stewardship focus.

## 1. Introduction

Medicines prescribed in dental practice play a critical role in the prevention and management of oral disease, pain, and infection [1]. Commonly used dental medicines include antibiotics, analgesics, and topical agents [2, 3].

Dentists account for a substantial proportion of community antibiotic prescribing, making dental practice an important target for antimicrobial stewardship initiatives. Contemporary recommendations increasingly emphasise narrow‐spectrum antibiotics and nonopioid analgesia, reflecting concerns regarding antimicrobial resistance and opioid‐related harms. International studies have demonstrated considerable variation in dental prescribing patterns across countries, particularly regarding antibiotic selection and opioid use [4–7]. Despite differences between healthcare systems, several common themes have emerged, including efforts to improve antimicrobial stewardship and optimise analgesic prescribing. However, prescribing behaviour may also be influenced by external factors such as regulatory changes, evolving clinical guidelines, and disruptions to medicine supply.

Medicine shortages have become increasingly common globally and may disrupt established prescribing practices by necessitating therapeutic substitution [8, 9]. Although the impact of shortages on prescribing behaviour has been investigated in other settings, little is known about how supply disruptions influence medicines used in dentistry, where first‐line therapies are relatively limited and stewardship principles are particularly important.

Monitoring the utilisation of these medicines is important for understanding dental prescribing practices, assessing adherence to clinical guidelines, and supporting antimicrobial stewardship efforts within dentistry. A previous Australian analysis of dentist‐prescribed medicines from 2006 to 2018 demonstrated increasing use of antibiotics, opioids, and benzodiazepines, with antibiotics comprising the majority of prescriptions [6]. Since that analysis, several developments have occurred, including updates to Therapeutic Guidelines, growing antimicrobial stewardship initiatives, changes in opioid prescribing recommendations, the COVID‐19 pandemic, and recurrent medicine shortages. The influence of these events on dental medicine utilisation remains unknown.

Therefore, this study aimed to characterise temporal trends in dentist‐prescribed medicines in Australia from 2014 to 2025 and examine how changes in clinical guidance, health‐system disruptions, and medicine shortages may have influenced dispensing patterns.

## 2. Material and Methods

In Australia, many medicines to treat dental conditions are subsidised under the Pharmaceutical Benefits Scheme (PBS), the national medicine formulary [10]. PBS‐subsidised medicines are available to Australian residents holding a current Medicare card, eligible overseas visitors covered by reciprocal healthcare arrangements, and eligible veterans through the Repatriation PBS (RPBS).

Medicines subsidised by the PBS are given a PBS item code [10]. This is a unique identifier used to reimburse pharmacies for dispensed PBS medicines. The PBS item code relates to a specific active ingredient of a medicine, its strength, dosage, formulation and pack size, and listing type (general, restricted, or authority). Different brands of medicine may have the same or different PBS item codes. Dental items subsidised on the PBS each have a unique PBS item code that is separate to that which is used by medical practitioners, nurse practitioners, midwives, or optometrists and are available on a specific dental schedule [11]. Therefore, to identify current and historical PBS item codes corresponding to dental medicines, we searched the PBS Publications Archive [12]. For each month within the study period (between February 2014 and August 2025), the PBS text files were reviewed, with specific attention to the ‘Prescriber type’ and ‘Prescriber type multi’ reports contained within these files [12]. In the monthly PBS Publications Archive, dental items are identifiable by the presence of a ‘D’ designation, indicating dental, meaning that these medicines can be prescribed exclusively by dentists.

PBS item codes related to dental items were extracted for each month across the study period from the PBS and RPBS Section 85 and Section 100 Date of Supply data [13].

Duplicate PBS item codes were then removed to define a list of unique PBS item codes representing medicines that were available to be dispensed via the PBS Schedule Dental Items list at any point in the study period (Appendix Table A).

### 2.1. Extraction of Utilisation Data

For each unique dental PBS item code identified, corresponding utilisation data were extracted from the PBS and RPBS Section 85 and Section 100 Date of Supply Data reports [13].

### 2.2. Classification of Dental Medicines

Dental medicines were grouped into clinically relevant therapeutic classes by grouping by third level Anatomical Therapeutic Chemical (ATC) classification code.

### 2.3. Data Analysis

Descriptive analyses were conducted to examine trends in dental medicine utilisation over the study period by summarising monthly dispensing counts and expressing them as population rates per 100,000 population using estimated residential population data from the Australian Bureau of Statistics [14]. Annual and monthly utilisation patterns were assessed to identify long‐term trends and short‐term fluctuations. Changes in utilisation were examined over time by medicine class and by medicine at the formulation level.

Medicine shortages and the COVID‐19 pandemic were considered when interpreting observed trends. The Therapeutic Goods Administration (TGA) Medicine shortage reports database was used to provide additional information about which medicines were in shortage and the shortage periods [15]. When shortages for dental medicines were reported, we checked the TGA website to identify shortage mitigation strategies including availability of section 19A overseas imports or Serious Scarcity Substitution Instruments (SSSI), which allow pharmacists to dispense a different strength or formulation of the prescribed medicine without requiring a new prescription for the substitute product [16, 17]. A summary of the shortages throughout the study period affecting each of the top 10 most commonly dispensed medicines is listed in Appendix B.

### 2.4. Ethics Considerations

This study used aggregated, publicly available PBS data and did not involve individual‐level or identifiable patient information. As such, ethics approval was not required.

This study was conducted in accordance with the REporting of studies Conducted using Observational Routinely collected health Data for PharmacoEpidemiology (RECORD‐PE) guidelines.

## 3. Results

Across the study period, 59 unique medicines were available on PBS Dental Schedule. At the end of the study period, in August 2025, 50 unique medicines were available.

Results for the top 10 most commonly dispensed therapeutic groups, which accounted for more than 99.8% of total prescriptions dispensed over the study period, are presented in Figure 1. Data for all dental medicines over the study period are available in the Appendices (A and C).

Figure 1Top 10 most commonly dispensed PBS Dental Schedule classes of medicine by ATC 3rd level per 100,000 population per month between February 2014 and August 2025. (A) J01C beta‐lactam antibacterials, penicillins. (B) N02A opioids. (C) J01X other antibacterials. (D) J01F macrolides, lincosamides, and streptogramins. (E) J01D other beta‐lactam antibacterials. (F) M01A anti‐inflammatory and antirheumatic products, nonsteroids. (G) N05B anxiolytics. (H) A01A stomatological preparations. (I) N05C hypnotics and sedatives. (J) J01A tetracyclines.

**Figure:**
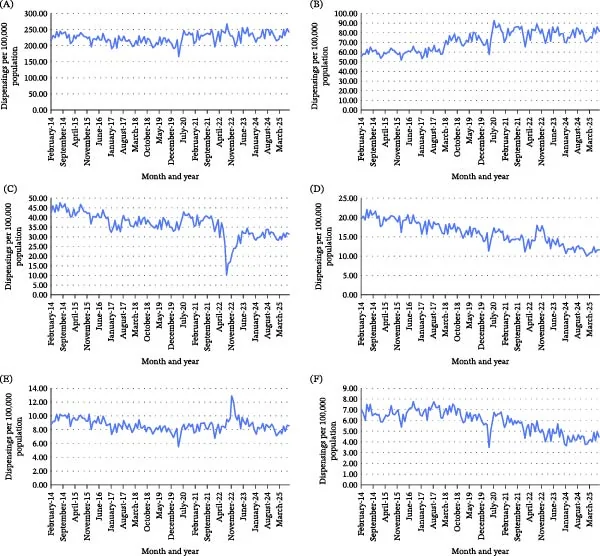

**Figure:**
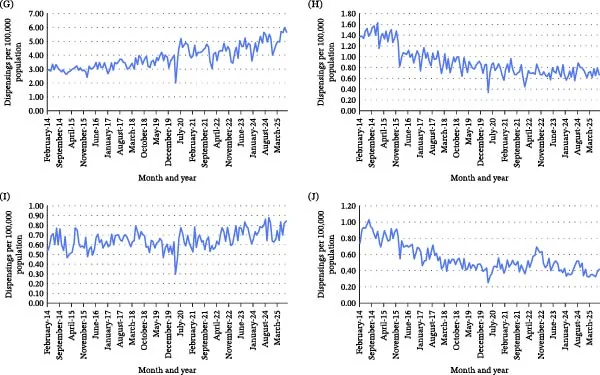

The leading therapeutic classification of dental items dispensed per 100,000 per month across the study period was beta‐lactam antibacterials and penicillins (ATC group J01C), followed by opioids (N02A); other antibacterials (J01X); macrolides, lincosamides, and streptogramins (J01F); other beta‐lactam antibacterials (J01D); nonsteroidal anti‐inflammatory and antirheumatic products (M01A); anxiolytics (N05B); stomatological preparations (A01A); hypnotics and sedatives (N05C); and lastly tetracyclines (J01A) (Figure 1).

Six of the top 10 most commonly dispensed PBS Dental Schedule medicines were antibiotics, three were analgesics, and one was an anxiolytic (Figure 2).

Figure 2Top 10 most commonly dispensed PBS Dental Schedule medicines (by total volume across study period) dispensed at the medicine level per 100,000 population per month between February 2014 and August 2025. (A) Amoxicillin. (B) Paracetamol with codeine. (C) Amoxicillin with clavulanic acid. (D) Metronidazole. (E) Clindamycin. (F) Cefalexin. (G) Ibuprofen. (H) Diazepam. (I) Oxycodone. (J) Phenoxymethylpenicillin.

**Figure:**
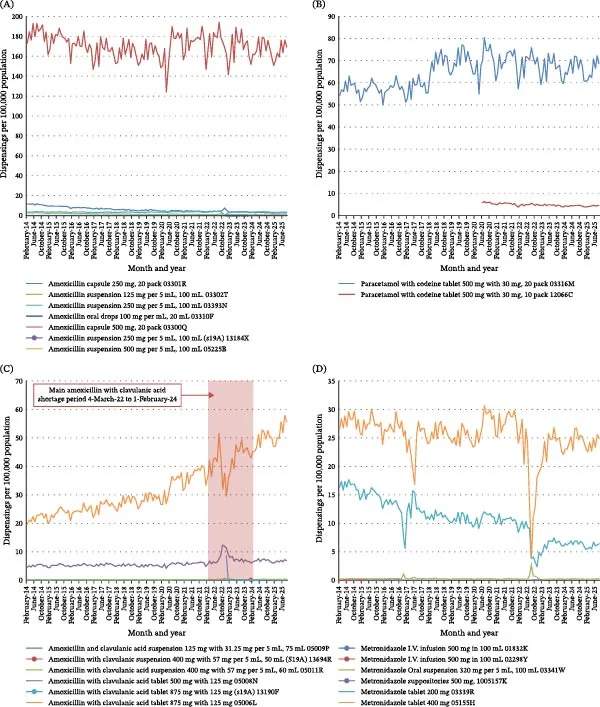

**Figure:**
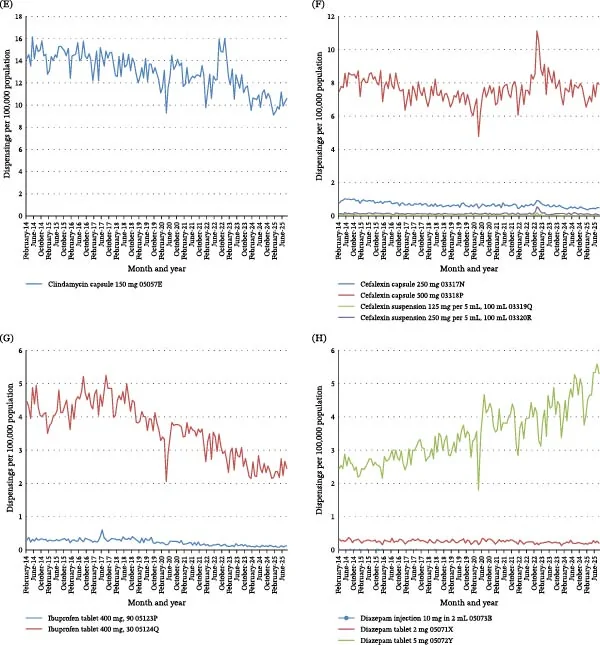

**Figure:**
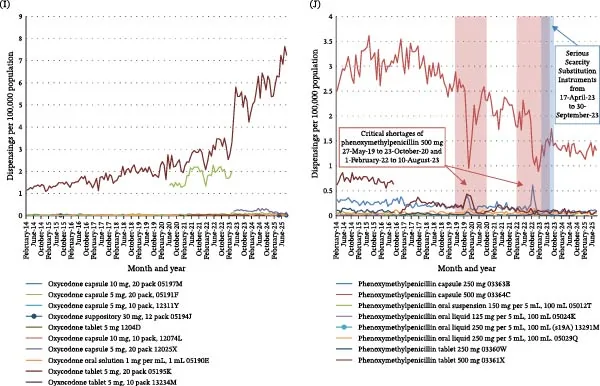

Amoxicillin was the most commonly dispensed medicine per 100,000 population per month, with 500 mg capsules accounting for, on average, 180 dispensings per 100,000 population per month across the study period (Figure 2A).

Metronidazole was the fourth most commonly dispensed medicine despite shortages in late 2016 to early 2017 and again in mid‐2022 to early 2023 (Figure 2D). Dispensings of clindamycin 150 mg capsules trended downwards throughout the study period except a brief increase in mid‐to‐late 2022 coinciding with the metronidazole shortage (Figure 2D,E).

Cefalexin dispensings were predominantly attributable to 500 mg capsules, which had an average of ~8.4 dispensings per 100,000 population each month across the study period except for a brief increase at the end of 2022 and start of 2023 (Figure 2F). This coincided with implementation of an SSSI due to several ongoing cefalexin shortages (Figure 2F, Appendix B).

Dispensings of diazepam 5 mg tablets, which accounted for most of the dispensing of diazepam, rose throughout the study period except for a sharp reduction during the early months of 2020 with subsequent rebound thereafter (Figure 2H). These dispensing changes coincided with the onset of the COVID‐19 pandemic and subsequent shortage of a single brand of diazepam from 28^th^ of April 2020 to 16^th^ of April 2021. Average dispensings of diazepam 5 mg tablets increased from 2.8 per 100,000 per month in February 2014 to more than 5 per 100,000 per month in August 2025.

Oxycodone dispensings increased throughout the study period with a marked increase in dispensings per 100,000 per month in April and May 2023 of the 5 mg strength, 20 tablet item code (Figure 2I). The use of other item codes remained comparatively low.

Phenoxymethylpenicillin dispensings were largely accounted for by the 500 mg capsule strength, 50 capsule pack, yet dispensings largely declined throughout the study period with critical shortages occurring repeatedly throughout the study period, leading to the implementation of an SSSI from April 2023 to September 2023 (Figure 2J and Appendix B).

Figures of dispensings per 100,000 population per month for each medicine other than the top 10 most commonly dispensed are provided in Appendix C.

## 4. Discussion

This study provides a contemporary national overview of utilisation patterns for PBS‐subsidised dental medicines in Australia over an 11.5‐year period. Our results demonstrate that dispensing trends have also been influenced by external factors like the COVID‐19 pandemic and recurring medicine shortages. While antibiotics remained the most commonly dispensed therapeutic class throughout the study period, utilisation patterns within and between medicine classes changed substantially over time, particularly during periods of constrained supply. Our findings are broadly consistent with those of an earlier national analysis of PBS dental prescribing from 2006 to 2018 [6]. As in the previous study, antibiotics accounted for the majority of dental PBS prescribing, with amoxicillin remaining the most frequently dispensed medicine.

A key finding of our study was the influence of medicine shortages on dispensing patterns across several commonly used dental medicines. Shortages of amoxicillin, amoxicillin with clavulanic acid, metronidazole, cefalexin, and phenoxymethylpenicillin were each associated with short‐term changes in dispensing volumes of either the affected medicine or therapeutic alternatives. For example, the national shortage of amoxicillin 500 mg capsules in late 2022 and early 2023 coincided with increased dispensing of alternative strengths and formulations of amoxicillin, supported by the implementation of an SSSI. The SSSI specifically allowed pharmacists to substitute amoxicillin, including the 500 mg capsules to an alternative strength of the same formulation (for example, two 250 mg capsules or half of a 1 g tablet) without contacting the prescriber [17]. A brief increase in 250 mg amoxicillin dispensings was observed in November 2022 just prior to the SSSI implementation, indicating that, due to the shortage, pharmacists may have already been dispensing 250 mg capsules instead with approval from prescribers. At the time of the amoxicillin shortage and SSSI implementation, there was a reduction in the monthly dispensing of the 250 mg/5 mL suspension product, which through the SSSI, could be substituted with an equivalent suspension product of a different strength.

Shortages of metronidazole aligned with a temporary increase in clindamycin use, suggesting substitution toward alternative anaerobic antimicrobial therapy when first‐line options were unavailable. In contrast to the reported increase in clindamycin use over time in the earlier study [6], our results demonstrated an overall downward trend in clindamycin dispensing over more recent years, with only a brief rise in 2022 corresponding to the metronidazole shortage (Figure 2). Although both drugs are effective in the treatment of anaerobic bacteria, the primary cause of dental abscess, clindamycin is known to cause serious adverse effects such as *Clostridium difficile* infection [18–22]. For this reason, clindamycin should only be considered in patients with hypersensitivity reactions to penicillin‐based regimens. It was therefore promising to observe a return to usual dispensing patterns of clindamycin following the metronidazole shortage period, which aligns with recommendations in the Therapeutic Guidelines antimicrobial stewardship protocol [23].

The observed increases in broader‐spectrum antibiotic use during shortage periods are particularly important from an antimicrobial stewardship perspective. Dispensing of amoxicillin with clavulanic acid increased steadily over the study period and showed more pronounced rises during periods when amoxicillin shortages were occurring. In the lead up to the amoxicillin SSSI, there was a substantial increase in amoxicillin 875 mg with clavulanic acid 125 mg dispensings with 51.5 dispensings per 100,000 population per month in August 2022. Similarly, during the amoxicillin 500 mg shortage, there was a brief increase in amoxicillin 500 mg with clavulanic acid 125 mg tablet dispensing. This indicates that prescribing and dispensing may have shifted from amoxicillin to amoxicillin with clavulanic acid during the amoxicillin shortage period. Amoxicillin with clavulanic acid then also experienced its own shortages, leading to the introduction of the availability of an overseas‐registered s19A product to the market [16]. While some of the increase in amoxicillin with clavulanic acid dispensing may reflect longer‐term changes in prescribing behaviour, the timing suggests that shortages likely contributed to substitution toward broader‐spectrum agents. This has important stewardship implications, as broader‐spectrum antibiotic exposure may contribute to antimicrobial resistance and may not always align with contemporary guideline recommendations in dentistry [23].

S19A products are those that have received regulatory approval from jurisdictions with comparably similar medicine regulation [16, 24]. Several s19A products were made available during the shortage periods including those for amoxicillin, amoxicillin with clavulanic acid, and phenoxymethylpenicillin. For both amoxicillin and phenoxymethylpenicillin, the s19A products were not greatly utilised. For amoxicillin 875 mg with clavulanic acid 125 mg tablets, the S19A product with item code 13190F was greatly utilised during the shortage period initially before supply of Australian Register of Therapeutic Goods stock was subsequently available.

Our prior analysis of overseas‐registered s19A products during shortages has demonstrated that when implemented, s19A products usually contribute to a substantial portion of medicine utilisation [24]. This is, however, dependent on several market factors including whether other brand alternatives are available, alternative strengths or formulations of the product available for use, and whether there are delays in PBS approval of the s19A product in relation to commencement of the shortage.

Beyond shortages, longer‐term utilisation trends also suggest changing prescribing practices in response to guideline updates and evolving pain management strategies. Amoxicillin remained consistently the most frequently dispensed medicine nationally, reinforcing its central role in dental infection management, which is reflected in current treatment guidelines. However, the persistence of high dispensing volumes for paracetamol with codeine, despite evolving regulatory restrictions and increasing emphasis on opioid stewardship [23], suggests that legacy prescribing patterns may remain entrenched.

The prior study reported increases in oxycodone and tramadol dispensing up to 2018 [6]. These findings were broadly consistent with our findings, which showed that opioid dispensings increased across much of the study period until November 2019, before declining during the onset of the COVID‐19 pandemic and plateauing from October 2020. Most opioid use in our study was for paracetamol with codeine 30 mg. Although oxycodone accounted for a low proportion of opioid dispensing in our study, use increased throughout the study period, with the rate of dispensings particularly increasing post‐2020. In 2019 and 2020, the Therapeutic Guidelines, Oral and Dental was updated to recommend that where opioids are required, dentists should consider prescribing oxycodone at the lowest effective dose for the shortest possible duration [4, 23]. The rise in oxycodone dispensing from 2023 onwards may reflect increasing alignment with updated Therapeutic Guidelines recommendations favouring short‐course oxycodone over codeine‐containing products where opioid analgesia is required. Despite this, the product with the greatest number of dispensings per month across the study period was oxycodone 5 mg tablet 20 pack, PBS item code 5195 K. There was considerably lower use of the PBS item code of the same product with a pack size of 10 tablets. Caution should also be taken when interpreting this data as it is unknown whether people were supplied a quantity less than 20 tablets. Our findings are, however, in alignment with those of the Australian Institute of Health and Welfare (AIHW) who observed that paracetamol with codeine was the second most commonly dispensed medicine over a similar timeframe despite recommendations for dentists to prescribe oxycodone as an analgesic instead [25]. Promisingly, the AIHW identified that the number of oxycodone dispensings has increased six‐fold between 2013 and 2024, potentially indicating a shift in analgesic preference in alignment with updated guidelines [25].

In alignment with updated recommendations in the Therapeutic Guidelines, we also observed increased dispensing of temazepam 10 mg, which is commonly used for anxiolysis during dental procedures [23]. Temazepam or lorazepam is preferred over other anxiolytics in the dental setting due to their shorter action [23]. Results of our study did, however, show that the rate of diazepam use increased over the study period despite it being a longer‐acting benzodiazepine and not the anxiolytic of choice in the dental population unless a longer duration of anxiolysis is required [23]. Although the data from this study originate from Australia, several findings have broader international relevance. Inappropriate antibiotic use in dentistry has been recognised as a global antimicrobial stewardship challenge, with dentists contributing a substantial proportion of community antibiotic prescribing in many countries and stewardship interventions being increasingly implemented internationally [26]. Comparative studies have demonstrated important differences in dental antibiotic prescribing patterns across Australia, England, Canada, and the United States, reflecting variation in guideline recommendations and healthcare systems, but with common concerns regarding broad‐spectrum antibiotic use and unnecessary prescribing. Analgesic dispensing patterns differ markedly across countries. For example, opioid prescribing by dentists is substantially more frequent in the United States than in England, where such prescriptions are rare and analgesic practice favours nonopioid therapies, highlighting contrasting approaches to pain management in dental care [7].

Medicine shortages have also emerged as a global health system challenge, particularly following the COVID‐19 pandemic. The World Health Organisation and International Federation of Pharmacists have highlighted increasing medicine shortages and supply chain vulnerabilities as threats to continuity of care and antimicrobial stewardship [9, 27]. Shortages of first‐line agents may necessitate substitution with broader‐spectrum or less desirable alternatives, potentially altering prescribing patterns and increasing the risk of inappropriate antimicrobial exposure. The temporary increases in clindamycin and amoxicillin with clavulanic acid observed during shortages in Australia illustrate mechanisms that are likely to occur in other healthcare systems when preferred therapies become unavailable. Similar concerns regarding shortages leading to therapeutic substitution and disruption of stewardship efforts have been reported internationally [8, 28–30].

Several considerations need to be made when interpreting the results of this study. The dataset utilised in this study only includes PBS‐subsidised medicines and does not include private prescriptions or over‐the‐counter purchases (such as oral antiseptic sprays, oral gargles, or some simple analgesics). Moreover, although temporal associations with external events (for example, COVID‐19 and regulatory changes) were considered, causal inference cannot be established. The specific medicines dispensed and their respective item codes are also only an indicator of the maximum pack size of a product provided to a patient; therefore, no dose inference can be made about how many tablets or a product were supplied to a patient, and subsequently, no inference can be made about treatment duration and adherence to clinical guidelines.

Collectively, these findings support the need for proactive shortage preparedness strategies, particularly for dentists, closer integration of shortage response measures with antimicrobial stewardship frameworks, and regular review of PBS Dental Schedule listings to ensure alignment with contemporary evidence‐based dental practice.

## 5. Conclusion

Dental medicine utilisation in Australia changed substantially between 2014 and 2025, influenced by antimicrobial stewardship initiatives, evolving clinical guidance, and medicine shortages. Antibiotics remained the dominant therapeutic class, with increasing use of amoxicillin–clavulanic acid and recent growth in oxycodone dispensing. Shortages produced temporary shifts toward alternative therapies, highlighting the need for proactive shortage management and continued alignment of PBS dental listings with contemporary evidence‐based practice. These findings have implications beyond Australia, highlighting how medicine shortages and evolving stewardship recommendations can influence dental prescribing patterns and potentially disrupt access to first‐line therapies. Strengthening shortage preparedness and ensuring alignment between formularies and contemporary clinical guidance are likely to be important priorities for dental practice internationally.

## Author Contributions

**Jack Janetzki**: conceptualisation, data curation, formal analysis, investigation, methodology, project administration, resources, visualisation, writing – original draft, writing – reviewing and editing. **Joon Soo Park**: formal analysis, investigation, supervision, writing – reviewing and editing. **Amy Page**: formal analysis, writing – reviewing and editing. **Debra Rowett**: conceptualisation, formal analysis, investigation, methodology, supervision, writing – reviewing and editing. **Wern Chern Chai**: formal analysis, investigation, writing – reviewing and editing. **Lisa Kalisch Ellett**: conceptualisation, formal analysis, investigation, methodology, supervision, writing – reviewing and editing.

## Funding

This study did not receive any specific funding. Open access publishing facilitated by Adelaide University, as part of the Wiley ‐ Adelaide University agreement via the Council of Australasian University Librarians.

## Disclosure

All authors have read and approved the final version of the manuscript. Jack Janetzki had full access to all of the data in this study and takes complete responsibility for the integrity of the data and the accuracy of the data analysis.

## Conflicts of Interest

The authors declare no conflicts of interest.

## Supporting Information

Additional supporting information can be found online in the Supporting Information section.

## Supporting information


**Supporting Information** Appendix Table A: Medicines included in analysis. Name, formulation, and PBS item code. Appendix Table B: Shortage dates of top 10 most commonly dispensed medicines included in the study. Appendix C: Dispensings per 100,000 per month for each medicine other than the top 10 listed in the results section.

## Data Availability

The data that support the findings of this study are openly available in PBS and RPBS Section 85 and Section 100 Date of Supply Data at https://www.pbs.gov.au/info/statistics/dos-and-dop/dos-and-dop.
