# Supplementary material for: Utilisation and Shortages of Dental Medicines in Australia From 2014 to 2025: A Retrospective Time‐Series Study
Source: Int J Dent. 2026 Oct 8;2026:7203795. doi: 10.1155/ijod/7203795 (PMC13647207; doi:10.1155/ijod/7203795)
Supplement: Supplementary file 1 [file IJOD-2026-7203795-s001.docx]

**Appendix:**

*Appendix Table A: Medicines included in analysis. Name, formulation and PBS item code.*

ADRENALINE (EPINEPHRINE) Injection 1 mg (as acid tartrate) in 1 mL (1 in 1,000) 05004J

AMOXICILLIN + CLAVULANIC ACID Powder for oral suspension containing 125 mg amoxicillin (as trihydrate) with 31.25 mg clavulanic acid (as potassium clavulanate) per 5 mL, 75 mL 05009P

AMOXICILLIN + CLAVULANIC ACID Powder for oral suspension containing 400 mg amoxicillin (as trihydrate) with 57 mg clavulanic acid (as potassium clavulanate) per 5 mL, 50 mL (S19A) 13694R

AMOXICILLIN + CLAVULANIC ACID Powder for oral suspension containing 400 mg amoxicillin (as trihydrate) with 57 mg clavulanic acid (as potassium clavulanate) per 5 mL, 60 mL 05011R

AMOXICILLIN + CLAVULANIC ACID Tablet containing 500 mg amoxicillin (as trihydrate) with 125 mg clavulanic acid (as potassium clavulanate) 05008N

AMOXICILLIN + CLAVULANIC ACID Tablet containing 875 mg amoxicillin (as trihydrate) with 125 mg clavulanic acid (as potassium clavulanate) (s19A) 13190F

AMOXICILLIN + CLAVULANIC ACID Tablet containing 875 mg amoxicillin (as trihydrate) with 125 mg clavulanic acid (as potassium clavulanate) 05006L

AMOXICILLIN Capsule 250 mg (as trihydrate) 03301R

AMOXICILLIN Capsule 500 mg (as trihydrate) 03300Q

AMOXICILLIN Powder for oral suspension 125 mg (as trihydrate) per 5 mL, 100 mL 03302T

AMOXICILLIN Powder for oral suspension 250 mg (as trihydrate) per 5 mL, 100 mL (s19A) 13184X

AMOXICILLIN Powder for oral suspension 250 mg (as trihydrate) per 5 mL, 100 mL 03393N

AMOXICILLIN Powder for oral suspension 500 mg (as trihydrate) per 5 mL, 100 mL 05225B

AMOXICILLIN Powder for paediatric oral drops 100 mg (as trihydrate) per mL, 20 mL 03310F

AMPHOTERICIN B Lozenge 10 mg 03306B

AMPICILLIN Powder for injection 1 g (as sodium) 03314K

AMPICILLIN Powder for injection 500 mg (as sodium) 03313J

ASPIRIN Tablet, dispersible, 300 mg 05018D

ATROPINE SULFATE Injection containing atropine sulfate monohydrate 600 micrograms in 1 mL 05022H

BENZATHINE BENZYLPENICILLIN Injection containing 1,200,000 units benzathine benzylpenicillin tetrahydrate in 2.3 mL single use pre-filled syringe 05027N

BENZATHINE BENZYLPENICILLIN Injection containing 600,000 units benzathine benzylpenicillin tetrahydrate in 1.17 mL single use pre-filled syringe 11735P

BENZYDAMINE Mouth and throat rinse containing benzydamine hydrochloride 22.5 mg per 15 mL, 500 mL 05032W

BENZYLPENICILLIN Powder for injection 3 g (as sodium) 03399X

BENZYLPENICILLIN Powder for injection 600 mg (as sodium) 03398W

BETAMETHASONE ACETATE + BETAMETHASONE SODIUM PHOSPHATE Injection containing betamethasone acetate 3 mg with betamethasone sodium phosphate 3.9 mg in 1 mL 05034Y

CARBAMAZEPINE Oral suspension 100 mg per 5 mL, 300 mL 05041H

CARBAMAZEPINE Tablet 100 mg 05039F

CARBAMAZEPINE Tablet 200 mg (controlled release) 05038E

CARBAMAZEPINE Tablet 200 mg (controlled release) 14671E

CARBAMAZEPINE Tablet 200 mg 01724R

CARBAMAZEPINE Tablet 100mg 01755J

CARBAMAZEPINE Tablet 400 mg (controlled release) 05037D

CARBAMAZEPINE Tablet 400 mg (controlled release) 14680P

CEFACLOR Powder for oral suspension 125 mg (as monohydrate) per 5 mL, 100 mL 05046N

CEFACLOR Powder for oral suspension 250 mg (as monohydrate) per 5 mL, 75 mL 05047P

CEFACLOR Tablet (sustained release) 375 mg (as monohydrate) 05045M

CEFALEXIN Capsule 250 mg (as monohydrate) 03317N

CEFALEXIN Capsule 500 mg (as monohydrate) 03318P

CEFALEXIN Granules for oral suspension 125 mg (as monohydrate) per 5 mL, 100 mL 03319Q

CEFALEXIN Granules for oral suspension 250 mg (as monohydrate) per 5 mL, 100 mL 03320R

CEFALOTIN Powder for injection 1 g (as sodium) 03376Q

CEFUROXIME Powder for oral suspension 125 mg (as axetil) per 5 mL, 100 mL 11191B

CEFUROXIME Powder for oral suspension 125 mg (as axetil) per 5 mL, 70 mL (S19A) 13653N

CEFUROXIME Powder for oral suspension 125 mg (as axetil) per 5 mL, 70 mL 02002J

CEFUROXIME Tablet 250 mg (as axetil) 05052X

CEFUROXIME Tablet 250 mg (as axetil) 11228Y

CHLORAMPHENICOL Eye drops 5 mg per mL, 10 mL 05055C

CLINDAMYCIN Capsule 150 mg (as hydrochloride) 05057E

CODEINE Tablet containing codeine phosphate hemihydrate 30 mg 05063L

CODEINE Tablet containing codeine phosphate hemihydrate 30 mg 12054K

DIAZEPAM Injection 10 mg in 2 mL 05073B

DIAZEPAM Tablet 2 mg 05071X

DIAZEPAM Tablet 5 mg 05072Y

DICLOFENAC Suppository containing diclofenac sodium 100 mg 05079H

DICLOFENAC Tablet (enteric coated) containing diclofenac sodium 25 mg 05076E

DICLOFENAC Tablet (enteric coated) containing diclofenac sodium 50 mg 05077F

DICLOXACILLIN Capsule 250 mg (as sodium) 05096F

DICLOXACILLIN Capsule 500 mg (as sodium) 05097G

DOXYCYCLINE Capsule 100 mg (as hyclate) (containing enteric coated pellets) 03322W

DOXYCYCLINE Tablet 100 mg (as hyclate) 03321T

DOXYCYCLINE Tablet 100 mg (as monohydrate) 05082L

ERYTHROMYCIN Capsule 250 mg (containing enteric coated pellets) 03325B

ERYTHROMYCIN ETHYLSUCCINATE Powder for oral liquid 200 mg (as ethyl succinate) per 5 mL, 100 mL 03334L

ERYTHROMYCIN ETHYLSUCCINATE Powder for oral liquid 400 mg (as ethyl succinate) per 5 mL, 100 mL 03337P

ERYTHROMYCIN ETHYLSUCCINATE Tablet 400 mg (as ethyl succinate) 03336N

FLOXACILLIN Capsule 250 mg (as sodium monohydrate) 05090X

FLOXACILLIN Capsule 500 mg (as sodium monohydrate) 05091Y

FLOXACILLIN Capsule 500 mg (as sodium) 11728G

FLOXACILLIN Powder for injection 1 g (as sodium monohydrate) 05095E

FLOXACILLIN Powder for oral liquid 125 mg (as sodium monohydrate) per 5 mL, 100 mL 05257Q

FLOXACILLIN Powder for oral liquid 250 mg (as sodium monohydrate) per 5 mL, 100 mL 05258R

GLUCAGON HYDROCHLORIDE Injection set containing glucagon hydrochloride 1 mg (1 I.U.) and 1 mL solvent in disposable syringe 05105Q

GLUCOSE + SODIUM CHLORIDE I.V. infusion 31 mmol-222 mmol (anhydrous) per L, 1 L 05214K

GLYCERYL TRINITRATE Tablets 600 micrograms, 100 05108W

HYDROCORTISONE ACETATE Cream containing hydrocortisone acetate 10 mg per g, 30 g 05111B

HYDROCORTISONE ACETATE Cream containing hydrocortisone acetate 10 mg per g, 50 g 05113D

HYDROCORTISONE ACETATE Ointment containing hydrocortisone acetate 10 mg per g, 30 g 05112C

HYDROCORTISONE ACETATE Ointment containing hydrocortisone acetate 10 mg per g, 50 g 05114E

HYDROCORTISONE SODIUM SUCCINATE Injection 100 mg (as sodium succinate) with 2 mL solvent 05118J

HYDROMORPHONE Oral liquid containing hydromorphone hydrochloride 1 mg per mL, 473 mL 05132D

HYDROMORPHONE Tablet containing hydromorphone hydrochloride 2 mg 05115F

HYDROMORPHONE Tablet containing hydromorphone hydrochloride 4 mg 05116G

HYDROMORPHONE Tablet containing hydromorphone hydrochloride 8 mg 05117H

IBUPROFEN Tablet 400 mg 05123P

IBUPROFEN Tablet 400 mg 05124Q

INDOMETACIN Capsule 25 mg 05126T

INDOMETACIN Suppository 100 mg 05128X

KETOPROFEN Capsule 200 mg (sustained release) 05136H

LINCOMYCIN Injection 600 mg (as hydrochloride monohydrate) in 2 mL 05144R

LINCOMYCIN Injection 600 mg (as hydrochloride monohydrate) in 2 mL 11366F

METHYLPREDNISOLONE Injection containing methylprednisolone acetate 40 mg in 1 mL 05148Y

METOCLOPRAMIDE Injection containing 10 mg metoclopramide hydrochloride (as monohydrate) in 2 mL 05153F

METOCLOPRAMIDE Tablet containing 10 mg metoclopramide hydrochloride (as monohydrate) 05151D

METRONIDAZOLE I.V. infusion 500 mg in 100 mL 01832K

METRONIDAZOLE I.V. infusion 500 mg in 100 mL 02298Y

METRONIDAZOLE Oral suspension containing metronidazole benzoate 320 mg per 5 mL, 100 mL 03341W

METRONIDAZOLE Suppositories 500 mg, 10 05157K

METRONIDAZOLE Tablet 200 mg 03339R

METRONIDAZOLE Tablet 400 mg 05155H

MORPHINE Injection containing morphine hydrochloride trihydrate 10 mg in 1 mL 10863R

MORPHINE Injection containing morphine hydrochloride trihydrate 20 mg in 1 mL 10858L

MORPHINE Injection containing morphine sulfate pentahydrate 10 mg in 1 mL 05168B

MORPHINE Injection containing morphine sulfate pentahydrate 15 mg in 1 mL 05169C

MORPHINE Injection containing morphine sulfate pentahydrate 30 mg in 1 mL 05170D

MORPHINE Oral solution containing morphine hydrochloride trihydrate 10 mg per mL, 1 mL 05239R

MORPHINE Oral solution containing morphine hydrochloride trihydrate 2 mg per mL, 1 mL 05237P

MORPHINE Oral solution containing morphine hydrochloride trihydrate 5 mg per mL, 1 mL 05238Q

MORPHINE Tablet containing morphine sulfate pentahydrate 30 mg 05163R

NALOXONE Injection containing naloxone hydrochloride 400 micrograms in 1 mL ampoule 10787R

NALOXONE Nasal spray 1.8 mg (as hydrochloride dihydrate) in 0.1 mL single dose unit, 2 11816X

NAPROXEN Tablet 1 g (sustained release) 05179N

NAPROXEN Tablet 250 mg 05176K

NAPROXEN Tablet 500 mg 05177L

NAPROXEN Tablet 750 mg (sustained release) 05178M

NAPROXEN Tablet containing naproxen sodium 550 mg 05186Y

NITRAZEPAM Tablet 5 mg 05189D

NYSTATIN Capsule 500,000 units 03345C

NYSTATIN Oral suspension 100,000 units per mL, 24 mL 03343Y

NYSTATIN Tablet 500,000 units 03342X

OXAZEPAM Tablet 15 mg 05192G

OXAZEPAM Tablet 30 mg 05193H

OXYCODONE Capsule containing oxycodone hydrochloride 10 mg 05197M

OXYCODONE Capsule containing oxycodone hydrochloride 10 mg 12074L

OXYCODONE Capsule containing oxycodone hydrochloride 5 mg 05191F

OXYCODONE Capsule containing oxycodone hydrochloride 5 mg 12025X

OXYCODONE Capsule containing oxycodone hydrochloride 5 mg 12311Y

OXYCODONE Oral solution containing oxycodone hydrochloride 1 mg per mL, 1 mL 05190E

OXYCODONE Suppository 30 mg (as pectinate) 05194J

OXYCODONE Tablet containing oxycodone hydrochloride 5 mg 05195K

OXYCODONE Tablet containing oxycodone hydrochloride 5 mg 12048D

OXYCODONE Tablet containing oxycodone hydrochloride 5 mg 13234M

PARACETAMOL + CODEINE Tablet containing codeine phosphate hemihydrate 30 mg with paracetamol 500 mg 03316M

PARACETAMOL + CODEINE Tablet containing codeine phosphate hemihydrate 30 mg with paracetamol 500 mg 12066C

PARACETAMOL Oral liquid 120 mg per 5 mL, 100 mL 03348F

PARACETAMOL Oral liquid 240 mg per 5 mL, 200 mL 03349G

PARACETAMOL Tablet 500 mg 05196L

PARACETAMOL Tablet 500 mg 05224Y

PHENOXYMETHYLPENICILLIN Capsule 250 mg phenoxymethylpenicillin (as potassium) 03363B

PHENOXYMETHYLPENICILLIN Capsule 500 mg phenoxymethylpenicillin (as potassium) 03364C

PHENOXYMETHYLPENICILLIN Oral suspension 150 mg (as benzathine) per 5 mL, 100 mL 05012T

PHENOXYMETHYLPENICILLIN Powder for oral liquid 125 mg (as potassium) per 5 mL, 100 mL 05024K

PHENOXYMETHYLPENICILLIN Powder for oral liquid 250 mg (as potassium) per 5 mL, 100 mL (s19A) 13291M

PHENOXYMETHYLPENICILLIN Powder for oral liquid 250 mg (as potassium) per 5 mL, 100 mL 05029Q

PHENOXYMETHYLPENICILLIN Tablet 250 mg phenoxymethylpenicillin (as potassium) 03360W

PHENOXYMETHYLPENICILLIN Tablet 500 mg phenoxymethylpenicillin (as potassium) 03361X

PIROXICAM Capsule 10 mg 05203W

PIROXICAM Capsule 20 mg 05204X

PIROXICAM Dispersible tablet 10 mg 05201R

PIROXICAM Dispersible tablet 20 mg 05202T

PROCAINE BENZYLPENICILLIN Injection 1.5 g in disposable syringe 03371K

PROCHLORPERAZINE Injection containing prochlorperazine mesilate 12.5 mg in 1 mL 05206B

PROCHLORPERAZINE Suppositories containing prochlorperazine equivalent to 25 mg prochlorperazine maleate, 5 05208D

PROCHLORPERAZINE Tablet containing prochlorperazine maleate 5 mg 05205Y

PROMETHAZINE Injection containing promethazine hydrochloride 50 mg in 2 mL 03374N

ROXITHROMYCIN Tablet 150 mg 05260W

ROXITHROMYCIN Tablet 300 mg 05261X

ROXITHROMYCIN Tablet for oral suspension 50 mg 05259T

SODIUM CHLORIDE I.V. infusion 154 mmol per L, 1 L 05212H

TEMAZEPAM Tablet 10 mg 05221T

TICARCILLIN Powder for injection 3 g-100 mg (solvent required) 05230G

TRAMADOL Capsule containing tramadol hydrochloride 50 mg 05232J

TRAMADOL Capsule containing tramadol hydrochloride 50 mg 12024W

TRAMADOL Injection containing tramadol hydrochloride 100 mg in 2 mL 05231H

TRAMADOL Oral drops containing tramadol hydrochloride 100 mg per mL, 10 mL 05150C

TRIAMCINOLONE Injection containing triamcinolone acetonide 10 mg in 1 mL 05233K

TRIMETHOPRIM + SULFAMETHOXAZOLE Paediatric oral suspension 40 mg-200 mg per 5 mL, 100 mL 03391L

TRIMETHOPRIM + SULFAMETHOXAZOLE Tablet 160 mg-800 mg 03390K

VANCOMYCIN Powder for injection 1 g (1,000,000 I.U.) (as hydrochloride) 05083M

*Appendix Table B: Shortage dates of top 10 most commonly dispensed medicines included in the study.*

| **ARTG name** | **Active ingredients** | **Dosage form** | **Supply impact start date** | **Supply impact end date** | **Deletion from market** | **Number of days impacted** | **Reason** |
| --- | --- | --- | --- | --- | --- | --- | --- |
| Proladone 30mg | Oxycodone | Suppository, compressed | 29/01/2017 | 30/05/2017 |  | 121 | Manufacturing |
| METRONIDE 400 metronidazole 400mg tablet blister pack | Metronidazole | Tablet, uncoated | 14/04/2017 | 16/06/2017 |  | 63 | Unexpected increase in consumer demand |
| FLAGYL 400mg tablet blister pack | Metronidazole | Tablet, uncoated | 13/02/2017 | 14/07/2017 |  | 151 | Unexpected increase in consumer demand |
| DBL METRONIDAZOLE INTRAVENOUS INFUSION metronidazole 500mg/100mL solution for injection bag | Metronidazole | Injection, solution | 22/02/2017 | 31/10/2017 |  | 251 | Manufacturing |
| DBL DIAZEPAM 10mg/2mL Injection USP | Diazepam | Injection, solution | 4/12/2017 | 28/02/2018 |  | 86 | Manufacturing |
| ALPHACLAV DUO 500/125 amoxicillin 500mg (as trihydrate) & clavulanic acid 125mg (as potassium) tablet blister pack | amoxicillin trihydrate~potassium clavulanate | Tablet, film coated | 30/12/2018 | 20/01/2019 |  | 21 | Other |
| IBILEX 125 cefalexin 125mg/5mL powder for oral liquid bottle | cefalexin | Oral Liquid, powder for | 28/12/2018 | 31/01/2019 |  | 34 | Manufacturing |
| METRONIDAZOLE BAXTER metronidazole 500mg/100mL solution for injection bag | Metronidazole | Injection, solution | 1/01/2019 | 15/02/2019 |  | 45 | Manufacturing |
| AUGMENTIN DUO FORTE tablet blister pack | potassium clavulanate~amoxicillin trihydrate | Tablet, film coated | 6/02/2019 | 15/03/2019 |  | 37 | Manufacturing |
| METRONIDE 400 metronidazole 400 mg tablet blister pack | Metronidazole | Tablet, uncoated | 11/03/2019 | 18/04/2019 |  | 38 | Manufacturing |
| IBILEX 125 cefalexin 125mg/5mL powder for oral liquid bottle | cefalexin | Oral Liquid, powder for | 26/02/2019 | 30/04/2019 |  | 63 | Unexpected increase in consumer demand |
| IBILEX 250 cefalexin 250mg capsule (NF) | cefalexin monohydrate | Capsule, hard | 6/03/2019 | 30/04/2019 |  | 55 | Manufacturing |
| IBILEX 500 cefalexin 500mg capsule (NF) | cefalexin monohydrate | Capsule, hard | 21/03/2019 | 30/04/2019 |  | 40 | Manufacturing |
| APO-CLINDAMYCIN Clindamycin (as hydrochloride) 150 mg capsules blister pack | clindamycin hydrochloride | Capsule, hard | 31/05/2019 | 15/06/2019 |  | 15 | Manufacturing |
| VALPAM 2 diazepam 2mg tablet blister pack | diazepam | Tablet, uncoated | 26/03/2019 | 15/07/2019 |  | 111 | Manufacturing |
| LPV phenoxymethylpenicllin 500mg (as potassium) capsule blister pack | phenoxymethylpenicillin | Capsule, hard | 27/05/2019 | 19/07/2019 |  | 53 | Manufacturing |
| CLINDAMYCIN BNM clindamycin (as hydrochloride) 150mg capsule blister pack | clindamycin hydrochloride | Capsule, hard | 15/02/2019 | 31/07/2019 |  | 166 | Commercial Changes / Commercial viability |
| Antenex 5 tablet bottle | diazepam | Tablet, uncoated | 1/08/2019 | 23/08/2019 |  | 22 | Manufacturing |
| IBILEX 125 cefalexin 125mg/5mL powder for oral liquid bottle | cefalexin monohydrate | Oral Liquid, powder for | 26/07/2019 | 27/09/2019 |  | 63 | Manufacturing |
| IBILEX 250 cefalexin 250mg capsule (NF) | cefalexin monohydrate | Capsule, hard | 12/09/2019 | 30/09/2019 |  | 18 | Other |
| IBILEX 500 cefalexin 500mg capsule (NF) | cefalexin monohydrate | Capsule, hard | 18/09/2019 | 30/09/2019 |  | 12 | Other |
| METROGYL 400 metronidazole 400mg tablet bottle | metronidazole | Tablet, uncoated | 26/09/2019 | 30/09/2019 |  | 4 | Manufacturing |
| AMOXIL SUGAR FREE SYRUP amoxicillin 125 mg/5 mL (as trihydrate) powder for oral liquid bottle | amoxicillin trihydrate | Oral Liquid, powder for | 30/08/2019 | 4/10/2019 |  | 35 | Unexpected increase in consumer demand |
| APO-AMOXYCILLIN amoxicillin (as trihydrate) 125mg/5mL powder for suspension bottle | amoxicillin trihydrate | Suspension, powder for | 12/08/2019 | 7/10/2019 |  | 56 | Unexpected increase in consumer demand |
| MAXAMOX amoxicillin trihydrate 500mg/5mLpowder for oral suspension bottle | amoxicillin trihydrate | Oral Liquid, powder for | 16/10/2019 | 15/11/2019 |  | 30 | Manufacturing |
| IBILEX 125 cefalexin 125mg/5mL powder for oral liquid bottle | cefalexin monohydrate | Oral Liquid, powder for | 10/10/2019 | 30/11/2019 |  | 51 | Manufacturing |
| AMOXIL FORTE SUGAR FREE SYRUP amoxicillin 250 mg/5 mL (as trihydrate) powder for oral liquid bottle | amoxicillin trihydrate | Oral Liquid, powder for | 8/10/2019 | 2/12/2019 |  | 55 | Unexpected increase in consumer demand |
| ALPHACLAV DUO FORTE 875/125 amoxicillin 875 mg (as trihydrate) and clavulanic acid 125 mg (as potassium) tablet blister pack | amoxicillin trihydrate~potassium clavulanate | Tablet, film coated | 11/11/2019 | 31/12/2019 |  | 50 | Manufacturing |
| CEFALEXIN SANDOZ cefalexin (as monohydrate) 125mg/5mL powder for suspension bottle | cefalexin monohydrate | Suspension, powder for | 21/09/2019 | 22/01/2020 |  | 123 | Manufacturing |
| ASPECILLIN VK phenoxymethylpenicillin 500mg (as potassium) tablet bottle | phenoxymethylpenicillin potassium | Tablet, film coated | 7/11/2019 | 31/01/2020 |  | 85 | Manufacturing |
| AMCLAVOX DUO FORTE 875/125, amoxicillin (as trihydrate) 875 mg and clavulanic acid (as potassium) 125 mg tablets blister pack | potassium clavulanate~amoxicillin trihydrate | Tablet, film coated | 18/12/2019 | 13/03/2020 |  | 86 | Other |
| LPV phenoxymethylpenicllin 500mg (as potassium) capsule blister pack | phenoxymethylpenicillin | Capsule, hard | 18/11/2019 | 16/03/2020 |  | 119 | Unexpected increase in consumer demand |
| AMOXYCILLIN SANDOZ amoxicillin trihydrate 250mg/5mL powder for oral suspension bottle | amoxicillin trihydrate | Oral Liquid, powder for | 21/02/2020 | 23/03/2020 |  | 31 | Other |
| CILICAINE VK 500mg capsule blister pack | phenoxymethylpenicillin potassium | Capsule, hard | 10/10/2019 | 31/03/2020 |  | 173 | Manufacturing |
| CILICAINE VK 250mg capsule blister pack | phenoxymethylpenicillin potassium | Capsule, hard | 10/10/2019 | 31/03/2020 |  | 173 | Manufacturing |
| DALACIN C clindamycin (as hydrochloride) 150mg capsule blister pack | clindamycin hydrochloride | Capsule, hard | 10/10/2019 | 17/04/2020 |  | 190 | Manufacturing |
| LPV phenoxymethylpenicillin 250mg (as potassium) capsule blister pack | phenoxymethylpenicillin | Capsule, hard | 30/12/2019 | 20/04/2020 |  | 112 | Unexpected increase in consumer demand |
| CURAM DUO 400/57 amoxicillin 400 mg/5mL (as trihydrate) / clavulanic acid 57 mg/5mL (as potassium clavulanate) powder for suspension bottle | amoxicillin trihydrate~potassium clavulanate | Suspension, powder for | 7/02/2020 | 30/04/2020 |  | 83 | Other |
| AMOXIL SUGAR FREE SYRUP amoxicillin 125 mg/5 mL (as trihydrate) powder for oral liquid bottle | amoxicillin trihydrate | Oral Liquid, powder for | 8/10/2019 | 4/05/2020 |  | 209 | Unexpected increase in consumer demand |
| LPV phenoxymethylpenicllin 500mg (as potassium) capsule blister pack | phenoxymethylpenicillin | Capsule, hard | 1/05/2020 | 15/05/2020 |  | 14 | Other |
| AMOXIL FORTE SUGAR FREE SYRUP amoxicillin 250 mg/5 mL (as trihydrate) powder for oral liquid bottle | amoxicillin trihydrate | Oral Liquid, powder for | 7/04/2020 | 18/05/2020 |  | 41 | Unexpected increase in consumer demand |
| CILAMOX 250mg capsule blister pack | amoxicillin trihydrate | Capsule, hard | 14/10/2019 | 31/05/2020 |  | 230 | Manufacturing |
| CEPHALEXIN GENERICHEALTH cefalexin monohydrate 500 mg capsules blister pack | cefalexin | Capsule | 20/03/2020 | 31/05/2020 |  | 72 | Unexpected increase in consumer demand |
| APO-CEPHALEXIN (as monohydrate) 250mg capsule blister pack | cefalexin monohydrate | Capsule, hard | 27/04/2020 | 8/06/2020 |  | 42 | Other |
| Keflex cefalexin 500mg capsules blister pack | cefalexin monohydrate | Capsule, hard | 22/05/2020 | 12/06/2020 |  | 21 | Unexpected increase in consumer demand |
| ALPHACLAV DUO 500/125 amoxicillin 500mg (as trihydrate) & clavulanic acid 125mg (as potassium) tablet blister pack | potassium clavulanate~amoxicillin trihydrate | Tablet, film coated | 7/04/2020 | 18/06/2020 |  | 72 | Manufacturing |
| CILICAINE VK 500mg capsule blister pack | phenoxymethylpenicillin potassium | Capsule, hard | 22/05/2020 | 19/06/2020 |  | 28 | Unexpected increase in consumer demand |
| APO-AMOXYCILLIN amoxicillin (as trihydrate) 250mg/5mL powder for suspension bottle | amoxicillin trihydrate | Suspension, powder for | 15/06/2020 | 13/07/2020 |  | 28 | Other |
| PANADEINE FORTE paracetamol 500mg and codeine phosphate hemihydrate 30mg tablet blister pack | codeine phosphate hemihydrate~paracetamol | Tablet, uncoated | 17/06/2020 | 31/07/2020 |  | 44 | Unexpected increase in consumer demand |
| CILICAINE VK 250mg capsule blister pack | phenoxymethylpenicillin potassium | Capsule, hard | 8/05/2020 | 31/07/2020 |  | 84 | Unexpected increase in consumer demand |
| AUGMENTIN DUO 400 powder for oral liquid bottle | clavulanic acid~amoxicillin | Oral Liquid, powder for | 7/04/2020 | 30/08/2020 |  | 145 | Unexpected increase in consumer demand |
| CEPHALEXIN GENERICHEALTH cefalexin monohydrate 500 mg capsules blister pack | cefalexin | Capsule | 16/07/2020 | 31/08/2020 |  | 46 | Unexpected increase in consumer demand |
| ASPECILLIN VK phenoxymethylpenicillin 250mg (as potassium) tablet bottle | phenoxymethylpenicillin potassium | Tablet, film coated | 12/12/2019 | 31/08/2020 |  | 263 | Manufacturing |
| ASPECILLIN VK phenoxymethylpenicillin 500mg (as potassium) tablet bottle | phenoxymethylpenicillin potassium | Tablet, film coated | 6/01/2020 | 31/08/2020 |  | 238 | Manufacturing |
| CILICAINE V phenoxymethylpenicillin (as benzathine) 150mg/5mL oral liquid, suspension bottle | phenoxymethylpenicillin benzathine | Oral Liquid, suspension | 25/06/2020 | 4/09/2020 |  | 71 | Manufacturing |
| IBILEX 250 cefalexin 250mg capsule (NF) | cefalexin monohydrate | Capsule, hard | 25/08/2020 | 16/09/2020 |  | 22 | Unexpected increase in consumer demand |
| CILICAINE VK 500mg capsule blister pack | phenoxymethylpenicillin potassium | Capsule, hard | 23/10/2020 | 23/10/2020 |  | 0 | Manufacturing |
| AMOXYCILLIN GENERICHEALTH 500 amoxicillin (as trihydrate) 500 mg capsule blister pack | amoxicillin trihydrate | Capsule, hard | 5/06/2020 | 30/11/2020 |  | 178 | Unexpected increase in consumer demand |
| CEFALEXIN SANDOZ cefalexin monohydrate 250 mg capsules blister pack | cefalexin | Capsule | 30/11/2019 | 18/12/2020 | 12/06/2019 | 384 | Manufacturing |
| ALPHAMOX 250 amoxicillin 250mg/5mL (as trihydrate) powder for oral liquid bottle | amoxicillin trihydrate | Oral Liquid, powder for | 24/01/2019 | 31/12/2020 | 9/07/2020 | 707 | Other |
| ALPHAMOX 125 amoxicillin 125mg/5mL (as trihydrate) powder for oral liquid bottle | amoxicillin trihydrate | Oral Liquid, powder for | 31/01/2019 | 31/12/2020 | 9/07/2020 | 700 | Other |
| AMOXYCLAV GENERICHEALTH 875/125 amoxicillin 875 mg and clavulanic acid 125 mg tablets blister pack | potassium clavulanate~amoxicillin trihydrate | Tablet, film coated | 20/03/2020 | 31/12/2020 |  | 286 | Unexpected increase in consumer demand |
| Antenex 5 tablet bottle | diazepam | Tablet, uncoated | 5/01/2021 | 13/01/2021 |  | 8 | Unexpected increase in consumer demand |
| CILICAINE V phenoxymethylpenicillin (as benzathine) 150mg/5mL oral liquid, suspension bottle | phenoxymethylpenicillin benzathine | Oral Liquid, suspension | 20/02/2020 | 31/01/2021 |  | 346 | Manufacturing |
| VALPAM 5 diazepam 5 mg tablet blister pack | diazepam | Tablet, uncoated | 28/04/2020 | 16/04/2021 |  | 353 | Other |
| CODALGIN FORTE tablet blister pack | paracetamol~codeine phosphate hemihydrate | Tablet, uncoated | 14/10/2020 | 16/04/2021 |  | 184 | Unexpected increase in consumer demand |
| CEFALEXIN SANDOZ cefalexin (as monohydrate) 125mg/5mL powder for suspension bottle | cefalexin | Suspension, powder for | 25/06/2021 | 25/06/2021 |  | 0 | Manufacturing |
| CEFALEXIN SANDOZ cefalexin (as monohydrate) 250mg/5mL powder for suspension bottle | cefalexin | Suspension, powder for | 30/06/2021 | 2/07/2021 |  | 2 | Manufacturing |
| VALPAM 5 diazepam 5 mg tablet blister pack | diazepam | Tablet, uncoated | 18/11/2020 | 2/07/2021 |  | 226 | Manufacturing |
| METROGYL 200 metronidazole 200mg tablet bottle | metronidazole | Tablet, uncoated | 15/06/2021 | 8/07/2021 |  | 23 | Unexpected increase in demand due to other sponsors unable to supply |
| VALPAM 2 diazepam 2 mg tablet blister pack | diazepam | Tablet, uncoated | 15/06/2021 | 9/07/2021 |  | 24 | Manufacturing |
| VALPAM 5 diazepam 5 mg tablet bottle | diazepam | Tablet, uncoated | 18/08/2020 | 16/07/2021 |  | 332 | Manufacturing |
| APO-DIAZEPAM diazepam 5mg tablet blister pack | diazepam | Tablet, uncoated | 23/07/2021 | 30/07/2021 |  | 7 | Commercial Changes / Commercial viability |
| CEPHALEXIN GENERICHEALTH cefalexin monohydrate 500 mg capsules blister pack | cefalexin | Capsule | 9/06/2021 | 17/08/2021 |  | 69 | Manufacturing |
| APO-AMOXYCILLIN amoxicillin (as trihydrate) 125mg/5mL powder for suspension bottle | amoxicillin trihydrate | Suspension, powder for | 1/10/2021 | 1/10/2021 |  | 0 | Manufacturing |
| APO-AMOXYCILLIN amoxicillin (as trihydrate) 125mg/5mL powder for suspension bottle | amoxicillin trihydrate | Suspension, powder for | 5/11/2021 | 5/11/2021 |  | 0 | Manufacturing |
| IBILEX 250 cefalexin 250mg capsule blister pack | cefalexin monohydrate | Capsule, hard | 10/11/2021 | 30/11/2021 |  | 20 | Transport / Logistic issues / Storage capacity issues |
| CILICAINE V phenoxymethylpenicillin (as benzathine) 150mg/5mL oral liquid, suspension bottle | phenoxymethylpenicillin benzathine | Oral Liquid, suspension | 28/09/2021 | 7/12/2021 |  | 70 | Manufacturing |
| METROGYL 200 metronidazole 200mg tablet bottle | metronidazole | Tablet, uncoated | 7/12/2021 | 10/12/2021 |  | 3 | Unexpected increase in demand due to other sponsors unable to supply |
| APX-DIAZEPAM diazepam 5 mg tablet blister pack | diazepam | Tablet, uncoated | 12/11/2021 | 28/01/2022 |  | 77 | Unexpected increase in consumer demand |
| CEPHALEX cefalexin (as monohydrate) 500 mg capsules blister pack | cefalexin monohydrate | Capsule | 10/01/2022 | 2/02/2022 |  | 23 | Manufacturing |
| ASPECILLIN VK phenoxymethylpenicillin 250mg (as potassium) tablet bottle | phenoxymethylpenicillin potassium | Tablet, film coated | 1/02/2022 | 28/02/2022 |  | 27 | Manufacturing |
| ASPECILLIN VK phenoxymethylpenicillin 500mg (as potassium) tablet bottle | phenoxymethylpenicillin potassium | Tablet, film coated | 1/02/2022 | 10/03/2022 |  | 37 | Manufacturing |
| CEPHALEXIN GENERICHEALTH cefalexin monohydrate 500 mg capsules blister pack | cefalexin | Capsule | 21/03/2022 | 7/04/2022 |  | 17 | Manufacturing |
| OXYNDONE oxycodone hydrochloride 5mg film coated tablet blister pack | oxycodone hydrochloride | Tablet, film coated | 1/04/2022 | 6/05/2022 |  | 35 | Manufacturing |
| IBILEX 250 cefalexin 250mg/5mL powder for oral liquid bottle | cefalexin monohydrate | Oral Liquid, powder for | 25/01/2022 | 9/05/2022 |  | 104 | Unexpected increase in demand due to other sponsors unable to supply |
| IBILEX 250 cefalexin 250mg capsule blister pack | cefalexin monohydrate | Capsule, hard | 25/01/2022 | 24/05/2022 |  | 119 | Unexpected increase in demand due to other sponsors unable to supply |
| IBILEX 250 cefalexin 250mg/5mL powder for oral liquid bottle | cefalexin monohydrate | Oral Liquid, powder for | 10/05/2022 | 24/05/2022 |  | 14 | Unexpected increase in consumer demand |
| IBILEX 125 cefalexin 125mg/5mL powder for oral liquid bottle | cefalexin monohydrate | Oral Liquid, powder for | 19/05/2022 | 25/05/2022 |  | 6 | Unexpected increase in demand due to other sponsors unable to supply |
| IBILEX 500 cefalexin 500mg capsule blister pack | cefalexin monohydrate | Capsule, hard | 17/03/2022 | 27/06/2022 |  | 102 | Unexpected increase in demand due to other sponsors unable to supply |
| CEPHALEXIN GENERICHEALTH cefalexin monohydrate 500 mg capsules blister pack | cefalexin | Capsule | 17/06/2022 | 7/07/2022 |  | 20 | Manufacturing |
| METROGYL 200 metronidazole 200mg tablet bottle | metronidazole | Tablet, uncoated | 19/05/2022 | 23/08/2022 |  | 96 | Manufacturing |
| AMOXYCILLIN SANDOZ amoxicillin 500mg (as trihydrate) capsule blister pack | amoxicillin trihydrate | Capsule, hard | 8/07/2022 | 26/08/2022 |  | 49 | Manufacturing |
| AUGMENTIN DUO FORTE tablet blister pack | potassium clavulanate~amoxicillin trihydrate | Tablet, film coated | 12/08/2022 | 9/09/2022 |  | 28 | Unexpected increase in consumer demand |
| AMOXYCLAV GENERICHEALTH 875/125 amoxicillin 875 mg and clavulanic acid 125 mg tablets blister pack | amoxicillin trihydrate~potassium clavulanate | Tablet, film coated | 2/06/2022 | 4/10/2022 |  | 124 | Unexpected increase in consumer demand |
| CURAM DUO FORTE 875/125 amoxicillin 875 mg (as trihydrate) and clavulanic acid 125 mg (as potassium) tablet strip pack | potassium clavulanate~amoxicillin trihydrate | Tablet, film coated | 2/09/2022 | 7/10/2022 |  | 35 | Manufacturing |
| CILICAINE V phenoxymethylpenicillin (as benzathine) 150mg/5mL oral liquid, suspension bottle | phenoxymethylpenicillin benzathine | Oral Liquid, suspension | 1/08/2022 | 10/10/2022 |  | 70 | Unexpected increase in consumer demand |
| APO-AMOXYCILLIN amoxicillin (as trihydrate) 250 mg capsules blister pack | amoxicillin trihydrate | Capsule, hard | 14/10/2022 | 17/10/2022 |  | 3 | Unexpected increase in consumer demand |
| CEPHALEXIN GENERICHEALTH cefalexin monohydrate 500 mg capsules blister pack | cefalexin | Capsule | 21/07/2022 | 20/10/2022 |  | 91 | Unexpected increase in consumer demand |
| ALPHACLAV DUO FORTE 875/125 amoxicillin 875 mg (as trihydrate) and clavulanic acid 125 mg (as potassium) tablet blister pack | amoxicillin trihydrate~potassium clavulanate | Tablet, film coated | 16/06/2022 | 24/10/2022 |  | 130 | Manufacturing |
| ALPHACLAV DUO 500/125 amoxicillin 500mg (as trihydrate) & clavulanic acid 125mg (as potassium) tablet blister pack | potassium clavulanate~amoxicillin trihydrate | Tablet, film coated | 16/06/2022 | 24/10/2022 |  | 130 | Manufacturing |
| APO-CEPHALEXIN (as monohydrate) 250mg capsule blister pack | cefalexin monohydrate | Capsule, hard | 21/10/2022 | 28/10/2022 |  | 7 | Unexpected increase in consumer demand |
| VALIUM diazepam 5 mg tablet blister pack | diazepam | Tablet, uncoated | 29/08/2022 | 28/10/2022 |  | 60 | Manufacturing |
| AMOXYCILLIN SANDOZ amoxicillin 500mg (as trihydrate) capsule blister pack | amoxicillin trihydrate | Capsule, hard | 9/09/2022 | 3/11/2022 |  | 55 | Manufacturing |
| PHENOXYMETHYLPENICILLIN-AFT phenoxymethylpenicillin (as potassium salt) 250mg/5ml powder for oral liquid bottle | phenoxymethylpenicillin potassium | Oral Liquid, powder for | 19/10/2022 | 9/11/2022 |  | 21 | Unexpected increase in consumer demand |
| AMOXIL amoxicillin 500mg (as trihydrate) capsule blister pack | amoxicillin trihydrate | Capsule, hard | 14/11/2022 | 21/11/2022 |  | 7 | Unexpected increase in demand due to other sponsors unable to supply |
| CLINDAMYCIN BNM clindamycin (as hydrochloride) 150 mg capsule blister pack | clindamycin hydrochloride | Capsule, hard | 28/09/2022 | 30/11/2022 |  | 63 | Manufacturing |
| AMOXIL PAEDIATRIC DROPS amoxicillin 100mg/mL (as trihydrate) powder for oral liquid bottle | amoxicillin trihydrate | Oral Liquid, powder for | 14/11/2022 | 2/12/2022 |  | 18 | Unexpected increase in demand due to other sponsors unable to supply |
| FLAGYL metronidazole 400 mg tablet blister pack | metronidazole | Tablet, uncoated | 18/07/2022 | 5/12/2022 |  | 140 | Unexpected increase in demand due to other sponsors unable to supply |
| Alphamox 250 capsule blister pack | amoxicillin trihydrate | Capsule, hard | 25/10/2022 | 13/12/2022 |  | 49 | Unexpected increase in consumer demand |
| ASPECILLIN VK phenoxymethylpenicillin 500mg (as potassium) tablet bottle | phenoxymethylpenicillin potassium | Tablet, film coated | 12/10/2022 | 13/12/2022 |  | 62 | Unexpected increase in consumer demand |
| CEFALEXIN SANDOZ cefalexin (as monohydrate) 250mg/5mL powder for suspension bottle | cefalexin | Suspension, powder for | 25/11/2022 | 16/12/2022 |  | 21 | Manufacturing |
| DBL DIAZEPAM 10mg/2ml injection ampoule | diazepam | Injection, solution | 20/12/2022 | 21/12/2022 |  | 1 | Manufacturing |
| AMOXIL PAEDIATRIC DROPS amoxicillin 100mg/mL (as trihydrate) powder for oral liquid bottle | amoxicillin trihydrate | Oral Liquid, powder for | 13/12/2022 | 9/01/2023 |  | 27 | Commercial Changes / Commercial viability |
| LPV phenoxymethylpenicllin 500mg (as potassium) capsule blister pack | phenoxymethylpenicillin | Capsule, hard | 7/10/2022 | 10/01/2023 |  | 95 | Unexpected increase in demand due to other sponsors unable to supply |
| LPV phenoxymethylpenicillin 250mg (as potassium) capsule blister pack | phenoxymethylpenicillin | Capsule, hard | 20/10/2022 | 10/01/2023 |  | 82 | Unexpected increase in demand due to other sponsors unable to supply |
| AMILOXYN amoxicillin (as trihydrate) 250 mg capsules blister pack | amoxicillin trihydrate | Capsule, hard | 2/12/2022 | 1/02/2023 |  | 61 | Unexpected increase in consumer demand |
| IBILEX 125 cefalexin 125mg/5mL powder for oral liquid bottle | cefalexin monohydrate | Oral Liquid, powder for | 25/11/2022 | 1/02/2023 |  | 68 | Unexpected increase in demand due to other sponsors unable to supply |
| METROGYL 200 metronidazole 200mg tablet bottle | metronidazole | Tablet, uncoated | 16/09/2022 | 9/02/2023 |  | 146 | Manufacturing |
| Alphamox 500 capsule blister pack | amoxicillin trihydrate | Capsule, hard | 17/08/2022 | 22/02/2023 |  | 189 | Unexpected increase in consumer demand |
| APO-AMOXYCILLIN amoxicillin (as trihydrate) 250 mg capsules blister pack | amoxicillin trihydrate | Capsule, hard | 14/11/2022 | 24/02/2023 |  | 102 | Unexpected increase in consumer demand |
| Alphamox 250 capsule blister pack | amoxicillin trihydrate | Capsule, hard | 28/02/2023 | 2/03/2023 |  | 2 | Unexpected increase in consumer demand |
| AMOXYCLAV GENERICHEALTH 875/125 amoxicillin 875 mg and clavulanic acid 125 mg tablets blister pack | amoxicillin trihydrate~potassium clavulanate | Tablet, film coated | 17/10/2022 | 2/03/2023 |  | 136 | Manufacturing |
| AMOXYCILLIN GENERICHEALTH 500 amoxicillin (as trihydrate) 500 mg capsule blister pack | amoxicillin trihydrate | Capsule, hard | 30/09/2022 | 3/03/2023 |  | 154 | Unexpected increase in consumer demand |
| CILICAINE VK 250mg capsule blister pack | phenoxymethylpenicillin potassium | Capsule, hard | 4/11/2022 | 8/03/2023 |  | 124 | Manufacturing |
| ASPECILLIN VK phenoxymethylpenicillin 500mg (as potassium) tablet bottle | phenoxymethylpenicillin potassium | Tablet, film coated | 30/01/2023 | 8/03/2023 |  | 37 | Manufacturing |
| CILICAINE VK 500mg capsule blister pack | phenoxymethylpenicillin potassium | Capsule, hard | 28/02/2023 | 8/03/2023 |  | 8 | Manufacturing |
| FLAGYL S benzoyl metronidazole 64 mg/mL suspension oral liquid bottle | metronidazole benzoate | Oral Liquid, suspension | 26/10/2022 | 22/03/2023 |  | 147 | Unexpected increase in consumer demand |
| AMOXIL FORTE SUGAR FREE SYRUP amoxicillin 250 mg/5 mL (as trihydrate) powder for oral liquid bottle | amoxicillin trihydrate | Oral Liquid, powder for | 12/08/2022 | 14/04/2023 |  | 245 | Unexpected increase in demand due to other sponsors unable to supply |
| CEPHALEXIN GENERICHEALTH cefalexin monohydrate 500 mg capsules blister pack | cefalexin | Capsule | 25/10/2022 | 20/04/2023 |  | 177 | Unexpected increase in consumer demand |
| APX-DIAZEPAM diazepam 5 mg tablet blister pack | diazepam | Tablet, uncoated | 24/04/2023 | 24/04/2023 |  | 0 | Manufacturing |
| AMOXIL SUGAR FREE SYRUP amoxicillin 125 mg/5 mL (as trihydrate) powder for oral liquid bottle | amoxicillin trihydrate | Oral Liquid, powder for | 14/11/2022 | 25/04/2023 |  | 162 | Unexpected increase in demand due to other sponsors unable to supply |
| IBILEX 500 cefalexin 500mg capsule blister pack | cefalexin monohydrate | Capsule, hard | 31/03/2023 | 3/05/2023 |  | 33 | Unexpected increase in consumer demand |
| APO-AMOXYCILLIN amoxicillin (as trihydrate) 125mg/5mL powder for suspension bottle | amoxicillin trihydrate | Suspension, powder for | 7/10/2022 | 5/05/2023 |  | 210 | Unexpected increase in consumer demand |
| AMILOXYN amoxicillin (as trihydrate) 500 mg capsules blister pack | amoxicillin trihydrate | Capsule, hard | 10/10/2022 | 5/05/2023 |  | 207 | Unexpected increase in consumer demand |
| APO-CEPHALEXIN cefalexin 500mg capsule blister pack | cefalexin monohydrate | Capsule, hard | 12/05/2023 | 12/05/2023 |  | 0 | Unexpected increase in consumer demand |
| ASPECILLIN VK phenoxymethylpenicillin 250mg (as potassium) tablet bottle | phenoxymethylpenicillin potassium | Tablet, film coated | 28/11/2022 | 24/05/2023 |  | 177 | Manufacturing |
| APO-AMOXY/CLAV 875/125 amoxicillin 875 mg and clavulanic acid 125 mg tablets blister pack | potassium clavulanate~amoxicillin trihydrate | Tablet, film coated | 26/05/2023 | 2/06/2023 |  | 7 | Unexpected increase in consumer demand |
| ALPHACLAV DUO 500/125 amoxicillin 500mg (as trihydrate) & clavulanic acid 125mg (as potassium) tablet blister pack | amoxicillin trihydrate~potassium clavulanate | Tablet, film coated | 8/02/2023 | 8/06/2023 |  | 120 | Unexpected increase in demand due to other sponsors unable to supply |
| CURAM 125/31.25 amoxicillin (as trihydrate) 125mg/5mL clavulanic acid (as potassium clavulanate) 31.25mg/5mL powder for suspension bottle | potassium clavulanate~amoxicillin trihydrate | Suspension, powder for | 4/11/2022 | 9/06/2023 |  | 217 | Manufacturing |
| AMCLAVOX DUO 500/125, amoxicillin (as trihydrate) 500 mg and clavulanic acid (as potassium) 125 mg tablets blister pack | potassium clavulanate~amoxicillin trihydrate | Tablet, film coated | 4/03/2022 | 23/06/2023 | 21/06/2023 | 476 | Manufacturing |
| AUGMENTIN DUO 400 powder for oral liquid bottle | amoxicillin~clavulanic acid | Oral Liquid, powder for | 13/12/2022 | 26/06/2023 |  | 195 | Commercial Changes / Commercial viability |
| LPV phenoxymethylpenicillin 250mg (as potassium) capsule blister pack | phenoxymethylpenicillin | Capsule, hard | 1/05/2023 | 26/06/2023 |  | 56 | Unexpected increase in consumer demand |
| AMOXIL PAEDIATRIC DROPS amoxicillin 100mg/mL (as trihydrate) powder for oral liquid bottle | amoxicillin trihydrate | Oral Liquid, powder for | 12/05/2023 | 30/06/2023 |  | 49 | Commercial Changes / Commercial viability |
| CILICAINE V phenoxymethylpenicillin (as benzathine) 150mg/5mL oral liquid, suspension bottle | phenoxymethylpenicillin benzathine | Oral Liquid, suspension | 12/12/2022 | 30/06/2023 |  | 200 | Manufacturing |
| AUGMENTIN DUO amoxicillin 500mg (as trihydrate) & clavulanic acid 125mg (as potassium clavulanate) | clavulanic acid~amoxicillin | Tablet, film coated | 14/11/2022 | 1/07/2023 |  | 229 | Unexpected increase in demand due to other sponsors unable to supply |
| AMOXIL amoxicillin 250mg (as trihydrate) capsule blister pack | amoxicillin trihydrate | Capsule, hard | 12/05/2023 | 18/07/2023 |  | 67 | Unexpected increase in demand due to other sponsors unable to supply |
| CEFALEXIN SANDOZ cefalexin (as monohydrate) 250mg/5mL powder for suspension bottle | cefalexin | Suspension, powder for | 3/02/2023 | 28/07/2023 |  | 175 | Manufacturing |
| CILICAINE VK 500mg capsule blister pack | phenoxymethylpenicillin potassium | Capsule, hard | 13/04/2023 | 10/08/2023 |  | 119 | Manufacturing |
| AMOXYCLAV GENERICHEALTH 875/125 amoxicillin 875 mg and clavulanic acid 125 mg tablets blister pack | amoxicillin trihydrate~potassium clavulanate | Tablet, film coated | 27/03/2023 | 18/08/2023 |  | 144 | Unexpected increase in consumer demand |
| DALACIN C clindamycin (as hydrochloride) 150mg capsule blister pack | clindamycin hydrochloride | Capsule, hard | 18/08/2023 | 18/08/2023 |  | 0 | Manufacturing |
| PHENOXYMETHYLPENICILLIN-AFT phenoxymethylpenicillin (as potassium salt) 250mg/5ml powder for oral liquid bottle | phenoxymethylpenicillin potassium | Oral Liquid, powder for | 5/12/2022 | 6/09/2023 |  | 275 | Manufacturing |
| PHENOXYMETHYLPENICILLIN-AFT phenoxymethylpenicillin (as potassium salt) 125mg/5ml powder for oral liquid bottle | phenoxymethylpenicillin potassium | Oral Liquid, powder for | 9/03/2023 | 6/09/2023 |  | 181 | Manufacturing |
| AMOXYCILLIN SANDOZ amoxicillin 500mg (as trihydrate) capsule blister pack | amoxicillin trihydrate | Capsule, hard | 9/12/2022 | 8/09/2023 |  | 273 | Manufacturing |
| AUGMENTIN DUO 400 powder for oral liquid bottle | clavulanic acid~amoxicillin | Oral Liquid, powder for | 18/07/2023 | 20/09/2023 |  | 64 | Manufacturing |
| CURAM DUO FORTE 875/125 amoxicillin 875 mg (as trihydrate) and clavulanic acid 125 mg (as potassium) tablet strip pack | amoxicillin trihydrate~potassium clavulanate | Tablet, film coated | 13/01/2023 | 29/09/2023 |  | 259 | Manufacturing |
| CURAM DUO 500/125 amoxicillin 500mg (as trihydrate) & clavulanic acid 125mg (as potassium) tablet blister pack | amoxicillin trihydrate~potassium clavulanate | Tablet, film coated | 27/01/2023 | 29/09/2023 |  | 245 | Manufacturing |
| APO-CEPHALEXIN cefalexin 500mg capsule blister pack | cefalexin monohydrate | Capsule, hard | 7/07/2023 | 29/09/2023 |  | 84 | Unexpected increase in consumer demand |
| CURAM DUO 400/57 amoxicillin 400 mg/5mL (as trihydrate) / clavulanic acid 57 mg/5mL (as potassium clavulanate) powder for suspension bottle | potassium clavulanate~amoxicillin trihydrate | Suspension, powder for | 28/10/2022 | 29/09/2023 |  | 336 | Manufacturing |
| IBILEX 125 cefalexin 125mg/5mL powder for oral liquid bottle | cefalexin monohydrate | Oral Liquid, powder for | 27/03/2023 | 5/10/2023 |  | 192 | Unexpected increase in consumer demand |
| APX-DIAZEPAM diazepam 5 mg tablet blister pack | diazepam | Tablet, uncoated | 30/06/2023 | 3/11/2023 |  | 126 | Unexpected increase in consumer demand |
| AMOXYCILLIN SANDOZ amoxicillin trihydrate 250mg/5mL powder for oral suspension bottle | amoxicillin trihydrate | Oral Liquid, powder for | 5/08/2022 | 1/12/2023 |  | 483 | Manufacturing |
| CURAM DUO FORTE 875/125 amoxicillin 875 mg (as trihydrate) and clavulanic acid 125 mg (as potassium) tablet blister pack | amoxicillin trihydrate~potassium clavulanate | Tablet, film coated | 11/01/2023 | 1/12/2023 |  | 324 | Manufacturing |
| CEFALEXIN SANDOZ cefalexin (as monohydrate) 500mg capsule blister pack | cefalexin monohydrate | Capsule, hard | 7/10/2022 | 1/12/2023 |  | 420 | Manufacturing |
| APO-CLINDAMYCIN Clindamycin (as hydrochloride) 150 mg capsules blister pack | clindamycin hydrochloride | Capsule, hard | 2/06/2023 | 8/12/2023 |  | 189 | Unexpected increase in consumer demand |
| DBL DIAZEPAM 10mg/2ml injection ampoule | diazepam | Injection, solution | 17/12/2023 | 18/12/2023 |  | 1 | Transport / Logistic issues / Storage capacity issues |
| APX-AMOXICILLIN/CLAVULANIC ACID 500/125 amoxicillin 500 mg (as trihydrate) & clavulanic acid 125 mg (as potassium) tablet strip pack | amoxicillin trihydrate~potassium clavulanate | Tablet, film coated | 16/11/2022 | 22/12/2023 |  | 401 | Manufacturing |
| APO-AMOXY/CLAV 875/125 amoxicillin 875 mg and clavulanic acid 125 mg tablets blister pack | potassium clavulanate~amoxicillin trihydrate | Tablet, film coated | 8/09/2023 | 22/12/2023 |  | 105 | Unexpected increase in consumer demand |
| KEFLEX cefalexin 250mg/5mL powder for oral liquid bottle | cefalexin | Oral Liquid, powder for | 22/06/2023 | 10/01/2024 |  | 202 | Manufacturing |
| AMOXYCILLIN SANDOZ amoxicillin (as trihydrate) 1000 mg tablet blister pack | amoxicillin trihydrate | Tablet, film coated | 4/11/2022 | 15/01/2024 |  | 437 | Manufacturing |
| METROGYL 400 metronidazole 400mg tablet bottle | metronidazole | Tablet, uncoated | 25/01/2024 | 25/01/2024 |  | 0 | Manufacturing |
| CURAM DUO 400/57 amoxicillin 400 mg/5mL (as trihydrate) / clavulanic acid 57 mg/5mL (as potassium clavulanate) powder for suspension bottle | potassium clavulanate~amoxicillin trihydrate | Suspension, powder for | 18/12/2023 | 26/01/2024 |  | 39 | Manufacturing |
| AMOXYCLAV GENERICHEALTH 875/125 amoxicillin 875 mg and clavulanic acid 125 mg tablets blister pack | potassium clavulanate~amoxicillin trihydrate | Tablet, film coated | 15/09/2023 | 13/02/2024 |  | 151 | Unexpected increase in consumer demand |
| METRONIDAZOLE KABI metronidazole 500mg/100mL solution for injection bottle | metronidazole | Injection, solution | 13/02/2024 | 26/02/2024 |  | 13 | Transport / Logistic issues / Storage capacity issues |
| KEFLEX cefalexin 125mg/5mL powder for oral liquid bottle | cefalexin | Oral Liquid, powder for | 22/06/2023 | 11/04/2024 |  | 294 | Manufacturing |
| VALPAM 5 diazepam 5 mg tablet bottle | diazepam | Tablet, uncoated | 22/03/2024 | 12/04/2024 |  | 21 | Transport / Logistic issues / Storage capacity issues |
| CEFALEXIN SANDOZ cefalexin (as monohydrate) 125mg/5mL powder for suspension bottle | cefalexin | Suspension, powder for | 13/01/2023 | 3/05/2024 |  | 476 | Manufacturing |
| VALPAM 5 diazepam 5 mg tablet blister pack | diazepam | Tablet, uncoated | 2/02/2024 | 3/05/2024 |  | 91 | Manufacturing |
| METRONIDAZOLE KABI metronidazole 500mg/100mL solution for injection bottle | metronidazole | Injection, solution | 6/05/2024 | 14/05/2024 |  | 8 | Transport / Logistic issues / Storage capacity issues |
| MAXAMOX amoxicillin trihydrate 500mg/5mLpowder for oral suspension bottle | amoxicillin trihydrate | Oral Liquid, powder for | 29/07/2022 | 4/06/2024 |  | 676 | Manufacturing |
| OXYNORM oxycodone hydrochloride 5mg capsule blister pack | oxycodone hydrochloride | Capsule, hard | 21/06/2024 | 21/06/2024 |  | 0 | Commercial Changes / Commercial viability |
| IBILEX 125 cefalexin 125mg/5mL powder for oral liquid bottle | cefalexin monohydrate | Oral Liquid, powder for | 10/05/2024 | 15/07/2024 |  | 66 | Manufacturing |
| OXYCODONE BNM oxycodone hydrochloride 10mg capsule blister pack | oxycodone hydrochloride | Capsule, hard | 8/01/2023 | 16/08/2024 |  | 586 | Commercial Changes / Commercial viability |
| OXYCODONE BNM oxycodone hydrochloride 5mg capsule blister pack | oxycodone hydrochloride | Capsule, hard | 31/01/2023 | 16/08/2024 |  | 563 | Commercial Changes / Commercial viability |
| METROGYL 400 metronidazole 400mg tablet bottle | metronidazole | Tablet, uncoated | 18/09/2024 | 26/09/2024 |  | 8 | Manufacturing |
| APO-AMOXY/CLAV 875/125 amoxicillin 875 mg and clavulanic acid 125 mg tablets blister pack | amoxicillin trihydrate~potassium clavulanate | Tablet, film coated | 14/07/2024 | 2/10/2024 |  | 80 | Manufacturing |
| MAXAMOX amoxicillin trihydrate 500mg/5mLpowder for oral suspension bottle | amoxicillin trihydrate | Oral Liquid, powder for | 5/09/2024 | 16/10/2024 |  | 41 | Manufacturing |
| OXYNORM oxycodone hydrochloride 10mg capsule blister pack | oxycodone hydrochloride | Capsule, hard | 18/06/2024 | 25/10/2024 |  | 129 | Commercial Changes / Commercial viability |
| OXYNORM oxycodone hydrochloride 5mg capsule blister pack | oxycodone hydrochloride | Capsule, hard | 25/10/2024 | 25/10/2024 |  | 0 | Manufacturing |
| CURAM 125/31.25 amoxicillin (as trihydrate) 125mg/5mL clavulanic acid (as potassium clavulanate) 31.25mg/5mL powder for suspension bottle | potassium clavulanate~amoxicillin trihydrate | Suspension, powder for | 27/10/2023 | 21/11/2024 |  | 391 | Manufacturing |
| Antenex 2 tablet bottle | diazepam | Tablet, uncoated | 29/11/2024 | 18/12/2024 |  | 19 | Manufacturing |
| OXYNORM LIQUID oxycodone hydrochloride 1mg/mL oral liquid bottle | oxycodone hydrochloride | Oral Liquid, solution | 31/10/2024 | 19/12/2024 |  | 49 | Unexpected increase in consumer demand |
| OXYNORM LIQUID oxycodone hydrochloride 1mg/mL oral liquid bottle | oxycodone hydrochloride | Oral Liquid, solution | 14/01/2025 | 10/02/2025 |  | 27 | Unexpected increase in consumer demand |
| ASPECILLIN VK phenoxymethylpenicillin 250mg (as potassium) tablet bottle | phenoxymethylpenicillin potassium | Tablet, film coated | 26/11/2024 | 19/02/2025 |  | 85 | Manufacturing |
| ASPECILLIN VK phenoxymethylpenicillin 250mg (as potassium) tablet bottle | phenoxymethylpenicillin potassium | Tablet, film coated | 4/03/2025 | 4/03/2025 |  | 0 | Manufacturing |
| ASPECILLIN VK phenoxymethylpenicillin 500mg (as potassium) tablet bottle | phenoxymethylpenicillin potassium | Tablet, film coated | 4/03/2025 | 4/03/2025 |  | 0 | Manufacturing |
| CILICAINE VK 500mg capsule blister pack | phenoxymethylpenicillin potassium | Capsule, hard | 4/03/2025 | 4/03/2025 |  | 0 | Manufacturing |
| CILICAINE VK 250mg capsule blister pack | phenoxymethylpenicillin potassium | Capsule, hard | 4/03/2025 | 4/03/2025 |  | 0 | Manufacturing |
| APO-CEPHALEXIN (as monohydrate) 250mg capsule blister pack | cefalexin monohydrate | Capsule, hard | 12/08/2025 | 12/08/2025 |  | 0 | Manufacturing |
| OXYCODONE BNM oxycodone hydrochloride 10mg capsule blister pack | oxycodone hydrochloride | Capsule, hard | 18/10/2024 | 19/08/2025 |  | 305 | Commercial Changes / Commercial viability |
| METRONIDAZOLE KABI metronidazole 500mg/100mL solution for injection bottle | metronidazole | Injection, solution | 27/08/2025 | 15/09/2025 |  | 19 | Transport / Logistic issues / Storage capacity issues |
| PHENOXYMETHYLPENICILLIN-AFT phenoxymethylpenicillin (as potassium salt) 125mg/5ml powder for oral liquid bottle | phenoxymethylpenicillin potassium | Oral Liquid, powder for | 9/09/2025 | 29/10/2025 |  | 50 | Manufacturing |
| ASPECILLIN VK phenoxymethylpenicillin 500mg (as potassium) tablet bottle | phenoxymethylpenicillin potassium | Tablet, film coated | 12/09/2025 | 24/11/2025 |  | 73 | Manufacturing |
| METRONIDAMED metronidazole 400 mg tablet blister pack | metronidazole | Tablet, film coated | 14/10/2025 | 12/12/2025 |  | 59 | Unexpected increase in demand due to other sponsors unable to supply |
| CLINDAMYCIN-WGR clindamycin (as hydrochloride) 150 mg capsule blister pack | clindamycin hydrochloride | Capsule, hard | 18/12/2025 | 18/12/2025 |  | 0 | Manufacturing |
| AMOXYCILLIN SANDOZ amoxicillin 250mg (as trihydrate) capsule blister pack | amoxicillin trihydrate | Capsule, hard | 30/11/2019 |  | 1/03/2020 |  | Other |
| CILAMOX 500mg capsule blister pack | amoxicillin trihydrate | Capsule, hard | 31/08/2022 |  | 30/11/2022 |  | Commercial Changes / Commercial viability |
| CILAMOX 250mg capsule blister pack | amoxicillin trihydrate | Capsule, hard | 31/10/2022 |  | 31/01/2023 |  | Commercial Changes / Commercial viability |
| AMOXYCILLIN/CLAVULANIC ACID 500/125 APOTEX amoxicillin 500mg (as trihydrate) & clavulanic acid 125mg (as potassium) tablet blister pack | amoxicillin trihydrate~potassium clavulanate | Tablet, film coated | 2/11/2022 |  | 18/05/2023 |  | Commercial Changes / Commercial viability |
| ALPHACLAV DUO FORTE 875/125 amoxicillin 875 mg (as trihydrate) and clavulanic acid 125 mg (as potassium) tablet blister pack | amoxicillin trihydrate~potassium clavulanate | Tablet, film coated | 30/12/2022 |  | 7/09/2023 |  | Unexpected increase in demand due to other sponsors unable to supply |
| APX-AMOXICILLIN/CLAVULANIC ACID 500/125 amoxicllin 500 mg (as trihydrate) & clavulanic acid 125 mg (as potassium) tablet strip pack | amoxicillin trihydrate~potassium clavulanate | Tablet, film coated | 1/11/2024 |  | 1/11/2024 |  | Commercial Changes / Commercial viability |
| CHEMMART CLINDAMYCIN 150 mg Clindamycin (as hydrochloride) 150 mg capsules blister pack | clindamycin hydrochloride | Capsule, hard | 26/05/2019 |  | 26/05/2019 |  | Commercial Changes / Commercial viability |
| TERRYWHITE CHEMISTS Clindamycin 150 mg Clindamycin (as hydrochloride) 150 mg capsules blister pack | clindamycin hydrochloride | Capsule, hard | 26/05/2019 |  | 26/05/2019 |  | Commercial Changes / Commercial viability |
| CLINDAMYCIN BNM clindamycin (as hydrochloride) 150 mg capsule blister pack | clindamycin hydrochloride | Capsule, hard | 31/07/2024 |  | 1/08/2024 |  | Commercial Changes / Commercial viability |
| VALIUM 5mg tablet | Diazepam | Tablet, uncoated | 31/07/2014 |  |  |  | Discontinuation |
| Valium tablet 2mg | Diazepam | Tablet, uncoated | 1/12/2014 |  | 3/07/2014 |  | Commercial Changes / Commercial viability |
| APO-DIAZEPAM diazepam 5mg tablet blister pack | diazepam | Tablet, uncoated | 11/03/2022 |  | 11/03/2022 |  | Commercial Changes / Commercial viability |
| APO-DIAZEPAM diazepam 2mg tablet blister pack | diazepam | Tablet, uncoated | 12/08/2022 |  | 12/08/2022 |  | Commercial Changes / Commercial viability |
| DBL METRONIDAZOLE INTRAVENOUS INFUSION metronidazole 500mg/100mL solution for injection bag | Metronidazole | Injection, solution | 20/04/2018 |  | 1/04/2020 |  | Manufacturing |
| METRONIDAZOLE-CLARIS metronidazole 500mg/100mL solution for injection vial | Metronidazole | Injection, solution | 1/01/2019 |  | 9/01/2019 |  | Other |
| METRONIDE 400 metronidazole 400 mg tablet blister pack | metronidazole | Tablet, uncoated | 7/02/2022 |  | 20/01/2022 |  | Commercial Changes / Commercial viability |
| FLAGYL metronidazole 200 mg tablet blister pack | metronidazole | Tablet, uncoated | 17/06/2022 |  | 23/05/2022 |  | Commercial Changes / Commercial viability |
| METRONIDE 200 metronidazole 200 mg tablet blister pack | metronidazole | Tablet, uncoated | 31/10/2022 |  | 26/09/2022 |  | Commercial Changes / Commercial viability |
| Pfizer (Australia) METRONIDAZOLE 5mg/mL (0.5%) injection vial | metronidazole | Injection, solution | 1/03/2023 |  | 1/03/2023 |  | Commercial Changes / Commercial viability |
| APO-PARACETAMOL/CODEINE 500/30 paracetamol / codeine phosphate hemihydrate 500mg / 30mg tablets blister pack | paracetamol~codeine phosphate hemihydrate | Tablet | 8/09/2020 |  | 8/09/2020 |  | Commercial Changes / Commercial viability |
| CILICAINE VK 500mg capsule blister pack | phenoxymethylpenicillin potassium | Capsule, hard | 28/01/2021 |  | 25/02/2021 |  | Commercial Changes / Commercial viability |
| AMOXICLAV AMNEAL 875/125 amoxicillin 875 mg and clavulanic acid 125 mg tablets blister pack | potassium clavulanate~amoxicillin trihydrate | Tablet, film coated | 18/02/2019 |  | 30/06/2019 |  | Commercial Changes / Commercial viability |

*Appendix C: Dispensings per 100,000 per month for each medicine other than the top 10 listed in the results section.*

Adrenaline

|  | Epinephrine injection 1 mg in 1 mL (1 in 1,000) 05004J |
| --- | --- |
| 2014-02-01 | 0.0128171 |
| 2014-03-01 | 0.00427237 |
| 2014-04-01 | 0.00425973 |
| 2014-05-01 | 0.0383375 |
| 2014-06-01 | 0.00851945 |
| 2014-07-01 | 0.0212198 |
| 2014-08-01 | 0.0212198 |
| 2014-09-01 | 0.00848792 |
| 2014-10-01 | 0.0126902 |
| 2014-12-01 | 0.0253804 |
| 2015-01-01 | 0.0168452 |
| 2015-03-01 | 0.0042113 |
| 2015-04-01 | 0.00839772 |
| 2015-05-01 | 0.00839772 |
| 2015-06-01 | 0.00419886 |
| 2015-07-01 | 0.00418335 |
| 2015-08-01 | 0.00836671 |
| 2015-09-01 | 0.0167334 |
| 2015-10-01 | 0.00833869 |
| 2015-11-01 | 0.00416935 |
| 2015-12-01 | 0.012508 |
| 2016-01-01 | 0.00414879 |
| 2016-02-01 | 0.00829758 |
| 2016-03-01 | 0.00414879 |
| 2016-04-01 | 0.0165351 |
| 2016-05-01 | 0.00826757 |
| 2016-07-01 | 0.00823129 |
| 2016-08-01 | 0.0164626 |
| 2016-09-01 | 0.0164626 |
| 2016-10-01 | 0.0123026 |
| 2016-11-01 | 0.00820174 |
| 2016-12-01 | 0.0205044 |
| 2017-01-01 | 0.00815947 |
| 2017-02-01 | 0.0122392 |
| 2017-03-01 | 0.00815947 |
| 2017-04-01 | 0.0121988 |
| 2017-05-01 | 0.0121988 |
| 2017-06-01 | 0.0203313 |
| 2017-07-01 | 0.00405021 |
| 2017-09-01 | 0.00810042 |
| 2017-10-01 | 0.0121168 |
| 2017-11-01 | 0.00807786 |
| 2017-12-01 | 0.00807786 |
| 2018-01-01 | 0.016076 |
| 2018-03-01 | 0.012057 |
| 2018-05-01 | 0.0200294 |
| 2018-06-01 | 0.00801177 |
| 2018-07-01 | 0.00797849 |
| 2018-08-01 | 0.00398925 |
| 2018-09-01 | 0.0239355 |
| 2018-11-01 | 0.0119303 |
| 2018-12-01 | 0.00397675 |
| 2019-01-01 | 0.0158322 |
| 2019-02-01 | 0.0118742 |
| 2019-03-01 | 0.00791611 |
| 2019-04-01 | 0.0118414 |
| 2019-05-01 | 0.00394714 |
| 2019-07-01 | 0.0157245 |
| 2019-09-01 | 0.00786223 |
| 2019-11-01 | 0.00391842 |
| 2020-01-01 | 0.011706 |
| 2020-04-01 | 0.00389875 |
| 2020-05-01 | 0.0077975 |
| 2020-06-01 | 0.0116962 |
| 2020-07-01 | 0.00390117 |
| 2020-08-01 | 0.00390117 |
| 2020-09-01 | 0.00390117 |
| 2020-10-01 | 0.0156063 |
| 2021-01-01 | 0.00779636 |
| 2021-03-01 | 0.0194909 |
| 2021-04-01 | 0.00389326 |
| 2021-05-01 | 0.00778652 |
| 2021-06-01 | 0.00389326 |
| 2021-10-01 | 0.00387991 |
| 2021-12-01 | 0.00387991 |
| 2022-01-01 | 0.00771814 |
| 2022-02-01 | 0.00771814 |
| 2022-03-01 | 0.00385907 |
| 2022-05-01 | 0.00384339 |
| 2022-06-01 | 0.0115302 |
| 2022-07-01 | 0.0114638 |
| 2022-08-01 | 0.00382127 |
| 2022-09-01 | 0.00382127 |
| 2022-11-01 | 0.00379935 |
| 2022-12-01 | 0.0075987 |
| 2023-01-01 | 0.0150862 |
| 2023-02-01 | 0.0075431 |
| 2023-03-01 | 0.0075431 |
| 2023-05-01 | 0.0075019 |
| 2023-07-01 | 0.011181 |
| 2023-09-01 | 0.00372701 |
| 2023-10-01 | 0.00370966 |
| 2023-11-01 | 0.00741932 |
| 2023-12-01 | 0.00370966 |
| 2024-01-01 | 0.00737639 |
| 2024-02-01 | 0.00737639 |
| 2024-04-01 | 0.0110319 |
| 2024-06-01 | 0.00367731 |
| 2024-07-01 | 0.00366253 |
| 2024-08-01 | 0.00366253 |
| 2024-09-01 | 0.00732507 |
| 2024-10-01 | 0.0146025 |
| 2024-11-01 | 0.00730123 |
| 2024-12-01 | 0.00365062 |
| 2025-01-01 | 0.00363149 |
| 2025-03-01 | 0.00363149 |
| 2025-05-01 | 0.0108945 |
| 2025-08-01 | 0.00726299 |

Amphotercin B

|  | Amphotericin lozenge 10 mg 03306B |
| --- | --- |
| 2014-02-01 | 1.07664 |
| 2014-03-01 | 1.07664 |
| 2014-04-01 | 1.07345 |
| 2014-05-01 | 1.1842 |
| 2014-06-01 | 1.20976 |
| 2014-07-01 | 1.1586 |
| 2014-08-01 | 1.15436 |
| 2014-09-01 | 1.1586 |
| 2014-10-01 | 1.30709 |
| 2014-11-01 | 1.1675 |
| 2014-12-01 | 1.28594 |
| 2015-01-01 | 0.947543 |
| 2015-02-01 | 1.09073 |
| 2015-03-01 | 1.10757 |
| 2015-04-01 | 1.08331 |
| 2015-05-01 | 1.17148 |
| 2015-06-01 | 1.07911 |
| 2015-07-01 | 1.15879 |
| 2015-08-01 | 1.16716 |
| 2015-09-01 | 0.962171 |
| 2015-10-01 | 1.08403 |
| 2015-11-01 | 1.17159 |
| 2015-12-01 | 1.10905 |
| 2016-01-01 | 0.825609 |
| 2016-02-01 | 1.00816 |
| 2016-03-01 | 1.07039 |
| 2016-04-01 | 1.04171 |
| 2016-05-01 | 1.02931 |
| 2016-06-01 | 1.11199 |
| 2016-07-01 | 0.995986 |
| 2016-08-01 | 1.04537 |
| 2016-09-01 | 0.884864 |
| 2016-10-01 | 1.02522 |
| 2016-11-01 | 1.08673 |
| 2016-12-01 | 1.02522 |
| 2017-01-01 | 0.742511 |
| 2017-02-01 | 1.02401 |
| 2017-03-01 | 1.15048 |
| 2017-04-01 | 0.975904 |
| 2017-05-01 | 1.06536 |
| 2017-06-01 | 0.91491 |
| 2017-07-01 | 0.838394 |
| 2017-08-01 | 1.05305 |
| 2017-09-01 | 0.9518 |
| 2017-10-01 | 0.965305 |
| 2017-11-01 | 1.09455 |
| 2017-12-01 | 0.904721 |
| 2018-01-01 | 0.69127 |
| 2018-02-01 | 0.848011 |
| 2018-03-01 | 1.04092 |
| 2018-04-01 | 0.781148 |
| 2018-05-01 | 0.957407 |
| 2018-06-01 | 0.705036 |
| 2018-07-01 | 0.861677 |
| 2018-08-01 | 0.985344 |
| 2018-09-01 | 0.905559 |
| 2018-10-01 | 0.998165 |
| 2018-11-01 | 0.986235 |
| 2018-12-01 | 0.779444 |
| 2019-01-01 | 0.641205 |
| 2019-02-01 | 0.97764 |
| 2019-03-01 | 0.890562 |
| 2019-04-01 | 0.710484 |
| 2019-05-01 | 0.907841 |
| 2019-06-01 | 0.852581 |
| 2019-07-01 | 0.786223 |
| 2019-08-01 | 0.770499 |
| 2019-09-01 | 0.84519 |
| 2019-10-01 | 0.877727 |
| 2019-11-01 | 0.916911 |
| 2019-12-01 | 0.846379 |
| 2020-01-01 | 0.694555 |
| 2020-02-01 | 0.842831 |
| 2020-03-01 | 0.846733 |
| 2020-04-01 | 0.34309 |
| 2020-05-01 | 0.705674 |
| 2020-06-01 | 0.853826 |
| 2020-07-01 | 0.869961 |
| 2020-08-01 | 0.745123 |
| 2020-09-01 | 0.784135 |
| 2020-10-01 | 0.858346 |
| 2020-11-01 | 0.792019 |
| 2020-12-01 | 0.659366 |
| 2021-01-01 | 0.553541 |
| 2021-02-01 | 0.760145 |
| 2021-03-01 | 0.861498 |
| 2021-04-01 | 0.720253 |
| 2021-05-01 | 0.949956 |
| 2021-06-01 | 0.770866 |
| 2021-07-01 | 0.665246 |
| 2021-08-01 | 0.661356 |
| 2021-09-01 | 0.70804 |
| 2021-10-01 | 0.713903 |
| 2021-11-01 | 0.830301 |
| 2021-12-01 | 0.620786 |
| 2022-01-01 | 0.45537 |
| 2022-02-01 | 0.563424 |
| 2022-03-01 | 0.740941 |
| 2022-04-01 | 0.69181 |
| 2022-05-01 | 0.69181 |
| 2022-06-01 | 0.707183 |
| 2022-07-01 | 0.676365 |
| 2022-08-01 | 0.852143 |
| 2022-09-01 | 0.764254 |
| 2022-10-01 | 0.664886 |
| 2022-11-01 | 0.664886 |
| 2022-12-01 | 0.710479 |
| 2023-01-01 | 0.660021 |
| 2023-02-01 | 0.63362 |
| 2023-03-01 | 0.690194 |
| 2023-04-01 | 0.577646 |
| 2023-05-01 | 0.79145 |
| 2023-06-01 | 0.723933 |
| 2023-07-01 | 0.696952 |
| 2023-08-01 | 0.816216 |
| 2023-09-01 | 0.693225 |
| 2023-10-01 | 0.638061 |
| 2023-11-01 | 0.845802 |
| 2023-12-01 | 0.667739 |
| 2024-01-01 | 0.571671 |
| 2024-02-01 | 0.626994 |
| 2024-03-01 | 0.730263 |
| 2024-04-01 | 0.625143 |
| 2024-05-01 | 0.790622 |
| 2024-06-01 | 0.558951 |
| 2024-07-01 | 0.739832 |
| 2024-08-01 | 0.879008 |
| 2024-09-01 | 0.791108 |
| 2024-10-01 | 0.799485 |
| 2024-11-01 | 0.752027 |
| 2024-12-01 | 0.689966 |
| 2025-01-01 | 0.620986 |
| 2025-02-01 | 0.70451 |
| 2025-03-01 | 0.722667 |
| 2025-04-01 | 0.63188 |
| 2025-05-01 | 0.769877 |
| 2025-06-01 | 0.6573 |
| 2025-07-01 | 0.780771 |
| 2025-08-01 | 0.675458 |

Ampicillin

|  | Ampicillin injection 1 g 03314K | Ampicillin injection 500 mg 03313J |
| --- | --- | --- |
| 2014-03-01 | 0.00427237 |  |
| 2014-04-01 | 0.00425973 |  |
| 2014-06-01 | 0.00425973 |  |
| 2014-12-01 | 0.00423006 |  |
| 2015-07-01 | 0.00418335 |  |
| 2015-09-01 | 0.00418335 |  |
| 2015-11-01 | 0.00833869 |  |
| 2016-01-01 | 0.00414879 |  |
| 2016-03-01 | 0.0124464 |  |
| 2016-12-01 | 0.00410087 |  |
| 2017-04-01 | 0.00813253 |  |
| 2017-05-01 | 0.00813253 |  |
| 2017-07-01 | 0.00810042 |  |
| 2017-08-01 | 0.00405021 | 0.00405021 |
| 2017-12-01 | 0.00807786 |  |
| 2018-01-01 | 0.00401901 |  |
| 2018-04-01 | 0.00400589 |  |
| 2018-09-01 | 0.00398925 |  |
| 2019-01-01 | 0.0118742 |  |
| 2019-02-01 | 0.00791611 |  |
| 2019-03-01 | 0.00395806 |  |
| 2019-05-01 | 0.00394714 |  |
| 2019-08-01 | 0.00786223 |  |
| 2019-09-01 | 0.00786223 |  |
| 2019-10-01 | 0.00783685 |  |
| 2019-12-01 | 0.0195921 |  |
| 2020-06-01 | 0.00389875 |  |
| 2020-07-01 | 0.00390117 |  |
| 2020-10-01 | 0.00780314 |  |

Aspirin

|  | Aspirin dispersible tablet 300 mg 05018D |
| --- | --- |
| 2015-04-01 | 0.00419886 |
| 2015-07-01 | 0.00418335 |
| 2016-02-01 | 0.00414879 |
| 2016-05-01 | 0.00413378 |
| 2016-07-01 | 0.00411564 |
| 2016-08-01 | 0.00411564 |
| 2016-11-01 | 0.00410087 |
| 2017-01-01 | 0.00407973 |
| 2017-04-01 | 0.00406627 |
| 2017-05-01 | 0.00406627 |
| 2017-06-01 | 0.00406627 |
| 2018-03-01 | 0.00401901 |
| 2018-12-01 | 0.00397675 |
| 2020-01-01 | 0.00390199 |

Atropine

|  | Atropine sulfate injection 600 micrograms in 1 mL 05022H |
| --- | --- |
| 2014-04-01 | 0.00425973 |
| 2014-05-01 | 0.00425973 |
| 2014-08-01 | 0.00424396 |
| 2015-06-01 | 0.00419886 |
| 2015-12-01 | 0.00416935 |
| 2016-07-01 | 0.00411564 |
| 2016-09-01 | 0.00411564 |
| 2016-11-01 | 0.00410087 |
| 2017-02-01 | 0.00407973 |
| 2017-03-01 | 0.00407973 |
| 2017-11-01 | 0.00403893 |
| 2018-05-01 | 0.00400589 |
| 2019-09-01 | 0.00393112 |
| 2021-10-01 | 0.00387991 |
| 2023-06-01 | 0.00375095 |

Benzathine benzylpenicillin

|  | Benzathine benzylpenicillin injection 1,200,000 units in 2.3 mL 05027N | Benzathin benzylpenicillin injection 600,000 units in 1.17 mL 11735P |
| --- | --- | --- |
| 2015-02-01 | 0.0042113 |  |
| 2016-10-01 | 0.00410087 |  |
| 2016-12-01 | 0.00410087 |  |
| 2018-01-01 | 0.00401901 |  |
| 2018-07-01 | 0.00398925 |  |
| 2019-03-01 | 0.00395806 |  |
| 2019-04-01 | 0.00789427 |  |
| 2019-05-01 | 0.00394714 |  |
| 2020-05-01 |  | 0.00389875 |

Benzydamine

|  | Benzydamine mouth and throat rinse 22.5 mg per 15 mL, 500 mL 05032W |
| --- | --- |
| 2014-02-01 | 0.00854475 |
| 2014-03-01 | 0.00854475 |
| 2014-05-01 | 0.00851945 |
| 2014-07-01 | 0.0127319 |
| 2014-08-01 | 0.00424396 |
| 2014-09-01 | 0.0212198 |
| 2014-10-01 | 0.0126902 |
| 2014-11-01 | 0.00846012 |
| 2014-12-01 | 0.0211503 |
| 2015-01-01 | 0.0084226 |
| 2015-02-01 | 0.0084226 |
| 2015-03-01 | 0.0042113 |
| 2015-04-01 | 0.0125966 |
| 2015-05-01 | 0.00839772 |
| 2015-07-01 | 0.00836671 |
| 2015-09-01 | 0.00836671 |
| 2015-12-01 | 0.00416935 |
| 2016-02-01 | 0.00414879 |
| 2016-03-01 | 0.00829758 |
| 2016-04-01 | 0.0124014 |
| 2016-05-01 | 0.0206689 |
| 2016-06-01 | 0.00413378 |
| 2016-08-01 | 0.00823129 |
| 2016-11-01 | 0.0246052 |
| 2016-12-01 | 0.00410087 |
| 2017-02-01 | 0.00407973 |
| 2017-03-01 | 0.0122392 |
| 2017-04-01 | 0.00406627 |
| 2017-05-01 | 0.00813253 |
| 2017-07-01 | 0.00405021 |
| 2017-08-01 | 0.00810042 |
| 2017-11-01 | 0.00403893 |
| 2017-12-01 | 0.00403893 |
| 2018-01-01 | 0.00803802 |
| 2018-02-01 | 0.00401901 |
| 2018-03-01 | 0.00803802 |
| 2018-06-01 | 0.00400589 |
| 2018-09-01 | 0.00398925 |
| 2018-10-01 | 0.00397675 |
| 2018-11-01 | 0.00795351 |
| 2018-12-01 | 0.00397675 |
| 2019-01-01 | 0.00395806 |
| 2019-09-01 | 0.00393112 |
| 2020-01-01 | 0.00390199 |
| 2020-02-01 | 0.00390199 |
| 2020-03-01 | 0.011706 |
| 2020-06-01 | 0.00389875 |
| 2020-07-01 | 0.00780234 |
| 2020-08-01 | 0.00780234 |
| 2020-09-01 | 0.00390117 |
| 2020-10-01 | 0.00390157 |
| 2020-12-01 | 0.00390157 |
| 2021-01-01 | 0.0155927 |
| 2021-03-01 | 0.00389818 |
| 2021-05-01 | 0.00389326 |
| 2021-06-01 | 0.00389326 |
| 2021-07-01 | 0.00778066 |
| 2021-08-01 | 0.00778066 |
| 2021-10-01 | 0.00387991 |
| 2021-11-01 | 0.00775982 |
| 2021-12-01 | 0.00775982 |
| 2022-02-01 | 0.00385907 |
| 2022-04-01 | 0.00384339 |
| 2022-05-01 | 0.00384339 |
| 2022-07-01 | 0.00764254 |
| 2022-08-01 | 0.00764254 |
| 2022-09-01 | 0.00382127 |
| 2022-10-01 | 0.00379935 |
| 2022-12-01 | 0.0075987 |
| 2023-03-01 | 0.00377155 |
| 2023-05-01 | 0.0075019 |
| 2023-07-01 | 0.00372701 |
| 2023-08-01 | 0.00372701 |
| 2023-09-01 | 0.00745403 |
| 2023-12-01 | 0.00370966 |
| 2024-02-01 | 0.0036882 |
| 2024-04-01 | 0.00367731 |
| 2024-05-01 | 0.00367731 |
| 2024-06-01 | 0.00367731 |
| 2024-08-01 | 0.00366253 |
| 2024-09-01 | 0.00732507 |
| 2024-11-01 | 0.00365062 |
| 2024-12-01 | 0.0146025 |
| 2025-02-01 | 0.00726299 |
| 2025-03-01 | 0.00363149 |

Benzylpenicillin

|  | Benzylpenicillin injection 3 g 03399X | Benzylpenicillin injection 600 mg 03398W |
| --- | --- | --- |
| 2014-05-01 |  | 0.00425973 |
| 2014-08-01 |  | 0.00424396 |
| 2014-10-01 |  | 0.00423006 |
| 2015-01-01 |  | 0.0126339 |
| 2015-03-01 |  | 0.0042113 |
| 2015-05-01 |  | 0.00419886 |
| 2015-06-01 |  | 0.00839772 |
| 2015-08-01 |  | 0.00836671 |
| 2015-11-01 |  | 0.00833869 |
| 2015-12-01 |  | 0.00416935 |
| 2016-01-01 |  | 0.00414879 |
| 2016-05-01 |  | 0.00826757 |
| 2016-09-01 |  | 0.0164626 |
| 2016-12-01 |  | 0.00410087 |
| 2017-01-01 |  | 0.0163189 |
| 2017-02-01 |  | 0.00815947 |
| 2017-03-01 |  | 0.00407973 |
| 2017-07-01 |  | 0.00405021 |
| 2017-08-01 |  | 0.0121506 |
| 2017-09-01 |  | 0.00405021 |
| 2017-11-01 |  | 0.00403893 |
| 2017-12-01 |  | 0.00807786 |
| 2018-01-01 |  | 0.00803802 |
| 2018-02-01 |  | 0.00401901 |
| 2018-11-01 |  | 0.00795351 |
| 2019-05-01 | 0.00394714 |  |
| 2019-08-01 |  | 0.00786223 |
| 2020-11-01 |  | 0.00780314 |
| 2021-06-01 |  | 0.00389326 |
| 2022-04-01 |  | 0.00384339 |
| 2022-10-01 |  | 0.00379935 |
| 2022-12-01 |  | 0.0075987 |
| 2023-02-01 |  | 0.00377155 |
| 2023-07-01 |  | 0.00372701 |
| 2023-08-01 |  | 0.00372701 |
| 2023-09-01 |  | 0.00372701 |
| 2023-10-01 |  | 0.00741932 |
| 2024-03-01 |  | 0.00737639 |
| 2024-04-01 |  | 0.00367731 |
| 2024-08-01 |  | 0.00366253 |
| 2025-05-01 |  | 0.00363149 |
| 2025-06-01 |  | 0.00363149 |

Betamethasone acetate with betamethasone sodium phosphate

|  | Betamethasone acetate with betametasone sodium phosphate injection 3 mg with 3.9 mg in 1 mL 05034Y |
| --- | --- |
| 2014-02-01 | 0.00427237 |
| 2014-03-01 | 0.00427237 |
| 2014-05-01 | 0.00851945 |
| 2014-06-01 | 0.00851945 |
| 2014-07-01 | 0.00848792 |
| 2014-08-01 | 0.00424396 |
| 2014-09-01 | 0.00848792 |
| 2014-10-01 | 0.00846012 |
| 2014-11-01 | 0.00846012 |
| 2014-12-01 | 0.00423006 |
| 2015-02-01 | 0.0042113 |
| 2015-03-01 | 0.0126339 |
| 2015-04-01 | 0.00419886 |
| 2015-05-01 | 0.00839772 |
| 2015-06-01 | 0.00419886 |
| 2015-07-01 | 0.00836671 |
| 2015-08-01 | 0.00418335 |
| 2015-09-01 | 0.00418335 |
| 2015-11-01 | 0.00833869 |
| 2016-01-01 | 0.00829758 |
| 2016-02-01 | 0.00414879 |
| 2016-03-01 | 0.0124464 |
| 2016-04-01 | 0.00413378 |
| 2016-05-01 | 0.00413378 |
| 2016-06-01 | 0.00826757 |
| 2016-07-01 | 0.00411564 |
| 2016-08-01 | 0.00823129 |
| 2016-10-01 | 0.00820174 |
| 2016-11-01 | 0.00820174 |
| 2017-01-01 | 0.00815947 |
| 2017-02-01 | 0.00407973 |
| 2017-06-01 | 0.00406627 |
| 2017-07-01 | 0.00405021 |
| 2017-08-01 | 0.00405021 |
| 2017-10-01 | 0.00403893 |
| 2017-12-01 | 0.00403893 |
| 2018-01-01 | 0.012057 |
| 2018-03-01 | 0.00401901 |
| 2018-04-01 | 0.00400589 |
| 2018-05-01 | 0.00801177 |
| 2018-07-01 | 0.00398925 |
| 2018-09-01 | 0.00398925 |
| 2018-10-01 | 0.00795351 |
| 2018-11-01 | 0.00397675 |
| 2018-12-01 | 0.00397675 |
| 2019-02-01 | 0.00395806 |
| 2019-03-01 | 0.00791611 |
| 2019-04-01 | 0.00394714 |
| 2019-07-01 | 0.00786223 |
| 2019-09-01 | 0.00786223 |
| 2019-10-01 | 0.00783685 |
| 2019-12-01 | 0.0156737 |
| 2020-01-01 | 0.00390199 |
| 2020-02-01 | 0.00390199 |
| 2020-03-01 | 0.00390199 |
| 2020-05-01 | 0.00389875 |
| 2020-06-01 | 0.00389875 |
| 2020-07-01 | 0.00780234 |
| 2020-08-01 | 0.00390117 |
| 2020-09-01 | 0.00390117 |
| 2020-10-01 | 0.0117047 |
| 2020-11-01 | 0.0156063 |
| 2020-12-01 | 0.00780314 |
| 2021-01-01 | 0.00389818 |
| 2021-02-01 | 0.00389818 |
| 2021-03-01 | 0.00389818 |
| 2021-06-01 | 0.00778652 |
| 2021-07-01 | 0.00389033 |
| 2021-10-01 | 0.00387991 |
| 2021-11-01 | 0.00387991 |
| 2022-04-01 | 0.00768677 |
| 2022-06-01 | 0.00768677 |
| 2022-07-01 | 0.00382127 |
| 2022-08-01 | 0.00382127 |
| 2022-09-01 | 0.00764254 |
| 2023-03-01 | 0.00377155 |
| 2023-04-01 | 0.00375095 |
| 2023-05-01 | 0.0075019 |
| 2023-06-01 | 0.00375095 |
| 2023-08-01 | 0.00372701 |
| 2023-09-01 | 0.00745403 |
| 2023-10-01 | 0.00370966 |
| 2024-02-01 | 0.0036882 |
| 2024-05-01 | 0.00367731 |
| 2024-06-01 | 0.00367731 |
| 2024-12-01 | 0.00365062 |
| 2025-01-01 | 0.00726299 |
| 2025-02-01 | 0.00363149 |
| 2025-03-01 | 0.00363149 |

Carbamazepine

|  | Carbamazepine Tablet 200 mg 01724R | Carbamazepine Tablet 100mg 01755J | Carbamazepine Tablet 400 mg (controlled release) 05037D | Carbamazepine Tablet 200 mg (controlled release) 05038E | Carbamazepine Tablet 100 mg 05039F | Carbamazepine Oral suspension 100 mg per 5 mL, 300 mL 05041H | Carbamazepine Tablet 200 mg (controlled release) 14671E | Carbamazepine Tablet 400 mg (controlled release) 14680P |
| --- | --- | --- | --- | --- | --- | --- | --- | --- |
| 2014-02-01 | 0.0170895 | 0.00854475 |  | 0.0170895 | 0.0469961 |  |  |  |
| 2014-03-01 | 0.0213619 | 0.00854475 |  | 0.00854475 | 0.0384514 |  |  |  |
| 2014-04-01 | 0.00854475 | 0.0128171 |  | 0.0128171 | 0.0427237 |  |  |  |
| 2014-05-01 | 0.0170895 | 0.00427237 |  | 0.0256342 | 0.0384514 |  |  |  |
| 2014-06-01 | 0.0213619 | 0.00854475 |  | 0.00427237 | 0.0384514 |  |  |  |
| 2014-07-01 | 0.0128171 |  |  | 0.0170895 | 0.0384514 |  |  |  |
| 2014-08-01 | 0.0299066 |  |  | 0.0299066 | 0.0213619 |  |  |  |
| 2014-09-01 | 0.00427237 |  |  | 0.00427237 | 0.0384514 | 0.00854475 |  |  |
| 2014-10-01 | 0.0256342 |  |  | 0.0213619 | 0.0427237 | 0.00427237 |  |  |
| 2014-11-01 | 0.0170895 |  |  | 0.0128171 | 0.0512685 | 0.00427237 |  |  |
| 2014-12-01 | 0.0170895 |  |  | 0.0128171 | 0.034179 | 0.00427237 |  |  |
| 2015-01-01 | 0.0213619 |  |  | 0.0128171 | 0.0299066 |  |  |  |
| 2015-02-01 | 0.0213619 |  |  | 0.0170895 | 0.0469961 | 0.00427237 |  |  |
| 2015-03-01 | 0.0170895 |  |  | 0.00854475 | 0.0256342 |  |  |  |
| 2015-04-01 | 0.0128171 |  |  | 0.00854475 | 0.0299066 |  |  |  |
| 2015-05-01 |  |  | 0.00427237 | 0.0170895 | 0.0213619 | 0.00427237 |  |  |
| 2015-06-01 | 0.0170895 |  |  | 0.0213619 | 0.0427237 |  |  |  |
| 2015-07-01 | 0.00854475 |  |  | 0.0128171 | 0.0469961 |  |  |  |
| 2015-08-01 | 0.00854475 |  |  | 0.0213619 | 0.0299066 |  |  |  |
| 2015-09-01 |  |  |  | 0.0170895 | 0.0256342 |  |  |  |
| 2015-10-01 | 0.0128171 |  |  | 0.0128171 | 0.0427237 |  |  |  |
| 2015-11-01 | 0.00854475 |  |  | 0.0256342 | 0.0170895 | 0.00427237 |  |  |
| 2015-12-01 | 0.00427237 |  |  | 0.0170895 | 0.0384514 | 0.00427237 |  |  |
| 2016-01-01 | 0.00854475 |  |  | 0.0170895 | 0.0384514 |  |  |  |
| 2016-02-01 |  |  |  | 0.00854475 | 0.0299066 |  |  |  |
| 2016-03-01 | 0.0256342 |  |  |  | 0.0170895 |  |  |  |
| 2016-04-01 | 0.0170895 |  |  | 0.00427237 | 0.0256342 |  |  |  |
| 2016-05-01 | 0.0170895 |  |  | 0.00427237 | 0.0299066 | 0.00427237 |  |  |
| 2016-06-01 | 0.0128171 |  |  | 0.0213619 | 0.0469961 |  |  |  |
| 2016-07-01 |  |  |  | 0.00854475 | 0.0555409 |  |  |  |
| 2016-08-01 | 0.0170895 |  |  |  | 0.0384514 |  |  |  |
| 2016-09-01 | 0.00854475 |  |  | 0.0128171 | 0.0427237 |  |  |  |
| 2016-10-01 | 0.00854475 |  |  | 0.00854475 | 0.0427237 |  |  |  |
| 2016-11-01 | 0.00854475 |  |  | 0.00427237 | 0.0555409 |  |  |  |
| 2016-12-01 | 0.0256342 |  |  | 0.0170895 | 0.0384514 |  |  |  |
| 2017-01-01 | 0.0256342 |  |  | 0.00854475 | 0.0256342 |  |  |  |
| 2017-02-01 | 0.00854475 |  |  | 0.0128171 | 0.0384514 |  |  |  |
| 2017-03-01 | 0.0256342 |  | 0.00427237 | 0.0128171 | 0.0299066 |  |  |  |
| 2017-04-01 | 0.0128171 |  |  | 0.0299066 | 0.0555409 |  |  |  |
| 2017-05-01 | 0.0128171 |  |  | 0.00427237 | 0.0598132 |  |  |  |
| 2017-06-01 | 0.0128171 |  |  | 0.0128171 | 0.0299066 |  |  |  |
| 2017-07-01 | 0.00427237 |  | 0.00427237 |  | 0.034179 |  |  |  |
| 2017-08-01 | 0.0128171 |  |  | 0.00427237 | 0.034179 |  |  |  |
| 2017-09-01 | 0.0213619 |  |  | 0.00427237 | 0.0299066 |  |  |  |
| 2017-10-01 | 0.00854475 |  |  |  | 0.0512685 |  |  |  |
| 2017-11-01 | 0.00854475 |  |  | 0.00854475 | 0.0384514 |  |  |  |
| 2017-12-01 | 0.0213619 |  | 0.00427237 |  | 0.0213619 |  |  |  |
| 2018-01-01 | 0.0213619 |  | 0.00427237 | 0.00854475 | 0.0213619 |  |  |  |
| 2018-02-01 | 0.00854475 |  |  | 0.00854475 | 0.0427237 |  |  |  |
| 2018-03-01 | 0.0170895 |  |  |  | 0.0213619 |  |  |  |
| 2018-04-01 | 0.00854475 |  |  | 0.00427237 | 0.0299066 |  |  |  |
| 2018-05-01 | 0.0170895 |  |  | 0.00854475 | 0.0256342 |  |  |  |
| 2018-06-01 | 0.0213619 |  |  | 0.00427237 | 0.0170895 |  |  |  |
| 2018-07-01 | 0.00854475 |  |  |  | 0.034179 |  |  |  |
| 2018-08-01 | 0.0256342 |  |  | 0.00427237 | 0.0213619 | 0.00854475 |  |  |
| 2018-09-01 | 0.00427237 |  | 0.00427237 | 0.0128171 | 0.034179 |  |  |  |
| 2018-10-01 | 0.034179 |  |  | 0.0213619 | 0.0512685 | 0.00427237 |  |  |
| 2018-11-01 | 0.0213619 |  |  | 0.0213619 | 0.0427237 |  |  |  |
| 2018-12-01 | 0.0128171 |  | 0.00427237 | 0.00427237 | 0.0427237 |  |  |  |
| 2019-01-01 | 0.0170895 |  |  | 0.00854475 | 0.0384514 | 0.00427237 |  |  |
| 2019-02-01 | 0.0170895 |  | 0.00854475 | 0.00854475 | 0.0299066 |  |  |  |
| 2019-03-01 | 0.0128171 |  |  | 0.0170895 | 0.00854475 |  |  |  |
| 2019-04-01 | 0.0128171 |  |  | 0.00427237 | 0.0299066 |  |  |  |
| 2019-05-01 | 0.00427237 |  |  | 0.0170895 | 0.0469961 |  |  |  |
| 2019-06-01 | 0.0170895 |  |  | 0.00854475 | 0.0384514 |  |  |  |
| 2019-07-01 | 0.0128171 |  |  | 0.0170895 | 0.0427237 | 0.00427237 |  |  |
| 2019-08-01 | 0.00854475 |  |  | 0.0128171 | 0.00427237 |  |  |  |
| 2019-09-01 | 0.0170895 |  |  | 0.00854475 | 0.0469961 |  |  |  |
| 2019-10-01 | 0.0170895 |  |  | 0.00427237 | 0.0256342 |  |  |  |
| 2019-11-01 | 0.0128171 |  | 0.00427237 | 0.00854475 | 0.0854475 |  |  |  |
| 2019-12-01 | 0.00854475 |  |  | 0.00427237 | 0.0170895 |  |  |  |
| 2020-01-01 | 0.00854475 |  | 0.00427237 | 0.00854475 | 0.0256342 |  |  |  |
| 2020-02-01 | 0.0170895 |  | 0.00427237 |  | 0.0299066 |  |  |  |
| 2020-03-01 | 0.0128171 |  |  | 0.00427237 | 0.034179 | 0.00427237 |  |  |
| 2020-04-01 | 0.00854475 |  |  | 0.00427237 | 0.0512685 | 0.00427237 |  |  |
| 2020-05-01 | 0.0170895 |  |  |  | 0.0299066 |  |  |  |
| 2020-06-01 | 0.00854475 |  |  | 0.00854475 | 0.034179 |  |  |  |
| 2020-07-01 | 0.00854475 |  |  |  | 0.0469961 | 0.00427237 |  |  |
| 2020-08-01 | 0.00854475 |  |  | 0.00854475 | 0.0213619 |  |  |  |
| 2020-09-01 | 0.0128171 |  |  | 0.00427237 | 0.0256342 |  |  |  |
| 2020-10-01 | 0.00854475 |  |  | 0.00854475 | 0.034179 |  |  |  |
| 2020-11-01 | 0.0170895 |  |  | 0.00427237 | 0.0299066 |  |  |  |
| 2020-12-01 | 0.00854475 |  |  | 0.00427237 | 0.0256342 |  |  |  |
| 2021-01-01 | 0.0128171 |  |  |  | 0.0299066 |  |  |  |
| 2021-02-01 | 0.0213619 |  | 0.00427237 |  | 0.0299066 | 0.00427237 |  |  |
| 2021-03-01 | 0.0128171 |  |  | 0.00854475 | 0.034179 |  |  |  |
| 2021-04-01 | 0.0128171 |  |  | 0.00427237 | 0.0427237 |  |  |  |
| 2021-05-01 | 0.0128171 |  |  | 0.0170895 | 0.0256342 |  |  |  |
| 2021-06-01 | 0.0213619 |  | 0.00427237 |  | 0.0512685 |  |  |  |
| 2021-07-01 | 0.00427237 |  |  |  | 0.0427237 |  |  |  |
| 2021-08-01 | 0.0170895 |  | 0.00427237 | 0.00427237 | 0.0299066 |  |  |  |
| 2021-09-01 | 0.0256342 |  |  |  | 0.0512685 |  |  |  |
| 2021-10-01 | 0.0256342 |  |  |  | 0.0555409 |  |  |  |
| 2021-11-01 | 0.00854475 |  | 0.00427237 |  | 0.034179 |  |  |  |
| 2021-12-01 | 0.0170895 |  |  | 0.00427237 | 0.0726304 |  |  |  |
| 2022-01-01 | 0.00427237 |  | 0.00427237 | 0.00427237 | 0.0299066 |  |  |  |
| 2022-02-01 | 0.00854475 |  |  | 0.0128171 | 0.0384514 | 0.00427237 |  |  |
| 2022-03-01 | 0.0170895 |  | 0.00427237 | 0.00427237 | 0.034179 |  |  |  |
| 2022-04-01 | 0.0128171 |  |  |  | 0.0213619 | 0.00427237 |  |  |
| 2022-05-01 | 0.0170895 |  |  |  | 0.0256342 |  |  |  |
| 2022-06-01 | 0.0170895 |  |  | 0.00427237 | 0.0128171 |  |  |  |
| 2022-07-01 | 0.00854475 |  | 0.00427237 | 0.00854475 | 0.0299066 |  |  |  |
| 2022-08-01 | 0.00854475 |  | 0.0170895 |  | 0.0555409 |  |  |  |
| 2022-09-01 | 0.00427237 |  | 0.00854475 | 0.00854475 | 0.0427237 |  |  |  |
| 2022-10-01 |  |  |  | 0.00427237 | 0.0170895 |  |  |  |
| 2022-11-01 |  |  |  | 0.00427237 | 0.0299066 | 0.00427237 |  |  |
| 2022-12-01 | 0.0170895 |  |  | 0.00854475 | 0.0469961 |  |  |  |
| 2023-01-01 | 0.00427237 |  |  | 0.00854475 | 0.0384514 |  |  |  |
| 2023-02-01 | 0.00854475 |  |  | 0.0128171 | 0.034179 |  |  |  |
| 2023-03-01 | 0.00427237 |  |  | 0.0170895 | 0.0256342 |  |  |  |
| 2023-04-01 | 0.0128171 |  |  | 0.0128171 | 0.0170895 |  |  |  |
| 2023-05-01 | 0.00427237 |  |  |  | 0.0213619 |  |  |  |
| 2023-06-01 | 0.0128171 |  |  | 0.0128171 | 0.0299066 | 0.00427237 |  |  |
| 2023-07-01 | 0.00854475 |  | 0.00427237 | 0.00854475 | 0.034179 |  |  |  |
| 2023-08-01 | 0.0170895 |  |  | 0.00427237 | 0.0598132 | 0.00427237 |  |  |
| 2023-09-01 | 0.00427237 |  |  | 0.0128171 | 0.0427237 |  |  |  |
| 2023-10-01 | 0.00427237 |  |  |  | 0.034179 | 0.00427237 |  |  |
| 2023-11-01 | 0.00854475 |  | 0.00854475 | 0.0170895 | 0.0128171 |  |  |  |
| 2023-12-01 | 0.0213619 |  |  | 0.00854475 | 0.0128171 |  |  |  |
| 2024-01-01 | 0.00427237 |  |  |  | 0.00854475 |  |  |  |
| 2024-02-01 | 0 |  |  |  | 0.0128171 |  |  |  |
| 2024-03-01 | 0.00854475 |  |  |  | 0.0384514 |  |  |  |
| 2024-04-01 | 0.0128171 |  |  |  | 0.0256342 |  |  |  |
| 2024-05-01 | 0.00427237 |  |  | 0.00427237 | 0.034179 |  |  |  |
| 2024-06-01 | 0.0213619 |  |  | 0.0128171 | 0.0213619 |  |  |  |
| 2024-07-01 | 0.00854475 |  |  |  | 0.0299066 |  |  |  |
| 2024-08-01 | 0.0170895 |  |  |  | 0.0299066 |  |  |  |
| 2024-09-01 | 0.0128171 |  |  | 0.00427237 | 0.0128171 |  |  |  |
| 2024-10-01 | 0.0170895 |  |  | 0.00854475 | 0.0213619 |  |  |  |
| 2024-11-01 | 0.0170895 |  |  |  | 0.0299066 |  |  |  |
| 2024-12-01 | 0 |  |  | 0.00427237 | 0.0128171 |  |  |  |
| 2025-01-01 | 0.00854475 |  | 0.00427237 |  | 0.0170895 |  |  |  |
| 2025-02-01 | 0.0170895 |  |  | 0.00427237 | 0.0299066 |  | 0.00427237 |  |
| 2025-03-01 | 0.00854475 |  |  |  | 0.0384514 | 0.00427237 | 0.00427237 |  |
| 2025-04-01 | 0.0170895 |  |  | 0.00427237 | 0.0256342 |  | 0.00854475 | 0.00427237 |
| 2025-05-01 | 0 |  |  |  | 0.0170895 | 0.00427237 | 0.00427237 |  |
| 2025-06-01 | 0.0170895 |  | 0.00427237 |  | 0.0170895 |  | 0.00854475 |  |
| 2025-07-01 | 0.00854475 |  |  |  | 0.0299066 |  | 0.00427237 |  |
| 2025-08-01 | 0.00427237 |  |  |  | 0.0299066 |  | 0.00427237 |  |

Cefaclor

|  | Cefaclor suspension 125 mg per 5 mL, 100 mL 05046N | Cefaclor suspension 250 mg per 5 mL, 75 mL 05047P | Cefaclor tablet (sustained release) 375 mg 05045M |
| --- | --- | --- | --- |
| 2014-02-01 | 0.0384514 | 0.0256342 | 0.290521 |
| 2014-03-01 | 0.0256342 | 0.0427237 | 0.273432 |
| 2014-04-01 | 0.0212986 | 0.0212986 | 0.31096 |
| 2014-05-01 | 0.0212986 | 0.0170389 | 0.298181 |
| 2014-06-01 | 0.00851945 | 0.0425973 | 0.272622 |
| 2014-07-01 | 0.00848792 | 0.0466836 | 0.271613 |
| 2014-08-01 | 0.0254638 | 0.0339517 | 0.258882 |
| 2014-09-01 | 0.00848792 | 0.0381956 | 0.254638 |
| 2014-10-01 | 0.00423006 | 0.0338405 | 0.270724 |
| 2014-11-01 | 0.0253804 | 0.0338405 | 0.135362 |
| 2014-12-01 | 0.00846012 | 0.0380705 | 0.346865 |
| 2015-01-01 | 0.0126339 | 0.0210565 | 0.307425 |
| 2015-02-01 | 0.0126339 | 0.0336904 | 0.256889 |
| 2015-03-01 |  | 0.0210565 | 0.265312 |
| 2015-04-01 | 0.00839772 | 0.0167954 | 0.25613 |
| 2015-05-01 |  | 0.0209943 | 0.205744 |
| 2015-06-01 | 0.00839772 | 0.0125966 | 0.247733 |
| 2015-07-01 | 0.00836671 | 0.0292835 | 0.292835 |
| 2015-08-01 | 0.0125501 | 0.0418335 | 0.259368 |
| 2015-09-01 | 0.0209168 | 0.0251001 | 0.150601 |
| 2015-10-01 | 0.0208467 | 0.00833869 | 0.233483 |
| 2015-11-01 | 0.0208467 | 0.0333548 | 0.229314 |
| 2015-12-01 | 0.00416935 | 0.0375241 | 0.279346 |
| 2016-01-01 | 0.0124464 | 0.0207439 | 0.211588 |
| 2016-02-01 | 0.00829758 | 0.0207439 | 0.178398 |
| 2016-03-01 | 0.00414879 | 0.0331903 | 0.161803 |
| 2016-04-01 | 0.00826757 | 0.0165351 | 0.223224 |
| 2016-05-01 | 0.0165351 | 0.0330703 | 0.248027 |
| 2016-06-01 | 0.00826757 | 0.0124014 | 0.190154 |
| 2016-07-01 | 0.00411564 | 0.0246939 | 0.251054 |
| 2016-08-01 | 0.0123469 | 0.0329252 | 0.267517 |
| 2016-09-01 | 0.0246939 | 0.0288095 | 0.164626 |
| 2016-10-01 | 0.00410087 | 0.032807 | 0.168136 |
| 2016-11-01 |  | 0.0246052 | 0.168136 |
| 2016-12-01 | 0.00410087 | 0.00410087 | 0.176337 |
| 2017-01-01 |  | 0.0244784 | 0.118312 |
| 2017-02-01 | 0.00815947 | 0.00407973 | 0.15095 |
| 2017-03-01 | 0.0122392 | 0.00815947 | 0.179508 |
| 2017-04-01 | 0.00813253 | 0.0284639 | 0.117922 |
| 2017-05-01 | 0.00406627 | 0.00813253 | 0.178916 |
| 2017-06-01 | 0.00406627 | 0.0121988 | 0.211446 |
| 2017-07-01 | 0.0162008 | 0.0243013 | 0.18226 |
| 2017-08-01 | 0.0162008 | 0.0121506 | 0.263264 |
| 2017-09-01 | 0.00810042 | 0.0202511 | 0.157958 |
| 2017-10-01 | 0.00403893 | 0.0121168 | 0.165596 |
| 2017-11-01 |  | 0.0242336 | 0.185791 |
| 2017-12-01 | 0.00403893 |  | 0.181752 |
| 2018-01-01 | 0.00401901 | 0.0241141 | 0.213008 |
| 2018-02-01 | 0.00803802 | 0.012057 | 0.172817 |
| 2018-03-01 |  | 0.0281331 | 0.164779 |
| 2018-04-01 | 0.00801177 | 0.0280412 | 0.196288 |
| 2018-05-01 | 0.00400589 | 0.0280412 | 0.216318 |
| 2018-06-01 | 0.00400589 | 0.0240353 | 0.15623 |
| 2018-07-01 |  | 0.00797849 | 0.15957 |
| 2018-08-01 |  | 0.00398925 | 0.135634 |
| 2018-09-01 |  | 0.015957 | 0.0718064 |
| 2018-10-01 | 0.00397675 | 0.0198838 | 0.163047 |
| 2018-11-01 | 0.00397675 | 0.015907 | 0.163047 |
| 2018-12-01 | 0.00795351 | 0.00795351 | 0.139186 |
| 2019-01-01 |  | 0.00791611 | 0.138532 |
| 2019-02-01 | 0.00791611 | 0.0197903 | 0.186029 |
| 2019-03-01 | 0.00395806 | 0.0158322 | 0.193945 |
| 2019-04-01 | 0.00394714 | 0.0118414 | 0.122361 |
| 2019-05-01 |  | 0.0157885 | 0.146044 |
| 2019-06-01 | 0.00394714 | 0.00394714 | 0.217092 |
| 2019-07-01 |  | 0.00393112 | 0.172969 |
| 2019-08-01 | 0.0117933 | 0.0157245 | 0.157245 |
| 2019-09-01 | 0.00393112 | 0.0117933 | 0.161176 |
| 2019-10-01 | 0.00783685 | 0.00783685 | 0.1489 |
| 2019-11-01 | 0.00391842 | 0.0117553 | 0.188084 |
| 2019-12-01 |  | 0.0156737 | 0.1489 |
| 2020-01-01 | 0.00780399 | 0.00780399 | 0.148276 |
| 2020-02-01 |  | 0.023412 | 0.105354 |
| 2020-03-01 |  | 0.011706 | 0.140472 |
| 2020-04-01 | 0.0077975 | 0.0116962 | 0.0701775 |
| 2020-05-01 |  | 0.0077975 | 0.12476 |
| 2020-06-01 |  | 0.0233925 | 0.152051 |
| 2020-07-01 | 0.00390117 | 0.0117035 | 0.179454 |
| 2020-08-01 |  | 0.0117035 | 0.0897269 |
| 2020-09-01 |  | 0.00780234 | 0.136541 |
| 2020-10-01 |  | 0.0195079 | 0.144358 |
| 2020-11-01 | 0.00390157 | 0.0312126 | 0.175571 |
| 2020-12-01 |  | 0.0234094 | 0.140457 |
| 2021-01-01 | 0.0116945 | 0.0116945 | 0.116945 |
| 2021-02-01 |  | 0.0194909 | 0.136436 |
| 2021-03-01 |  | 0.0116945 | 0.105251 |
| 2021-04-01 | 0.00389326 | 0.0116798 | 0.0817585 |
| 2021-05-01 | 0.00778652 | 0.0311461 | 0.144051 |
| 2021-06-01 | 0.0116798 | 0.015573 | 0.105118 |
| 2021-07-01 | 0.00778066 | 0.0311226 | 0.11282 |
| 2021-08-01 |  | 0.00389033 | 0.128381 |
| 2021-09-01 | 0.00389033 | 0.0311226 | 0.11671 |
| 2021-10-01 |  | 0.0193995 | 0.108637 |
| 2021-11-01 |  | 0.0232795 | 0.0814781 |
| 2021-12-01 |  | 0.0193995 | 0.108637 |
| 2022-01-01 |  | 0.0154363 | 0.0617451 |
| 2022-02-01 |  | 0.0115772 | 0.0578861 |
| 2022-03-01 |  | 0.00385907 | 0.0733223 |
| 2022-04-01 |  | 0.00768677 | 0.0384339 |
| 2022-05-01 | 0.00384339 | 0.0115302 | 0.0653376 |
| 2022-06-01 |  | 0.00768677 | 0.099928 |
| 2022-07-01 |  | 0.0114638 | 0.126102 |
| 2022-08-01 | 0.00382127 | 0.0114638 | 0.099353 |
| 2022-09-01 |  | 0.00382127 | 0.0878892 |
| 2022-10-01 | 0.00379935 | 0.0113981 | 0.0417929 |
| 2022-11-01 | 0.0113981 | 0.0189968 | 0.0683883 |
| 2022-12-01 | 0.0075987 | 0.0189968 | 0.0607896 |
| 2023-01-01 | 0.0188577 | 0.0264008 | 0.0452586 |
| 2023-02-01 |  | 0.0075431 | 0.0678879 |
| 2023-03-01 | 0.00377155 | 0.0113146 | 0.101832 |
| 2023-04-01 | 0.00375095 | 0.0150038 | 0.101276 |
| 2023-05-01 | 0.00375095 | 0.00375095 | 0.0600152 |
| 2023-06-01 | 0.0075019 |  | 0.071268 |
| 2023-07-01 |  | 0.00745403 | 0.0559052 |
| 2023-08-01 | 0.00372701 | 0.00745403 | 0.0484512 |
| 2023-09-01 | 0.00372701 | 0.00372701 | 0.0596322 |
| 2023-10-01 | 0.00370966 | 0.00741932 | 0.0445159 |
| 2023-11-01 | 0.00370966 |  | 0.0667739 |
| 2023-12-01 |  | 0.011129 | 0.0482256 |
| 2024-01-01 |  | 0.0036882 | 0.0295056 |
| 2024-02-01 |  | 0.0036882 | 0.0295056 |
| 2024-03-01 | 0.0036882 | 0.0036882 | 0.0626994 |
| 2024-04-01 |  | 0.0147092 | 0.0367731 |
| 2024-05-01 | 0.00367731 | 0.00735462 | 0.047805 |
| 2024-06-01 | 0.00367731 | 0.00367731 | 0.058837 |
| 2024-07-01 | 0.00366253 | 0.00366253 | 0.0586006 |
| 2024-08-01 | 0.00732507 | 0.00366253 | 0.0329628 |
| 2024-09-01 | 0.00366253 | 0.00366253 | 0.0293003 |
| 2024-10-01 | 0.00365062 |  | 0.0365062 |
| 2024-11-01 |  |  | 0.0547592 |
| 2024-12-01 | 0.00365062 |  | 0.0438074 |
| 2025-01-01 |  | 0.00726299 | 0.029052 |
| 2025-02-01 |  | 0.00726299 | 0.014526 |
| 2025-03-01 |  | 0.00726299 | 0.0472094 |
| 2025-04-01 |  | 0.00363149 | 0.021789 |
| 2025-05-01 |  |  | 0.0326834 |
| 2025-06-01 |  | 0.00363149 | 0.0472094 |
| 2025-07-01 | 0.00363149 | 0.00363149 | 0.0326834 |
| 2025-08-01 | 0.00363149 |  | 0.0399464 |

Cefalotin

|  | Cefalotin injection 1 g 03376Q |
| --- | --- |
| 2014-08-01 | 0.00424396 |
| 2014-12-01 | 0.00423006 |
| 2015-05-01 | 0.00419886 |
| 2015-09-01 | 0.00836671 |
| 2015-11-01 | 0.00416935 |
| 2016-09-01 | 0.00411564 |

Cefuroxime

|  | Cefuroxime suspension 125 mg per 5 mL, 100 mL 11191B | Cefuroxime suspension 125 mg per 5 mL, 70 mL (S19A) 13653N | Cefuroxime suspension 125 mg per 5 mL, 70 mL 02002J | Cefuroxime tablet 250 mg 05052X | Cefuroxime tablet 250 mg 11228Y |
| --- | --- | --- | --- | --- | --- |
| 2014-02-01 |  |  |  | 0.034179 |  |
| 2014-03-01 |  |  |  | 0.0128171 |  |
| 2014-04-01 |  |  |  | 0.0170389 |  |
| 2014-05-01 |  |  | 0.00425973 | 0.0127792 |  |
| 2014-06-01 |  |  |  | 0.0255584 |  |
| 2014-07-01 |  |  |  | 0.0594154 |  |
| 2014-08-01 |  |  |  | 0.0169758 |  |
| 2014-09-01 |  |  |  | 0.0254638 |  |
| 2014-10-01 |  |  |  | 0.0296104 |  |
| 2014-11-01 |  |  |  | 0.0126902 |  |
| 2014-12-01 |  |  |  | 0.0380705 |  |
| 2015-01-01 |  |  |  | 0.0252678 |  |
| 2015-02-01 |  |  |  | 0.0042113 |  |
| 2015-03-01 |  |  |  | 0.0252678 |  |
| 2015-04-01 |  |  |  | 0.0209943 |  |
| 2015-05-01 |  |  |  | 0.0167954 |  |
| 2015-06-01 |  |  |  | 0.0125966 |  |
| 2015-07-01 |  |  |  | 0.0167334 |  |
| 2015-08-01 |  |  |  | 0.0125501 |  |
| 2015-09-01 |  |  | 0.00836671 | 0.0334668 |  |
| 2015-10-01 |  |  |  | 0.0416935 |  |
| 2015-11-01 |  |  |  | 0.0208467 |  |
| 2015-12-01 |  |  |  | 0.0416935 |  |
| 2016-01-01 |  |  |  | 0.0290415 |  |
| 2016-02-01 |  |  |  | 0.0373391 |  |
| 2016-03-01 |  |  |  | 0.0248927 |  |
| 2016-04-01 |  |  |  | 0.0289365 |  |
| 2016-05-01 |  |  |  | 0.0165351 |  |
| 2016-06-01 |  |  |  | 0.0289365 |  |
| 2016-07-01 |  |  |  | 0.0288095 |  |
| 2016-08-01 |  |  |  | 0.00823129 |  |
| 2016-09-01 |  |  |  | 0.0246939 |  |
| 2016-10-01 |  |  |  | 0.032807 |  |
| 2016-11-01 |  |  |  | 0.0123026 |  |
| 2016-12-01 |  |  |  | 0.0410087 |  |
| 2017-01-01 |  |  |  | 0.0326379 |  |
| 2017-02-01 |  |  |  | 0.0203987 |  |
| 2017-03-01 |  |  |  | 0.0448771 |  |
| 2017-04-01 |  |  |  | 0.0284639 |  |
| 2017-05-01 |  |  |  | 0.0284639 |  |
| 2017-06-01 |  |  |  | 0.0203313 |  |
| 2017-07-01 |  |  | 0.00405021 | 0.0445523 |  |
| 2017-08-01 |  |  |  | 0.0445523 |  |
| 2017-09-01 |  |  |  | 0.0607532 |  |
| 2017-10-01 |  |  |  | 0.00807786 |  |
| 2017-11-01 |  |  |  | 0.0242336 |  |
| 2017-12-01 |  |  |  | 0.0242336 |  |
| 2018-01-01 |  |  |  | 0.0241141 |  |
| 2018-02-01 |  |  |  | 0.00401901 |  |
| 2018-03-01 |  |  |  |  | 0.00803802 |
| 2018-04-01 |  |  |  | 0.00400589 | 0.0120177 |
| 2018-05-01 |  |  |  |  | 0.0160235 |
| 2018-06-01 |  |  |  |  | 0.0280412 |
| 2018-07-01 |  |  |  | 0.00797849 | 0.0199462 |
| 2018-08-01 |  |  |  | 0.00398925 | 0.0119677 |
| 2018-09-01 |  |  |  | 0.00398925 | 0.00797849 |
| 2018-10-01 |  |  |  | 0.00397675 | 0.00397675 |
| 2018-11-01 |  |  |  | 0.00397675 | 0.0119303 |
| 2018-12-01 |  |  |  |  | 0.0198838 |
| 2019-01-01 |  |  |  |  | 0.0158322 |
| 2019-02-01 |  |  |  | 0.00395806 | 0.0197903 |
| 2019-03-01 |  |  |  | 0.00395806 | 0.0158322 |
| 2019-04-01 |  |  |  |  | 0.0157885 |
| 2019-05-01 |  |  |  | 0.00394714 | 0.0197357 |
| 2019-06-01 | 0.00394714 |  |  |  | 0.00394714 |
| 2019-07-01 |  |  |  |  | 0.0196556 |
| 2019-08-01 |  |  |  |  | 0.0117933 |
| 2019-09-01 |  |  |  |  | 0.0157245 |
| 2019-10-01 |  |  |  |  | 0.00783685 |
| 2019-11-01 | 0.00783685 |  |  |  | 0.0235105 |
| 2019-12-01 | 0.00391842 |  |  | 0.00783685 | 0.0195921 |
| 2020-01-01 |  |  |  |  | 0.015608 |
| 2020-02-01 |  |  |  |  | 0.01951 |
| 2020-03-01 |  |  |  |  | 0.0351179 |
| 2020-04-01 |  |  |  | 0.00389875 | 0.0077975 |
| 2020-05-01 |  |  |  |  | 0.0077975 |
| 2020-07-01 |  |  |  |  | 0.0117035 |
| 2020-08-01 |  |  |  | 0.00390117 | 0.0195058 |
| 2020-09-01 |  |  |  | 0.00390117 | 0.00780234 |
| 2020-10-01 |  |  |  |  | 0.027311 |
| 2020-11-01 |  |  |  |  | 0.0195079 |
| 2020-12-01 |  |  |  |  | 0.0156063 |
| 2021-01-01 |  |  |  |  | 0.0116945 |
| 2021-02-01 |  |  |  |  | 0.0116945 |
| 2021-03-01 |  |  |  | 0.00389818 | 0.0194909 |
| 2021-04-01 |  |  |  | 0.00389326 | 0.0194663 |
| 2021-05-01 |  |  |  |  | 0.0856517 |
| 2021-06-01 | 0.00778652 |  |  |  | 0.0973315 |
| 2021-07-01 | 0.00778066 |  |  |  | 0.0700259 |
| 2021-08-01 |  |  |  |  | 0.023342 |
| 2021-09-01 |  |  |  |  | 0.00389033 |
| 2021-10-01 |  |  |  |  | 0.00775982 |
| 2021-11-01 |  |  |  |  | 0.0193995 |
| 2021-12-01 |  |  |  |  | 0.0116397 |
| 2022-01-01 |  |  |  |  | 0.0115772 |
| 2022-02-01 |  |  |  | 0.00771814 | 0.0154363 |
| 2022-03-01 |  |  |  |  | 0.0154363 |
| 2022-04-01 | 0.00384339 |  |  |  | 0.0153735 |
| 2022-05-01 |  |  |  |  | 0.0192169 |
| 2022-06-01 | 0.00384339 |  |  |  | 0.0192169 |
| 2022-07-01 |  |  |  |  | 0.0191063 |
| 2022-08-01 |  |  |  |  | 0.0114638 |
| 2022-09-01 |  |  |  |  | 0.0191063 |
| 2022-10-01 |  |  |  | 0.00379935 | 0.0303948 |
| 2022-11-01 |  |  |  |  | 0.0227961 |
| 2022-12-01 |  |  |  | 0.00379935 | 0.0113981 |
| 2023-01-01 |  |  |  |  | 0.0075431 |
| 2023-02-01 |  |  |  |  | 0.00377155 |
| 2023-03-01 |  |  |  |  | 0.00377155 |
| 2023-04-01 |  |  |  |  | 0.0337585 |
| 2023-05-01 |  |  |  |  | 0.0112528 |
| 2023-06-01 |  |  |  | 0.00375095 | 0.0075019 |
| 2023-07-01 |  |  |  |  | 0.0260891 |
| 2023-08-01 |  |  |  |  | 0.0186351 |
| 2023-09-01 |  |  |  | 0.00372701 | 0.0186351 |
| 2023-10-01 |  |  |  | 0.00370966 | 0.0185483 |
| 2023-11-01 |  |  |  | 0.00370966 | 0.0148386 |
| 2023-12-01 |  |  |  |  | 0.011129 |
| 2024-01-01 |  |  |  | 0.0036882 | 0.0221292 |
| 2024-02-01 |  |  |  |  | 0.018441 |
| 2024-03-01 |  |  |  | 0.00737639 | 0.0110646 |
| 2024-04-01 |  |  |  |  | 0.0294185 |
| 2024-05-01 |  |  |  |  | 0.00735462 |
| 2024-06-01 |  |  |  |  | 0.0110319 |
| 2024-07-01 |  |  |  |  | 0.0219752 |
| 2024-08-01 |  |  |  | 0.0109876 | 0.00366253 |
| 2024-09-01 |  |  |  | 0.00732507 | 0.0146501 |
| 2024-10-01 |  |  |  |  | 0.0109518 |
| 2024-11-01 |  |  |  |  | 0.0219037 |
| 2024-12-01 |  |  |  |  | 0.0146025 |
| 2025-01-01 |  |  |  |  | 0.00363149 |
| 2025-02-01 |  |  |  |  | 0.014526 |
| 2025-03-01 |  |  |  |  | 0.0181575 |
| 2025-04-01 |  |  |  |  | 0.0254205 |
| 2025-05-01 |  |  |  |  | 0.00363149 |
| 2025-06-01 |  |  |  |  | 0.0108945 |
| 2025-07-01 |  | 0.00363149 |  | 0.00726299 | 0.00726299 |
| 2025-08-01 |  |  |  | 0.00363149 | 0.014526 |

Chloramphenicol

|  | Chloramphenicol eye drops 5 mg per mL, 10 mL 05055C |
| --- | --- |
| 2014-02-01 | 0.0555409 |
| 2014-03-01 | 0.0213619 |
| 2014-04-01 | 0.0212986 |
| 2014-05-01 | 0.0425973 |
| 2014-06-01 | 0.046857 |
| 2014-07-01 | 0.0381956 |
| 2014-08-01 | 0.0297077 |
| 2014-09-01 | 0.0169758 |
| 2014-10-01 | 0.0507607 |
| 2014-11-01 | 0.0211503 |
| 2014-12-01 | 0.0211503 |
| 2015-01-01 | 0.0379017 |
| 2015-02-01 | 0.0252678 |
| 2015-03-01 | 0.0126339 |
| 2015-04-01 | 0.0125966 |
| 2015-05-01 | 0.0629829 |
| 2015-06-01 | 0.0125966 |
| 2015-07-01 | 0.0334668 |
| 2015-08-01 | 0.0376502 |
| 2015-09-01 | 0.0334668 |
| 2015-10-01 | 0.0208467 |
| 2015-11-01 | 0.0166774 |
| 2015-12-01 | 0.0333548 |

Codeine

|  | Codeine tablet 30 mg 05063L | Codeine tablet 30 mg 12054K |
| --- | --- | --- |
| 2014-02-01 | 0.0256342 |  |
| 2014-03-01 | 0.0427237 |  |
| 2014-04-01 | 0.0596362 |  |
| 2014-05-01 | 0.0724153 |  |
| 2014-06-01 | 0.0425973 |  |
| 2014-07-01 | 0.0339517 |  |
| 2014-08-01 | 0.0594154 |  |
| 2014-09-01 | 0.0509275 |  |
| 2014-10-01 | 0.0676809 |  |
| 2014-11-01 | 0.0296104 |  |
| 2014-12-01 | 0.0169202 |  |
| 2015-01-01 | 0.0252678 |  |
| 2015-02-01 | 0.0336904 |  |
| 2015-03-01 | 0.0294791 |  |
| 2015-04-01 | 0.117568 |  |
| 2015-05-01 | 0.058784 |  |
| 2015-06-01 | 0.100773 |  |
| 2015-07-01 | 0.117134 |  |
| 2015-08-01 | 0.0627503 |  |
| 2015-09-01 | 0.0962171 |  |
| 2015-10-01 | 0.0542015 |  |
| 2015-11-01 | 0.0333548 |  |
| 2015-12-01 | 0.108403 |  |
| 2016-01-01 | 0.0622318 |  |
| 2016-02-01 | 0.0456367 |  |
| 2016-03-01 | 0.0497855 |  |
| 2016-04-01 | 0.0454716 |  |
| 2016-05-01 | 0.0702743 |  |
| 2016-06-01 | 0.0661406 |  |
| 2016-07-01 | 0.0411564 |  |
| 2016-08-01 | 0.0740816 |  |
| 2016-09-01 | 0.0535034 |  |
| 2016-10-01 | 0.0451096 |  |
| 2016-11-01 | 0.0164035 |  |
| 2016-12-01 | 0.0574122 |  |
| 2017-01-01 | 0.0163189 |  |
| 2017-02-01 | 0.0122392 |  |
| 2017-03-01 | 0.0489568 |  |
| 2017-04-01 | 0.0325301 |  |
| 2017-05-01 | 0.0447289 |  |
| 2017-06-01 | 0.0447289 |  |
| 2017-07-01 | 0.0364519 |  |
| 2017-08-01 | 0.0243013 |  |
| 2017-09-01 | 0.0445523 |  |
| 2017-10-01 | 0.0444283 |  |
| 2017-11-01 | 0.0282725 |  |
| 2017-12-01 | 0.0161557 |  |
| 2018-01-01 | 0.0442091 |  |
| 2018-02-01 | 0.0763612 |  |
| 2018-03-01 | 0.0843992 |  |
| 2018-04-01 | 0.0440648 |  |
| 2018-05-01 | 0.0640942 |  |
| 2018-06-01 | 0.0320471 |  |
| 2018-07-01 | 0.0638279 |  |
| 2018-08-01 | 0.139624 |  |
| 2018-09-01 | 0.031914 |  |
| 2018-10-01 | 0.0676048 |  |
| 2018-11-01 | 0.103396 |  |
| 2018-12-01 | 0.0437443 |  |
| 2019-01-01 | 0.0870772 |  |
| 2019-02-01 | 0.0356225 |  |
| 2019-03-01 | 0.0593708 |  |
| 2019-04-01 | 0.0671013 |  |
| 2019-05-01 | 0.0710484 |  |
| 2019-06-01 | 0.0355242 |  |
| 2019-07-01 | 0.0825534 |  |
| 2019-08-01 | 0.0511045 |  |
| 2019-09-01 | 0.0707601 |  |
| 2019-10-01 | 0.0901237 |  |
| 2019-11-01 | 0.0509395 |  |
| 2019-12-01 | 0.0391842 |  |
| 2020-01-01 | 0.0663339 |  |
| 2020-02-01 | 0.0741379 |  |
| 2020-03-01 | 0.0624319 |  |
| 2020-04-01 | 0.0194937 |  |
| 2020-05-01 | 0.0740762 |  |
| 2020-06-01 | 0.0740762 | 0.0116962 |
| 2020-07-01 | 0.0546164 | 0.0117035 |
| 2020-08-01 | 0.0390117 | 0.0156047 |
| 2020-09-01 | 0.0351105 | 0.0117035 |
| 2020-10-01 | 0.0351141 | 0.0156063 |
| 2020-11-01 | 0.0312126 | 0.0195079 |
| 2020-12-01 | 0.0429173 | 0.0117047 |
| 2021-01-01 | 0.0194909 | 0.00779636 |
| 2021-02-01 | 0.0506763 |  |
| 2021-03-01 | 0.0194909 | 0.00389818 |
| 2021-04-01 | 0.0583989 | 0.00389326 |
| 2021-05-01 | 0.0272528 | 0.0116798 |
| 2021-06-01 | 0.0272528 | 0.00778652 |
| 2021-07-01 | 0.0311226 | 0.011671 |
| 2021-08-01 | 0.0311226 | 0.011671 |
| 2021-09-01 | 0.00389033 | 0.0155613 |
| 2021-10-01 | 0.0349192 |  |
| 2021-11-01 | 0.042679 |  |
| 2021-12-01 | 0.0232795 |  |
| 2022-01-01 | 0.0192954 |  |
| 2022-02-01 | 0.0154363 | 0.00771814 |
| 2022-03-01 | 0.0154363 | 0.00385907 |
| 2022-04-01 | 0.0192169 | 0.00384339 |
| 2022-05-01 | 0.0345905 | 0.00768677 |
| 2022-06-01 | 0.0307471 | 0.00384339 |
| 2022-07-01 | 0.0382127 | 0.00764254 |
| 2022-08-01 | 0.0305702 | 0.00764254 |
| 2022-09-01 | 0.0343914 |  |
| 2022-10-01 | 0.0379935 | 0.0075987 |
| 2022-11-01 | 0.0379935 |  |
| 2022-12-01 | 0.0303948 |  |
| 2023-01-01 | 0.0339439 | 0.0113146 |
| 2023-02-01 | 0.0829741 | 0.0113146 |
| 2023-03-01 | 0.0528017 | 0.0075431 |
| 2023-04-01 | 0.0562642 |  |
| 2023-05-01 | 0.0525133 |  |
| 2023-06-01 | 0.0525133 |  |
| 2023-07-01 | 0.0186351 | 0.00372701 |
| 2023-08-01 | 0.0670863 |  |
| 2023-09-01 | 0.0596322 | 0.011181 |
| 2023-10-01 | 0.0185483 | 0.00370966 |
| 2023-11-01 | 0.0370966 |  |
| 2023-12-01 | 0.0556449 | 0.00741932 |
| 2024-01-01 | 0.0737639 | 0.0036882 |
| 2024-02-01 | 0.0331938 | 0.0036882 |
| 2024-03-01 | 0.036882 | 0.0036882 |
| 2024-04-01 | 0.0514823 | 0.00367731 |
| 2024-05-01 | 0.047805 |  |
| 2024-06-01 | 0.0882554 |  |
| 2024-07-01 | 0.109876 |  |
| 2024-08-01 | 0.0439504 | 0.0146501 |
| 2024-09-01 | 0.0769132 |  |
| 2024-10-01 | 0.0547592 | 0.00730123 |
| 2024-11-01 | 0.0912654 | 0.0109518 |
| 2024-12-01 | 0.0803136 | 0.0109518 |
| 2025-01-01 | 0.130734 | 0.00726299 |
| 2025-02-01 | 0.0508409 |  |
| 2025-03-01 | 0.0399464 | 0.00726299 |
| 2025-04-01 | 0.0798929 | 0.00726299 |
| 2025-05-01 | 0.0907874 |  |
| 2025-06-01 | 0.0689984 | 0.00363149 |
| 2025-07-01 | 0.0435779 | 0.014526 |
| 2025-08-01 | 0.0871559 | 0.00726299 |

Dicloxacillin

|  | Dicloxacillin capsule 250 mg 05096F | Dicloxacillin capsule 500 mg 05097G |
| --- | --- | --- |
| 2014-02-01 | 0.00427237 | 0.0213619 |
| 2014-03-01 | 0.00854475 | 0.0256342 |
| 2014-04-01 | 0.00851945 | 0.0170389 |
| 2014-05-01 | 0.00425973 | 0.0212986 |
| 2014-06-01 | 0.00425973 | 0.0383375 |
| 2014-07-01 |  | 0.0127319 |
| 2014-08-01 |  | 0.0212198 |
| 2014-09-01 | 0.00424396 | 0.0127319 |
| 2014-10-01 |  | 0.0211503 |
| 2014-11-01 |  | 0.0253804 |
| 2014-12-01 | 0.00423006 | 0.0380705 |
| 2015-01-01 |  | 0.0589582 |
| 2015-02-01 |  | 0.0336904 |
| 2015-03-01 | 0.0042113 | 0.0336904 |
| 2015-04-01 |  | 0.029392 |
| 2015-05-01 |  | 0.0209943 |
| 2015-06-01 |  | 0.0209943 |
| 2015-07-01 | 0.00836671 | 0.0251001 |
| 2015-08-01 |  | 0.0167334 |
| 2015-09-01 |  | 0.0292835 |
| 2015-11-01 |  | 0.0250161 |
| 2015-12-01 |  | 0.0166774 |
| 2016-01-01 | 0.00414879 | 0.0207439 |
| 2016-02-01 | 0.00414879 | 0.0248927 |
| 2016-03-01 | 0.00829758 | 0.0373391 |
| 2016-04-01 |  | 0.0165351 |
| 2016-05-01 | 0.00413378 | 0.0206689 |
| 2016-06-01 |  | 0.0124014 |
| 2016-07-01 |  | 0.00823129 |
| 2016-08-01 |  | 0.0123469 |
| 2016-09-01 |  | 0.00823129 |
| 2016-10-01 |  | 0.0123026 |
| 2016-11-01 |  | 0.00410087 |
| 2016-12-01 |  | 0.0205044 |
| 2017-01-01 | 0.00407973 | 0.0163189 |
| 2017-02-01 |  | 0.00407973 |
| 2017-03-01 |  | 0.0163189 |
| 2017-04-01 |  | 0.00813253 |
| 2017-05-01 |  | 0.0243976 |
| 2017-06-01 |  | 0.0121988 |
| 2017-07-01 |  | 0.0162008 |
| 2017-08-01 |  | 0.00810042 |
| 2017-09-01 | 0.00405021 | 0.00405021 |
| 2017-10-01 |  | 0.00403893 |
| 2017-11-01 |  | 0.0201947 |
| 2017-12-01 |  | 0.0323115 |
| 2018-01-01 |  | 0.00803802 |
| 2018-02-01 |  | 0.00401901 |
| 2018-03-01 |  | 0.016076 |
| 2018-04-01 |  | 0.0120177 |
| 2018-05-01 |  | 0.00400589 |
| 2018-06-01 |  | 0.00400589 |
| 2018-07-01 |  | 0.015957 |
| 2018-08-01 |  | 0.00797849 |
| 2018-09-01 |  | 0.00398925 |
| 2018-10-01 |  | 0.0119303 |
| 2018-11-01 |  | 0.00795351 |
| 2018-12-01 |  | 0.00795351 |
| 2019-01-01 |  | 0.00395806 |
| 2019-02-01 |  | 0.00791611 |
| 2019-04-01 |  | 0.00394714 |
| 2019-07-01 |  | 0.0117933 |
| 2019-08-01 |  | 0.0196556 |
| 2019-09-01 |  | 0.0117933 |
| 2019-10-01 |  | 0.00783685 |
| 2019-11-01 |  | 0.0117553 |
| 2019-12-01 |  | 0.00391842 |
| 2020-01-01 |  | 0.00390199 |
| 2020-02-01 |  | 0.015608 |
| 2020-03-01 |  | 0.01951 |
| 2020-04-01 | 0.00389875 | 0.0077975 |
| 2020-05-01 |  | 0.0077975 |
| 2020-06-01 |  | 0.00389875 |
| 2020-07-01 |  | 0.0117035 |
| 2020-08-01 |  | 0.00390117 |
| 2020-09-01 | 0.00390117 | 0.00780234 |
| 2020-10-01 | 0.00390157 | 0.0156063 |
| 2020-11-01 |  | 0.0156063 |
| 2020-12-01 |  | 0.027311 |
| 2021-01-01 |  | 0.00779636 |
| 2021-02-01 |  | 0.0116945 |
| 2021-03-01 |  | 0.0155927 |
| 2021-04-01 |  | 0.015573 |
| 2021-05-01 |  | 0.0428259 |
| 2021-06-01 | 0.00778652 | 0.0428259 |
| 2021-07-01 |  | 0.0272323 |
| 2021-08-01 |  | 0.023342 |
| 2021-09-01 | 0.00389033 | 0.0155613 |
| 2021-10-01 |  | 0.0116397 |
| 2021-11-01 |  | 0.0193995 |
| 2021-12-01 |  | 0.0193995 |
| 2022-01-01 | 0.00385907 | 0.00385907 |
| 2022-02-01 |  | 0.0115772 |
| 2022-03-01 |  | 0.00385907 |
| 2022-04-01 |  | 0.0115302 |
| 2022-05-01 |  | 0.0153735 |
| 2022-06-01 | 0.00384339 | 0.00768677 |
| 2022-07-01 |  | 0.00764254 |
| 2022-08-01 |  | 0.0114638 |
| 2022-09-01 |  | 0.00382127 |
| 2022-10-01 |  | 0.0189968 |
| 2022-11-01 | 0.00379935 | 0.0113981 |
| 2022-12-01 |  | 0.0151974 |
| 2023-03-01 |  | 0.0113146 |
| 2023-05-01 |  | 0.00375095 |
| 2023-06-01 |  | 0.0075019 |
| 2023-07-01 |  | 0.0223621 |
| 2023-08-01 |  | 0.00745403 |
| 2023-09-01 |  | 0.00372701 |
| 2023-10-01 |  | 0.00741932 |
| 2023-11-01 |  | 0.011129 |
| 2024-02-01 | 0.0036882 | 0.0110646 |
| 2024-03-01 |  | 0.0110646 |
| 2024-04-01 |  | 0.0110319 |
| 2024-05-01 |  | 0.0147092 |
| 2024-06-01 | 0.00367731 | 0.00367731 |
| 2024-07-01 | 0.00732507 | 0.00732507 |
| 2024-08-01 | 0.00366253 | 0.00366253 |
| 2024-09-01 |  | 0.0109876 |
| 2024-11-01 |  | 0.00365062 |
| 2024-12-01 |  | 0.00365062 |
| 2025-01-01 |  | 0.00726299 |
| 2025-02-01 |  | 0.0181575 |
| 2025-03-01 |  | 0.0108945 |
| 2025-04-01 |  | 0.0108945 |
| 2025-05-01 |  | 0.014526 |
| 2025-06-01 |  | 0.00363149 |
| 2025-07-01 |  | 0.00363149 |
| 2025-08-01 | 0.00363149 | 0.00363149 |

Doxycycline

|  | Doxycycline capsule 100 mg (containing enteric coated pellets) 03322W | Doxycycline tablet 100 mg, 7 (as hyclate) 03321T | Doxycycline tablet 100 mg, 7 (as monohydrate) 05082L |
| --- | --- | --- | --- |
| 2014-02-01 | 0.260615 | 0.205074 | 0.281977 |
| 2014-03-01 | 0.299066 | 0.290521 | 0.3247 |
| 2014-04-01 | 0.247064 | 0.345038 | 0.340778 |
| 2014-05-01 | 0.285402 | 0.3067 | 0.332259 |
| 2014-06-01 | 0.272622 | 0.31096 | 0.383375 |
| 2014-07-01 | 0.275857 | 0.352249 | 0.394688 |
| 2014-08-01 | 0.24615 | 0.322541 | 0.377712 |
| 2014-09-01 | 0.195222 | 0.314053 | 0.40742 |
| 2014-10-01 | 0.228423 | 0.274954 | 0.346865 |
| 2014-11-01 | 0.186123 | 0.270724 | 0.342635 |
| 2014-12-01 | 0.258034 | 0.224193 | 0.397626 |
| 2015-01-01 | 0.134762 | 0.328482 | 0.286368 |
| 2015-02-01 | 0.147396 | 0.273735 | 0.273735 |
| 2015-03-01 | 0.155818 | 0.294791 | 0.362172 |
| 2015-04-01 | 0.167954 | 0.348505 | 0.373698 |
| 2015-05-01 | 0.10917 | 0.394693 | 0.310716 |
| 2015-06-01 | 0.167954 | 0.310716 | 0.285522 |
| 2015-07-01 | 0.150601 | 0.359768 | 0.267735 |
| 2015-08-01 | 0.192434 | 0.284468 | 0.439252 |
| 2015-09-01 | 0.192434 | 0.242634 | 0.368135 |
| 2015-10-01 | 0.233483 | 0.296024 | 0.350225 |
| 2015-11-01 | 0.158435 | 0.375241 | 0.37941 |
| 2015-12-01 | 0.187621 | 0.308532 | 0.329378 |
| 2016-01-01 | 0.149356 | 0.190844 | 0.207439 |
| 2016-02-01 | 0.186695 | 0.282118 | 0.294564 |
| 2016-03-01 | 0.165952 | 0.261374 | 0.261374 |
| 2016-04-01 | 0.165351 | 0.165351 | 0.367907 |
| 2016-05-01 | 0.157084 | 0.264562 | 0.289365 |
| 2016-06-01 | 0.095077 | 0.252161 | 0.343104 |
| 2016-07-01 | 0.069966 | 0.238707 | 0.403333 |
| 2016-08-01 | 0.0781972 | 0.251054 | 0.390986 |
| 2016-09-01 | 0.0740816 | 0.251054 | 0.218129 |
| 2016-10-01 | 0.0861183 | 0.250153 | 0.254254 |
| 2016-11-01 | 0.0779165 | 0.287061 | 0.319868 |
| 2016-12-01 | 0.114824 | 0.28296 | 0.278859 |
| 2017-01-01 | 0.061196 | 0.30598 | 0.261103 |
| 2017-02-01 | 0.0530365 | 0.228465 | 0.179508 |
| 2017-03-01 | 0.0448771 | 0.248864 | 0.224385 |
| 2017-04-01 | 0.0813253 | 0.195181 | 0.248042 |
| 2017-05-01 | 0.060994 | 0.300904 | 0.309036 |
| 2017-06-01 | 0.0650603 | 0.235843 | 0.243976 |
| 2017-07-01 | 0.105305 | 0.243013 | 0.287565 |
| 2017-08-01 | 0.0850544 | 0.267314 | 0.360469 |
| 2017-09-01 | 0.076954 | 0.255163 | 0.259214 |
| 2017-10-01 | 0.100973 | 0.234258 | 0.274647 |
| 2017-11-01 | 0.0646229 | 0.193869 | 0.26657 |
| 2017-12-01 | 0.0525061 | 0.254453 | 0.254453 |
| 2018-01-01 | 0.0401901 | 0.180855 | 0.204969 |
| 2018-02-01 | 0.0562661 | 0.217027 | 0.176836 |
| 2018-03-01 | 0.0643042 | 0.225065 | 0.237122 |
| 2018-04-01 | 0.0240353 | 0.160235 | 0.208306 |
| 2018-05-01 | 0.0841236 | 0.228336 | 0.216318 |
| 2018-06-01 | 0.072106 | 0.176259 | 0.240353 |
| 2018-07-01 | 0.0718064 | 0.21143 | 0.251322 |
| 2018-08-01 | 0.0877634 | 0.207441 | 0.243344 |
| 2018-09-01 | 0.031914 | 0.21143 | 0.235365 |
| 2018-10-01 | 0.0437443 | 0.190884 | 0.290303 |
| 2018-11-01 | 0.0676048 | 0.206791 | 0.274396 |
| 2018-12-01 | 0.0516978 | 0.14714 | 0.290303 |
| 2019-01-01 | 0.0514547 | 0.16228 | 0.209777 |
| 2019-02-01 | 0.0395806 | 0.229567 | 0.269148 |
| 2019-03-01 | 0.0237483 | 0.178112 | 0.205819 |
| 2019-04-01 | 0.0394714 | 0.169727 | 0.209198 |
| 2019-05-01 | 0.0828899 | 0.16578 | 0.240775 |
| 2019-06-01 | 0.0315771 | 0.149991 | 0.244722 |
| 2019-07-01 | 0.0432423 | 0.157245 | 0.267316 |
| 2019-08-01 | 0.0275178 | 0.129727 | 0.302696 |
| 2019-09-01 | 0.03538 | 0.10614 | 0.267316 |
| 2019-10-01 | 0.0431027 | 0.144982 | 0.286045 |
| 2019-11-01 | 0.0391842 | 0.203758 | 0.235105 |
| 2019-12-01 | 0.0509395 | 0.188084 | 0.219432 |
| 2020-01-01 | 0.0390199 | 0.148276 | 0.199002 |
| 2020-02-01 | 0.0390199 | 0.171688 | 0.191198 |
| 2020-03-01 | 0.0585299 | 0.179492 | 0.191198 |
| 2020-04-01 | 0.0233925 | 0.12476 | 0.105266 |
| 2020-05-01 | 0.0116962 | 0.152051 | 0.167646 |
| 2020-06-01 | 0.0389875 | 0.159849 | 0.15595 |
| 2020-07-01 | 0.0546164 | 0.163849 | 0.226268 |
| 2020-08-01 | 0.0429129 | 0.152146 | 0.261378 |
| 2020-09-01 | 0.0273082 | 0.214564 | 0.19896 |
| 2020-10-01 | 0.0390157 | 0.210685 | 0.300421 |
| 2020-11-01 | 0.0312126 | 0.171669 | 0.218488 |
| 2020-12-01 | 0.0351141 | 0.195079 | 0.245799 |
| 2021-01-01 | 0.0389818 | 0.140334 | 0.187113 |
| 2021-02-01 | 0.0584727 | 0.187113 | 0.167622 |
| 2021-03-01 | 0.0701672 | 0.183214 | 0.268974 |
| 2021-04-01 | 0.0467191 | 0.20245 | 0.194663 |
| 2021-05-01 | 0.0311461 | 0.175197 | 0.272528 |
| 2021-06-01 | 0.0428259 | 0.20245 | 0.276421 |
| 2021-07-01 | 0.0389033 | 0.194516 | 0.245091 |
| 2021-08-01 | 0.0739162 | 0.159503 | 0.23731 |
| 2021-09-01 | 0.0622452 | 0.202297 | 0.245091 |
| 2021-10-01 | 0.0504388 | 0.116397 | 0.217275 |
| 2021-11-01 | 0.0581986 | 0.170716 | 0.236674 |
| 2021-12-01 | 0.0310393 | 0.159076 | 0.221155 |
| 2022-01-01 | 0.0347316 | 0.119631 | 0.20839 |
| 2022-02-01 | 0.0347316 | 0.173658 | 0.20839 |
| 2022-03-01 | 0.054027 | 0.266276 | 0.192954 |
| 2022-04-01 | 0.0345905 | 0.288254 | 0.119145 |
| 2022-05-01 | 0.0576508 | 0.349748 | 0.103771 |
| 2022-06-01 | 0.0538074 | 0.376652 | 0.142205 |
| 2022-07-01 | 0.0649616 | 0.351557 | 0.168136 |
| 2022-08-01 | 0.0726041 | 0.45091 | 0.164315 |
| 2022-09-01 | 0.0878892 | 0.374484 | 0.187242 |
| 2022-10-01 | 0.0835857 | 0.402731 | 0.132977 |
| 2022-11-01 | 0.064589 | 0.395132 | 0.170971 |
| 2022-12-01 | 0.0493916 | 0.281152 | 0.148175 |
| 2023-01-01 | 0.0339439 | 0.26778 | 0.132004 |
| 2023-02-01 | 0.0226293 | 0.279095 | 0.158405 |
| 2023-03-01 | 0.0452586 | 0.328125 | 0.173491 |
| 2023-04-01 | 0.0262566 | 0.258815 | 0.112528 |
| 2023-05-01 | 0.0412604 | 0.311329 | 0.0975247 |
| 2023-06-01 | 0.0262566 | 0.337585 | 0.135034 |
| 2023-07-01 | 0.0484512 | 0.268345 | 0.201259 |
| 2023-08-01 | 0.0335431 | 0.234802 | 0.216167 |
| 2023-09-01 | 0.0260891 | 0.227348 | 0.1379 |
| 2023-10-01 | 0.0259676 | 0.226289 | 0.152096 |
| 2023-11-01 | 0.0259676 | 0.241128 | 0.10758 |
| 2023-12-01 | 0.0408062 | 0.315321 | 0.0630642 |
| 2024-01-01 | 0.0331938 | 0.287679 | 0.0147528 |
| 2024-02-01 | 0.018441 | 0.228668 | 0.118022 |
| 2024-03-01 | 0.036882 | 0.195474 | 0.110646 |
| 2024-04-01 | 0.0220639 | 0.253734 | 0.0772235 |
| 2024-05-01 | 0.0404504 | 0.242702 | 0.128706 |
| 2024-06-01 | 0.0257412 | 0.356699 | 0.0882554 |
| 2024-07-01 | 0.054938 | 0.322303 | 0.135514 |
| 2024-08-01 | 0.0329628 | 0.413866 | 0.0659256 |
| 2024-09-01 | 0.0293003 | 0.322303 | 0.0915634 |
| 2024-10-01 | 0.0292049 | 0.343158 | 0.113169 |
| 2024-11-01 | 0.0219037 | 0.219037 | 0.094916 |
| 2024-12-01 | 0.0146025 | 0.248242 | 0.146025 |
| 2025-01-01 | 0.0181575 | 0.254205 | 0.0544724 |
| 2025-02-01 | 0.00726299 | 0.246942 | 0.0653669 |
| 2025-03-01 | 0.0108945 | 0.286888 | 0.0472094 |
| 2025-04-01 | 0.0326834 | 0.272362 | 0.0435779 |
| 2025-05-01 | 0.0399464 | 0.257836 | 0.0363149 |
| 2025-06-01 | 0.014526 | 0.268731 | 0.0399464 |
| 2025-07-01 | 0.014526 | 0.294151 | 0.0798929 |
| 2025-08-01 | 0.0399464 | 0.31594 | 0.0581039 |

Erythromycin

|  | Erythromycin capsule 250 mg (containing enteric coated pellets) 03325B | Erythromycin oral liquid 200 mg per 5 mL, 100 mL 03334L | Erythromycin oral liquid 400 mg per 5 mL, 100 mL 03337P | Erythromycin tablet 400 mg 03336N |
| --- | --- | --- | --- | --- |
| 2014-02-01 | 2.8454 | 0.166623 | 0.0640856 | 1.93539 |
| 2014-03-01 | 3.03766 | 0.123899 | 0.0384514 | 1.98238 |
| 2014-04-01 | 3.16072 | 0.102233 | 0.0724153 | 1.8615 |
| 2014-05-01 | 2.98181 | 0.0851945 | 0.0425973 | 2.09153 |
| 2014-06-01 | 2.99459 | 0.0553764 | 0.046857 | 1.87428 |
| 2014-07-01 | 3.1957 | 0.123075 | 0.0679034 | 2.10076 |
| 2014-08-01 | 2.87316 | 0.123075 | 0.0763913 | 1.87159 |
| 2014-09-01 | 2.92409 | 0.127319 | 0.0721473 | 2.04559 |
| 2014-10-01 | 2.92297 | 0.135362 | 0.0296104 | 2.05581 |
| 2014-11-01 | 2.82145 | 0.109982 | 0.0549908 | 1.857 |
| 2014-12-01 | 2.84683 | 0.114212 | 0.0592208 | 2.07696 |
| 2015-01-01 | 2.312 | 0.138973 | 0.0463243 | 1.78559 |
| 2015-02-01 | 2.5352 | 0.122128 | 0.0589582 | 1.61714 |
| 2015-03-01 | 2.95633 | 0.0884373 | 0.0800147 | 1.8614 |
| 2015-04-01 | 2.5739 | 0.104971 | 0.0503863 | 1.62076 |
| 2015-05-01 | 2.5739 | 0.0839772 | 0.0503863 | 1.69214 |
| 2015-06-01 | 2.71666 | 0.142761 | 0.0461874 | 1.65435 |
| 2015-07-01 | 2.5393 | 0.0878504 | 0.0627503 | 1.84904 |
| 2015-08-01 | 2.5142 | 0.133867 | 0.0502002 | 1.68171 |
| 2015-09-01 | 2.43889 | 0.138051 | 0.0125501 | 1.90761 |
| 2015-10-01 | 2.55998 | 0.12508 | 0.0375241 | 1.59269 |
| 2015-11-01 | 2.1347 | 0.0958949 | 0.0542015 | 1.60103 |
| 2015-12-01 | 2.60167 | 0.133419 | 0.0583708 | 1.8637 |
| 2016-01-01 | 1.85866 | 0.0705294 | 0.0165952 | 1.36495 |
| 2016-02-01 | 2.43119 | 0.116166 | 0.0663806 | 1.70515 |
| 2016-03-01 | 2.33992 | 0.112017 | 0.0331903 | 1.67196 |
| 2016-04-01 | 2.32732 | 0.0909433 | 0.0372041 | 1.823 |
| 2016-05-01 | 2.49681 | 0.0826757 | 0.0537392 | 1.65351 |
| 2016-06-01 | 2.1661 | 0.0909433 | 0.0496054 | 1.57084 |
| 2016-07-01 | 2.08663 | 0.0905442 | 0.0658503 | 1.45282 |
| 2016-08-01 | 2.45292 | 0.102891 | 0.069966 | 1.57218 |
| 2016-09-01 | 2.3418 | 0.0823129 | 0.0617347 | 1.49809 |
| 2016-10-01 | 1.98072 | 0.09432 | 0.0451096 | 1.56243 |
| 2016-11-01 | 2.05864 | 0.0697148 | 0.0205044 | 1.50912 |
| 2016-12-01 | 2.19397 | 0.0615131 | 0.0738157 | 1.4107 |
| 2017-01-01 | 1.73389 | 0.0693555 | 0.0407973 | 1.2892 |
| 2017-02-01 | 1.85628 | 0.061196 | 0.0203987 | 1.41975 |
| 2017-03-01 | 2.15002 | 0.0407973 | 0.0367176 | 1.35855 |
| 2017-04-01 | 1.80136 | 0.0325301 | 0.0406627 | 1.24834 |
| 2017-05-01 | 1.98027 | 0.0975904 | 0.0325301 | 1.42726 |
| 2017-06-01 | 1.73223 | 0.0650603 | 0.0650603 | 1.18735 |
| 2017-07-01 | 1.8793 | 0.0445523 | 0.0324017 | 1.05305 |
| 2017-08-01 | 1.9279 | 0.101255 | 0.0202511 | 1.36087 |
| 2017-09-01 | 1.8631 | 0.101255 | 0.0324017 | 1.28797 |
| 2017-10-01 | 1.80136 | 0.0525061 | 0.0242336 | 1.31265 |
| 2017-11-01 | 1.8983 | 0.0484672 | 0.0484672 | 1.2763 |
| 2017-12-01 | 1.6075 | 0.060584 | 0.0403893 | 1.2965 |
| 2018-01-01 | 1.63172 | 0.0602851 | 0.0442091 | 1.07709 |
| 2018-02-01 | 1.70808 | 0.0482281 | 0.0321521 | 1.02887 |
| 2018-03-01 | 1.88492 | 0.0562661 | 0.0442091 | 1.16149 |
| 2018-04-01 | 1.58633 | 0.0560824 | 0.0240353 | 1.00147 |
| 2018-05-01 | 1.86274 | 0.0681001 | 0.0400589 | 1.17773 |
| 2018-06-01 | 1.75858 | 0.0400589 | 0.0280412 | 1.16571 |
| 2018-07-01 | 1.71139 | 0.0478709 | 0.015957 | 1.22869 |
| 2018-08-01 | 1.88292 | 0.0678172 | 0.00797849 | 1.2247 |
| 2018-09-01 | 1.61166 | 0.0359032 | 0.0239355 | 0.989333 |
| 2018-10-01 | 1.57479 | 0.031814 | 0.0357908 | 1.10951 |
| 2018-11-01 | 1.59468 | 0.0676048 | 0.0198838 | 1.07372 |
| 2018-12-01 | 1.73784 | 0.0238605 | 0.015907 | 1.12144 |
| 2019-01-01 | 1.40115 | 0.0316644 | 0.0197903 | 0.831192 |
| 2019-02-01 | 1.49219 | 0.0633289 | 0.0356225 | 1.05284 |
| 2019-03-01 | 1.69801 | 0.0474967 | 0.0435386 | 1.0568 |
| 2019-04-01 | 1.51175 | 0.0434185 | 0.0157885 | 0.990731 |
| 2019-05-01 | 1.67359 | 0.0473656 | 0.02763 | 1.16441 |
| 2019-06-01 | 1.42492 | 0.0434185 | 0.0315771 | 0.911788 |
| 2019-07-01 | 1.65893 | 0.0393112 | 0.03538 | 1.13216 |
| 2019-08-01 | 1.49775 | 0.0314489 | 0.03538 | 1.26582 |
| 2019-09-01 | 1.4781 | 0.0550356 | 0.0235867 | 1.03781 |
| 2019-10-01 | 1.60655 | 0.0626948 | 0.027429 | 1.12459 |
| 2019-11-01 | 1.489 | 0.0626948 | 0.0156737 | 0.865972 |
| 2019-12-01 | 1.42239 | 0.027429 | 0.00783685 | 0.963932 |
| 2020-01-01 | 1.1823 | 0.0390199 | 0.01951 | 0.827223 |
| 2020-02-01 | 1.24474 | 0.0351179 | 0.011706 | 0.901361 |
| 2020-03-01 | 1.35009 | 0.0390199 | 0.0429219 | 1.02232 |
| 2020-04-01 | 1.02147 | 0.0194937 | 0.046785 | 0.697876 |
| 2020-05-01 | 1.09555 | 0.0233925 | 0.0194937 | 0.857725 |
| 2020-06-01 | 1.45033 | 0.03119 |  | 0.888915 |
| 2020-07-01 | 1.40052 | 0.0351105 | 0.0195058 | 1.0104 |
| 2020-08-01 | 1.35761 | 0.046814 | 0.046814 | 1.02211 |
| 2020-09-01 | 1.47464 | 0.0429129 | 0.0351105 | 0.963589 |
| 2020-10-01 | 1.36165 | 0.0312126 | 0.0468189 | 0.990999 |
| 2020-11-01 | 1.31093 | 0.0468189 | 0.0312126 | 0.909066 |
| 2020-12-01 | 1.34994 | 0.0156063 | 0.027311 | 0.924672 |
| 2021-01-01 | 1.16945 | 0.0194909 | 0.0272873 | 0.842007 |
| 2021-02-01 | 1.15386 | 0.0116945 | 0.0194909 | 0.943359 |
| 2021-03-01 | 1.39555 | 0.0506763 | 0.0155927 | 0.0896581 |
| 2021-04-01 | 1.17576 | 0.0233596 | 0.0350393 | 0.0545056 |
| 2021-05-01 | 1.22638 | 0.0350393 | 0.0311461 | 0.0428259 |
| 2021-06-01 | 1.23806 | 0.0350393 | 0.0116798 | 0.0116798 |
| 2021-07-01 | 1.26436 | 0.035013 | 0.0194516 | 0.011671 |
| 2021-08-01 | 1.27603 | 0.023342 | 0.0466839 | 0.0194516 |
| 2021-09-01 | 1.33827 | 0.0466839 | 0.035013 | 0.0155613 |
| 2021-10-01 | 1.23769 | 0.0310393 | 0.0193995 | 0.00775982 |
| 2021-11-01 | 1.44333 | 0.0271594 | 0.0310393 |  |
| 2021-12-01 | 1.38901 | 0.0310393 | 0.00775982 |  |
| 2022-01-01 | 0.980204 | 0.0115772 | 0.0154363 |  |
| 2022-02-01 | 1.1693 | 0.0308726 | 0.0231544 |  |
| 2022-03-01 | 1.23104 | 0.0192954 | 0.0192954 |  |
| 2022-04-01 | 1.06846 | 0.0422773 | 0.0192169 |  |
| 2022-05-01 | 1.23757 | 0.0384339 | 0.0192169 |  |
| 2022-06-01 | 1.28753 | 0.0269037 | 0.0153735 |  |
| 2022-07-01 | 1.22663 | 0.0343914 | 0.0229276 |  |
| 2022-08-01 | 1.49412 | 0.0229276 | 0.0382127 |  |
| 2022-09-01 | 1.43298 | 0.0191063 | 0.0152851 |  |
| 2022-10-01 | 1.34117 | 0.0379935 | 0.0189968 |  |
| 2022-11-01 | 1.41336 | 0.0455922 | 0.0189968 |  |
| 2022-12-01 | 1.39816 | 0.0455922 | 0.0265955 |  |
| 2023-01-01 | 1.02586 | 0.041487 | 0.0301724 |  |
| 2023-02-01 | 1.13901 | 0.0226293 | 0.0339439 |  |
| 2023-03-01 | 1.16164 | 0.0452586 | 0.00377155 |  |
| 2023-04-01 | 1.10653 | 0.0075019 | 0.0187547 |  |
| 2023-05-01 | 1.1703 | 0.0112528 | 0.0225057 |  |
| 2023-06-01 | 1.1928 | 0.0187547 | 0.0112528 |  |
| 2023-07-01 | 1.03238 | 0.0223621 | 0.00745403 |  |
| 2023-08-01 | 1.30073 | 0.0260891 | 0.011181 |  |
| 2023-09-01 | 0.946662 | 0.0260891 | 0.0149081 |  |
| 2023-10-01 | 1.02758 | 0.022258 | 0.0185483 |  |
| 2023-11-01 | 1.13887 | 0.0259676 | 0.011129 |  |
| 2023-12-01 | 0.945963 | 0.0259676 | 0.0148386 |  |
| 2024-01-01 | 0.85935 | 0.0036882 | 0.00737639 |  |
| 2024-02-01 | 0.984749 | 0.018441 | 0.0147528 |  |
| 2024-03-01 | 1.01057 | 0.018441 | 0.0147528 |  |
| 2024-04-01 | 0.963455 | 0.0257412 | 0.00367731 |  |
| 2024-05-01 | 1.12893 | 0.0183866 | 0.0183866 |  |
| 2024-06-01 | 0.930359 | 0.0147092 | 0.0257412 |  |
| 2024-07-01 | 0.908309 | 0.0293003 | 0.0329628 |  |
| 2024-08-01 | 1.0072 | 0.0402879 |  |  |
| 2024-09-01 | 0.904646 | 0.0256377 | 0.00732507 |  |
| 2024-10-01 | 0.806786 | 0.00365062 | 0.0219037 |  |
| 2024-11-01 | 0.865196 | 0.0146025 | 0.0146025 |  |
| 2024-12-01 | 0.82869 | 0.0182531 | 0.00730123 |  |
| 2025-01-01 | 0.751719 | 0.00726299 | 0.0108945 |  |
| 2025-02-01 | 0.744456 | 0.0181575 | 0.00363149 |  |
| 2025-03-01 | 0.737193 | 0.0108945 | 0.0108945 |  |
| 2025-04-01 | 0.904242 | 0.014526 | 0.00726299 |  |
| 2025-05-01 | 0.929663 | 0.0181575 | 0.0108945 |  |
| 2025-06-01 | 0.871559 | 0.0108945 | 0.00363149 |  |
| 2025-07-01 | 0.980503 | 0.0108945 | 0.00363149 |  |
| 2025-08-01 | 0.758982 | 0.0108945 | 0.0108945 |  |

Floxacillin

|  | Floxacillin capsule 250 mg 05090X | Floxacillin capsule 500 mg 05091Y | Floxacillin capsule 500 mg 11728G | Floxacillin injection 1 g 05095E | Floxacillin powder for oral liquid 125 mg per 5 mL, 100 mL 05257Q | Floxacillin oral liquid 250 mg per 5 mL, 100 mL 05258R |
| --- | --- | --- | --- | --- | --- | --- |
| 2014-02-01 | 0.034179 | 0.0897198 |  |  |  | 0.0170895 |
| 2014-03-01 | 0.0384514 | 0.0939922 |  |  | 0.00427237 | 0.00854475 |
| 2014-04-01 | 0.0127792 | 0.106493 |  |  | 0.00425973 | 0.0170389 |
| 2014-05-01 | 0.00851945 | 0.0766751 |  |  |  | 0.0212986 |
| 2014-06-01 | 0.0127792 | 0.0766751 |  |  |  | 0.00851945 |
| 2014-07-01 | 0.0381956 | 0.106099 |  |  |  | 0.00424396 |
| 2014-08-01 | 0.00424396 | 0.114587 |  |  |  |  |
| 2014-09-01 | 0.00424396 | 0.101855 |  |  |  | 0.00848792 |
| 2014-10-01 | 0.0253804 | 0.0972914 |  |  | 0.00423006 |  |
| 2014-11-01 | 0.0253804 | 0.156512 |  |  |  | 0.00423006 |
| 2014-12-01 | 0.00846012 | 0.131132 |  |  | 0.00423006 | 0.00846012 |
| 2015-01-01 | 0.0252678 | 0.0884373 |  |  | 0.0042113 | 0.0042113 |
| 2015-02-01 | 0.0168452 | 0.101071 |  |  |  |  |
| 2015-03-01 | 0.0084226 | 0.0884373 |  |  |  | 0.0126339 |
| 2015-04-01 |  | 0.138562 |  |  |  | 0.00419886 |
| 2015-05-01 | 0.0167954 | 0.0839772 |  |  |  | 0.0125966 |
| 2015-06-01 | 0.00419886 | 0.10917 |  |  |  |  |
| 2015-07-01 | 0.0251001 | 0.150601 |  |  |  | 0.00418335 |
| 2015-08-01 | 0.0125501 | 0.121317 |  |  |  | 0.0167334 |
| 2015-09-01 | 0.00836671 | 0.0836671 |  |  |  |  |
| 2015-10-01 | 0.00416935 | 0.0958949 |  |  |  | 0.00833869 |
| 2015-11-01 | 0.0166774 | 0.0792176 |  | 0.00416935 | 0.00416935 | 0.00833869 |
| 2015-12-01 | 0.012508 | 0.104234 |  |  | 0.00416935 | 0.00833869 |
| 2016-01-01 | 0.0124464 | 0.128612 |  |  |  |  |
| 2016-02-01 | 0.0165952 | 0.112017 |  |  | 0.00414879 | 0.00829758 |
| 2016-03-01 | 0.00414879 | 0.178398 |  |  |  | 0.00829758 |
| 2016-04-01 | 0.0248027 | 0.0620068 |  |  |  | 0.00826757 |
| 2016-05-01 | 0.0330703 | 0.111612 |  |  | 0.0124014 | 0.0124014 |
| 2016-06-01 | 0.00826757 | 0.095077 |  |  |  | 0.00826757 |
| 2016-07-01 | 0.00823129 | 0.069966 |  |  |  |  |
| 2016-08-01 |  | 0.139932 |  |  |  | 0.00411564 |
| 2016-09-01 | 0.0164626 | 0.0864285 |  |  |  | 0.00411564 |
| 2016-10-01 | 0.0164035 | 0.0615131 |  |  |  | 0.00410087 |
| 2016-11-01 | 0.00820174 | 0.0779165 |  |  |  | 0.00410087 |
| 2016-12-01 | 0.0246052 | 0.09432 |  |  |  | 0.00820174 |
| 2017-01-01 | 0.0122392 | 0.0775149 |  | 0.0122392 |  | 0.00407973 |
| 2017-02-01 | 0.0122392 | 0.0856744 |  |  | 0.00407973 |  |
| 2017-03-01 | 0.0122392 | 0.0979136 |  |  |  | 0.00407973 |
| 2017-04-01 |  | 0.0935241 |  |  |  | 0.00406627 |
| 2017-05-01 |  | 0.113855 |  |  | 0.00406627 | 0.00406627 |
| 2017-06-01 | 0.00813253 | 0.105723 |  |  |  | 0.00406627 |
| 2017-07-01 | 0.00405021 | 0.121506 |  |  |  | 0.00405021 |
| 2017-08-01 | 0.0121506 | 0.0931549 |  |  |  |  |
| 2017-09-01 | 0.00405021 | 0.121506 |  |  |  |  |
| 2017-10-01 | 0.00403893 | 0.0969344 |  |  |  | 0.00807786 |
| 2017-11-01 | 0.0121168 | 0.100973 |  |  |  | 0.00403893 |
| 2017-12-01 | 0.00403893 | 0.0686619 |  |  |  | 0.0161557 |
| 2018-01-01 | 0.00803802 | 0.0964562 |  |  |  | 0.00401901 |
| 2018-02-01 | 0.00803802 | 0.0924372 |  |  | 0.00803802 |  |
| 2018-03-01 | 0.016076 | 0.0964562 |  |  |  |  |
| 2018-04-01 | 0.0120177 | 0.0921354 |  |  |  | 0.00400589 |
| 2018-05-01 | 0.0320471 | 0.0841236 |  |  | 0.00400589 |  |
| 2018-06-01 | 0.0120177 | 0.100147 |  |  |  | 0.00400589 |
| 2018-07-01 | 0.0119677 | 0.10771 |  |  |  | 0.00398925 |
| 2018-08-01 | 0.00797849 | 0.0917526 |  |  |  |  |
| 2018-09-01 | 0.0119677 | 0.0877634 |  |  | 0.00398925 |  |
| 2018-10-01 | 0.0119303 | 0.0755583 |  |  |  |  |
| 2018-11-01 | 0.00795351 | 0.0994188 |  |  |  | 0.00397675 |
| 2018-12-01 | 0.0238605 | 0.111349 |  |  |  |  |
| 2019-01-01 | 0.00395806 | 0.0870772 |  |  |  |  |
| 2019-02-01 | 0.00395806 | 0.0831192 |  |  |  |  |
| 2019-03-01 | 0.00395806 | 0.114784 |  |  |  |  |
| 2019-04-01 | 0.0197357 | 0.0671013 |  |  |  |  |
| 2019-05-01 |  | 0.0552599 |  |  |  |  |
| 2019-06-01 | 0.00394714 | 0.0907841 |  |  |  |  |
| 2019-07-01 |  | 0.0707601 |  |  |  | 0.00786223 |
| 2019-08-01 |  | 0.0707601 |  |  |  |  |
| 2019-09-01 |  | 0.0550356 |  |  |  | 0.00393112 |
| 2019-10-01 | 0.0156737 | 0.0783685 |  |  |  | 0.0117553 |
| 2019-11-01 | 0.00783685 | 0.0979606 |  |  |  |  |
| 2019-12-01 | 0.00391842 | 0.109716 |  |  |  | 0.00391842 |
| 2020-01-01 | 0.011706 | 0.0975498 |  |  |  | 0.00390199 |
| 2020-02-01 | 0.00390199 | 0.0780399 |  |  | 0.00390199 | 0.00390199 |
| 2020-03-01 | 0.011706 | 0.101452 |  |  |  |  |
| 2020-04-01 |  | 0.077975 |  |  |  | 0.0077975 |
| 2020-05-01 | 0.00389875 | 0.0662787 |  |  |  |  |
| 2020-06-01 | 0.015595 | 0.09357 |  |  |  | 0.00389875 |
| 2020-07-01 | 0.0156047 | 0.0507152 |  |  |  |  |
| 2020-08-01 | 0.0156047 | 0.0624187 |  |  |  |  |
| 2020-09-01 | 0.00780234 | 0.070221 |  |  |  |  |
| 2020-10-01 | 0.0156063 | 0.054622 |  |  |  |  |
| 2020-11-01 | 0.00780314 | 0.0780314 | 0.00390157 |  |  |  |
| 2020-12-01 | 0.00390157 | 0.120949 |  |  |  |  |
| 2021-01-01 |  | 0.0701672 |  |  |  |  |
| 2021-02-01 |  | 0.0740654 |  |  |  | 0.00779636 |
| 2021-03-01 | 0.0155927 | 0.0818618 |  |  |  |  |
| 2021-04-01 | 0.0116798 | 0.112905 |  |  |  |  |
| 2021-05-01 | 0.00389326 | 0.0817585 |  |  |  |  |
| 2021-06-01 | 0.00389326 | 0.147944 |  |  |  | 0.0116798 |
| 2021-07-01 | 0.0155613 | 0.0933679 |  |  |  | 0.00389033 |
| 2021-08-01 | 0.00778066 | 0.0894775 |  |  |  |  |
| 2021-09-01 |  | 0.0622452 |  |  |  |  |
| 2021-10-01 | 0.0116397 | 0.0659585 |  |  |  |  |
| 2021-11-01 |  | 0.100878 |  |  |  | 0.00387991 |
| 2021-12-01 | 0.00387991 | 0.0659585 |  |  |  |  |
| 2022-01-01 | 0.00385907 | 0.0848995 |  |  |  |  |
| 2022-02-01 | 0.00385907 | 0.0771814 |  |  |  |  |
| 2022-03-01 |  | 0.119631 |  |  | 0.00385907 | 0.00385907 |
| 2022-04-01 | 0.00384339 | 0.0730243 |  |  |  |  |
| 2022-05-01 | 0.00384339 | 0.107615 |  |  |  | 0.00384339 |
| 2022-06-01 | 0.0115302 | 0.0922413 |  |  |  | 0.00384339 |
| 2022-07-01 | 0.00382127 | 0.099353 |  |  |  |  |
| 2022-08-01 | 0.00764254 | 0.114638 |  |  |  |  |
| 2022-09-01 |  | 0.0802467 |  |  |  |  |
| 2022-10-01 | 0.00379935 | 0.106382 |  |  |  | 0.00379935 |
| 2022-11-01 |  | 0.102582 |  |  |  | 0.0075987 |
| 2022-12-01 | 0.0151974 | 0.125379 |  |  |  | 0.00379935 |
| 2023-01-01 | 0.00377155 | 0.0942887 |  |  |  | 0.00377155 |
| 2023-02-01 | 0.00377155 | 0.0641163 |  |  |  |  |
| 2023-03-01 | 0.0075431 | 0.0980603 |  |  |  |  |
| 2023-04-01 | 0.0225057 | 0.071268 |  |  | 0.00375095 |  |
| 2023-05-01 |  | 0.0825209 |  |  |  | 0.00375095 |
| 2023-06-01 | 0.00375095 | 0.0450114 |  |  |  |  |
| 2023-07-01 | 0.00745403 | 0.0708133 |  |  |  |  |
| 2023-08-01 | 0.00372701 | 0.0521782 |  |  |  |  |
| 2023-09-01 | 0.00372701 | 0.0372701 |  |  |  |  |
| 2023-10-01 | 0.00370966 | 0.0890318 |  |  |  |  |
| 2023-11-01 | 0.011129 | 0.0556449 |  |  | 0.00370966 | 0.011129 |
| 2023-12-01 | 0.00741932 | 0.118709 |  |  |  | 0.00370966 |
| 2024-01-01 | 0.00737639 | 0.0774521 |  |  |  |  |
| 2024-02-01 | 0.0147528 | 0.0663876 |  |  |  |  |
| 2024-03-01 | 0.0110646 | 0.0922049 |  |  |  |  |
| 2024-04-01 | 0.00367731 | 0.058837 |  |  |  | 0.00367731 |
| 2024-05-01 | 0.0110319 | 0.0625143 |  |  |  | 0.00735462 |
| 2024-06-01 | 0.00735462 | 0.0661916 |  |  |  |  |
| 2024-07-01 | 0.00366253 | 0.0842383 |  |  | 0.00366253 | 0.00366253 |
| 2024-08-01 | 0.00732507 | 0.0769132 |  |  |  |  |
| 2024-09-01 | 0.00366253 | 0.0915634 |  |  |  | 0.00366253 |
| 2024-10-01 | 0.00730123 | 0.11682 |  |  |  |  |
| 2024-11-01 | 0.00365062 | 0.0730123 |  |  |  |  |
| 2024-12-01 |  | 0.0657111 |  |  |  |  |
| 2025-01-01 |  | 0.0835244 |  |  |  |  |
| 2025-02-01 | 0.00363149 | 0.101682 |  |  |  |  |
| 2025-03-01 | 0.00363149 | 0.0689984 |  |  | 0.00363149 | 0.00363149 |
| 2025-04-01 | 0.00363149 | 0.0617354 |  |  |  |  |
| 2025-05-01 |  | 0.0435779 |  |  |  | 0.00363149 |
| 2025-06-01 |  | 0.0508409 |  |  |  |  |
| 2025-07-01 |  | 0.0835244 |  |  |  |  |
| 2025-08-01 |  | 0.0653669 |  |  |  | 0.00363149 |

Glucagon

|  | Glucagon injection 1 mg (1 I.U.) and 1 mL solvent 05105Q |
| --- | --- |
| 2014-06-01 | 0.00425973 |
| 2014-07-01 | 0.00424396 |
| 2014-08-01 | 0.00424396 |
| 2014-10-01 | 0.00846012 |
| 2015-03-01 | 0.0084226 |
| 2015-04-01 | 0.00419886 |
| 2015-05-01 | 0.00419886 |
| 2015-06-01 | 0.00419886 |
| 2015-09-01 | 0.00418335 |
| 2015-10-01 | 0.00416935 |
| 2015-11-01 | 0.00416935 |
| 2016-07-01 | 0.00823129 |
| 2016-08-01 | 0.00411564 |
| 2016-10-01 | 0.00820174 |
| 2016-11-01 | 0.00410087 |
| 2017-01-01 | 0.00407973 |
| 2017-04-01 | 0.00813253 |
| 2017-05-01 | 0.00406627 |
| 2017-07-01 | 0.00405021 |
| 2017-12-01 | 0.00403893 |
| 2018-07-01 | 0.00797849 |
| 2019-01-01 | 0.00395806 |
| 2019-03-01 | 0.00395806 |
| 2019-05-01 | 0.00789427 |
| 2019-07-01 | 0.00393112 |
| 2019-09-01 | 0.00786223 |
| 2020-05-01 | 0.00389875 |
| 2020-08-01 | 0.00390117 |
| 2020-11-01 | 0.00390157 |
| 2021-02-01 | 0.00389818 |
| 2021-05-01 | 0.00389326 |
| 2021-10-01 | 0.00387991 |
| 2022-03-01 | 0.00771814 |
| 2022-08-01 | 0.00382127 |
| 2023-03-01 | 0.00377155 |
| 2023-09-01 | 0.00372701 |
| 2024-07-01 | 0.00366253 |
| 2024-09-01 | 0.00732507 |
| 2025-01-01 | 0.00363149 |
| 2025-03-01 | 0.00363149 |

Glucose with sodium chloride

|  | Glucose with sodium chloride IV infusion 31 mmol-222 mmol per L, 1 L 05214K |
| --- | --- |
| 2015-03-01 | 0.0042113 |

Glyceryl trinitrate

|  | Glyceryl trinitrate tablets 600 micrograms, 100 05108W |
| --- | --- |
| 2014-07-01 | 0.00424396 |
| 2015-01-01 | 0.0042113 |
| 2015-04-01 | 0.00419886 |
| 2016-07-01 | 0.00411564 |
| 2016-09-01 | 0.00823129 |
| 2017-04-01 | 0.00406627 |
| 2018-07-01 | 0.00398925 |

Hydrocortisone

|  | Hydrocortisone cream 10 mg per g, 30 g 05111B | Hydrocortisone cream 10 mg per g, 50 g 05113D | Hydrocortisone ointment 10 mg per g, 30 g 05112C | Hydrocortisone ointment 10 mg per g, 50 g 05114E | Hydrocortisone injection 100 mg with 2 mL solvent 05118J |
| --- | --- | --- | --- | --- | --- |
| 2014-02-01 | 0.00427237 | 0.00427237 | 0.0170895 | 0.00854475 |  |
| 2014-03-01 | 0.00854475 |  | 0.0170895 |  |  |
| 2014-04-01 |  |  | 0.0170389 | 0.00425973 |  |
| 2014-05-01 | 0.00425973 | 0.00425973 | 0.0127792 | 0.00425973 | 0.00425973 |
| 2014-06-01 | 0.0127792 | 0.00425973 | 0.00851945 | 0.00425973 |  |
| 2014-07-01 | 0.00424396 | 0.00424396 | 0.0127319 |  |  |
| 2014-08-01 |  | 0.00848792 | 0.0169758 | 0.00848792 | 0.00848792 |
| 2014-09-01 | 0.0127319 | 0.0169758 | 0.0127319 | 0.00848792 |  |
| 2014-10-01 | 0.0169202 |  | 0.0169202 | 0.00423006 |  |
| 2014-11-01 | 0.00846012 | 0.0211503 | 0.00846012 | 0.00423006 | 0.00423006 |
| 2014-12-01 | 0.0211503 | 0.0211503 | 0.0211503 | 0.0126902 |  |
| 2015-01-01 | 0.0126339 | 0.0084226 | 0.0084226 | 0.0168452 |  |
| 2015-02-01 |  | 0.0084226 | 0.0084226 | 0.0084226 |  |
| 2015-03-01 | 0.0042113 | 0.0084226 | 0.0084226 | 0.0126339 |  |
| 2015-04-01 | 0.00839772 | 0.0167954 | 0.00839772 |  | 0.00419886 |
| 2015-05-01 | 0.00419886 |  | 0.00839772 | 0.00839772 |  |
| 2015-06-01 | 0.00419886 | 0.00839772 | 0.0167954 | 0.00419886 |  |
| 2015-07-01 | 0.00836671 | 0.0167334 | 0.0125501 | 0.00418335 |  |
| 2015-08-01 | 0.0167334 | 0.00418335 | 0.00418335 | 0.00418335 |  |
| 2015-09-01 |  |  | 0.00836671 | 0.00836671 |  |
| 2015-10-01 | 0.00416935 | 0.00416935 | 0.00833869 | 0.00833869 |  |
| 2015-11-01 | 0.00416935 | 0.012508 | 0.00833869 | 0.00416935 |  |
| 2015-12-01 | 0.00416935 |  | 0.00833869 | 0.00833869 |  |
| 2016-01-01 |  | 0.0124464 |  |  |  |
| 2016-02-01 |  | 0.0165952 |  |  |  |
| 2016-03-01 |  | 0.00414879 |  | 0.00414879 |  |
| 2016-04-01 |  | 0.00826757 |  | 0.0165351 |  |
| 2016-05-01 |  | 0.00413378 |  | 0.00413378 | 0.00413378 |
| 2016-06-01 |  | 0.0165351 |  | 0.0124014 | 0.00413378 |
| 2016-07-01 |  | 0.00823129 |  | 0.00411564 |  |
| 2016-08-01 |  | 0.0164626 |  | 0.00411564 |  |
| 2016-09-01 |  | 0.0123469 |  | 0.00411564 | 0.00411564 |
| 2016-10-01 |  | 0.00820174 |  | 0.0123026 | 0.00410087 |
| 2016-11-01 |  | 0.00410087 |  | 0.0123026 |  |
| 2016-12-01 |  | 0.0287061 |  | 0.0205044 | 0.00410087 |
| 2017-01-01 |  | 0.00407973 |  |  |  |
| 2017-02-01 |  | 0.0122392 |  | 0.00407973 |  |
| 2017-03-01 |  | 0.0163189 |  | 0.00407973 |  |
| 2017-04-01 |  |  |  | 0.00406627 |  |
| 2017-05-01 |  | 0.00406627 |  | 0.0121988 |  |
| 2017-06-01 |  | 0.00813253 |  | 0.00813253 |  |
| 2017-07-01 |  | 0.00405021 |  | 0.00405021 | 0.00405021 |
| 2017-08-01 |  |  |  | 0.00405021 |  |
| 2017-09-01 |  |  |  | 0.00810042 |  |
| 2017-11-01 |  | 0.0121168 |  | 0.00403893 | 0.0121168 |
| 2017-12-01 |  | 0.00807786 |  |  |  |
| 2018-01-01 |  | 0.00401901 |  | 0.00803802 |  |
| 2018-02-01 |  | 0.00401901 |  | 0.016076 |  |
| 2018-03-01 |  | 0.00803802 |  |  |  |
| 2018-04-01 |  | 0.00801177 |  |  |  |
| 2018-05-01 |  | 0.00801177 |  | 0.0120177 | 0.00400589 |
| 2018-06-01 |  | 0.00400589 |  | 0.00801177 |  |
| 2018-07-01 |  | 0.0119677 |  | 0.00398925 |  |
| 2018-08-01 |  | 0.00797849 |  | 0.00797849 |  |
| 2018-09-01 |  | 0.00398925 |  | 0.00797849 |  |
| 2018-10-01 |  | 0.0119303 |  | 0.00795351 |  |
| 2018-11-01 |  | 0.0119303 |  | 0.00397675 |  |
| 2018-12-01 |  | 0.00795351 |  | 0.00397675 |  |
| 2019-02-01 |  | 0.0158322 |  | 0.00395806 |  |
| 2019-03-01 |  | 0.0118742 |  |  | 0.00395806 |
| 2019-04-01 |  | 0.0157885 |  |  |  |
| 2019-05-01 |  | 0.00789427 |  |  |  |
| 2019-06-01 |  | 0.00394714 |  |  |  |
| 2019-07-01 |  | 0.00393112 |  | 0.00786223 |  |
| 2019-08-01 |  | 0.00393112 |  |  |  |
| 2019-09-01 |  | 0.0157245 |  | 0.00393112 | 0.00393112 |
| 2019-10-01 |  |  |  | 0.00783685 |  |
| 2019-11-01 |  | 0.00391842 |  |  |  |
| 2019-12-01 |  | 0.00783685 |  | 0.00391842 |  |
| 2020-01-01 |  | 0.011706 |  |  |  |
| 2020-02-01 |  | 0.00390199 |  | 0.015608 |  |
| 2020-03-01 |  | 0.015608 |  | 0.011706 | 0.00780399 |
| 2020-04-01 |  | 0.00389875 |  | 0.00389875 |  |
| 2020-05-01 |  | 0.015595 |  | 0.00389875 |  |
| 2020-06-01 |  | 0.015595 |  | 0.015595 |  |
| 2020-07-01 |  | 0.00780234 |  | 0.0117035 |  |
| 2020-08-01 |  | 0.0117035 |  | 0.00780234 |  |
| 2020-09-01 |  | 0.0117035 |  | 0.0156047 |  |
| 2020-10-01 |  | 0.0234094 |  | 0.00390157 | 0.00390157 |
| 2020-12-01 |  | 0.0195079 |  | 0.00390157 |  |
| 2021-01-01 |  | 0.00779636 |  | 0.00779636 |  |
| 2021-02-01 |  | 0.00389818 |  |  | 0.00389818 |
| 2021-03-01 |  | 0.0116945 |  | 0.00389818 |  |
| 2021-04-01 |  | 0.00389326 |  | 0.00389326 |  |
| 2021-05-01 |  | 0.00778652 |  | 0.00778652 | 0.00778652 |
| 2021-06-01 |  | 0.00389326 |  | 0.00389326 |  |
| 2021-07-01 |  |  |  | 0.00389033 |  |
| 2021-08-01 |  | 0.0272323 |  | 0.023342 |  |
| 2021-09-01 |  | 0.00778066 |  | 0.00389033 |  |
| 2021-10-01 |  | 0.0116397 |  | 0.00387991 |  |
| 2021-11-01 |  | 0.0116397 |  | 0.00775982 |  |
| 2021-12-01 |  | 0.00775982 |  |  |  |
| 2022-01-01 |  |  |  | 0.0115772 |  |
| 2022-02-01 |  | 0.0115772 |  |  |  |
| 2022-03-01 |  | 0.00771814 |  | 0.0115772 |  |
| 2022-04-01 |  | 0.0192169 |  | 0.0115302 |  |
| 2022-05-01 |  | 0.0115302 |  |  |  |
| 2022-06-01 |  | 0.0192169 |  | 0.0115302 |  |
| 2022-07-01 |  |  |  | 0.00764254 |  |
| 2022-08-01 |  | 0.0191063 |  |  |  |
| 2022-09-01 |  | 0.00764254 |  | 0.00382127 |  |
| 2022-10-01 |  | 0.0075987 |  |  |  |
| 2022-11-01 |  | 0.0075987 |  | 0.00379935 |  |
| 2022-12-01 |  | 0.0113981 |  | 0.0113981 |  |
| 2023-01-01 |  | 0.0113146 |  | 0.00377155 |  |
| 2023-02-01 |  | 0.00377155 |  | 0.00377155 |  |
| 2023-03-01 |  | 0.0188577 |  | 0.0075431 |  |
| 2023-04-01 |  | 0.0112528 |  | 0.0075019 |  |
| 2023-05-01 |  | 0.0150038 |  |  |  |
| 2023-06-01 |  | 0.0075019 |  | 0.0075019 |  |
| 2023-07-01 |  | 0.011181 |  | 0.0149081 |  |
| 2023-08-01 |  | 0.00372701 |  |  |  |
| 2023-09-01 |  | 0.011181 |  | 0.0149081 |  |
| 2023-10-01 |  | 0.0148386 |  | 0.00741932 |  |
| 2023-11-01 |  | 0.00370966 |  | 0.00370966 |  |
| 2023-12-01 |  |  |  | 0.00370966 | 0.00370966 |
| 2024-02-01 |  | 0.0036882 |  |  |  |
| 2024-03-01 |  |  |  | 0.0036882 |  |
| 2024-06-01 |  | 0.00367731 |  |  |  |
| 2024-07-01 |  | 0.00366253 |  | 0.00366253 |  |
| 2024-09-01 |  | 0.00366253 |  |  |  |
| 2024-10-01 |  | 0.0146025 |  |  |  |
| 2024-11-01 |  | 0.00730123 |  | 0.00365062 |  |
| 2024-12-01 |  | 0.00365062 |  |  |  |
| 2025-01-01 |  |  |  | 0.00363149 |  |
| 2025-02-01 |  | 0.0108945 |  |  |  |
| 2025-04-01 |  |  |  | 0.00363149 |  |
| 2025-06-01 |  | 0.00726299 |  | 0.00363149 |  |
| 2025-07-01 |  | 0.014526 |  |  |  |
| 2025-08-01 |  | 0.00363149 |  |  |  |

Hydromorphone

|  | Hydromorphone oral liquid 1 mg per mL, 473 mL 05132D | Hydromorphone tablet 2 mg 05115F | Hydromorphone 4 mg 05116G | Hydromorphone tablet 8 mg 05117H |
| --- | --- | --- | --- | --- |
| 2014-11-01 |  | 0.00423006 |  |  |
| 2015-11-01 |  |  | 0.00416935 |  |
| 2016-02-01 |  | 0.00414879 |  |  |
| 2016-04-01 | 0.00413378 |  |  |  |
| 2016-07-01 |  |  |  | 0.00411564 |
| 2016-09-01 |  | 0.00411564 |  |  |
| 2016-12-01 | 0.00410087 |  |  |  |
| 2017-05-01 |  | 0.00406627 |  |  |
| 2018-07-01 |  | 0.00398925 |  |  |
| 2020-02-01 |  | 0.00390199 |  |  |
| 2020-09-01 |  |  |  | 0.00780234 |
| 2021-02-01 |  | 0.00389818 |  |  |
| 2021-07-01 |  | 0.00389033 |  |  |
| 2021-08-01 |  | 0.00389033 |  |  |
| 2022-03-01 |  |  | 0.00385907 |  |

Indometacin

|  | Indometacin capsule 25 mg 05126T | Indometacin suppository 100 mg 05128X |
| --- | --- | --- |
| 2014-02-01 | 0.0427237 | 0.0170895 |
| 2014-03-01 | 0.0640856 | 0.00427237 |
| 2014-04-01 | 0.102233 | 0.0127792 |
| 2014-05-01 | 0.0553764 | 0.00425973 |
| 2014-06-01 | 0.0553764 |  |
| 2014-07-01 | 0.0509275 | 0.0127319 |
| 2014-08-01 | 0.0551715 |  |
| 2014-09-01 | 0.0424396 | 0.00424396 |
| 2014-10-01 | 0.0338405 | 0.00423006 |
| 2014-11-01 | 0.0592208 | 0.00846012 |
| 2014-12-01 | 0.0380705 | 0.0169202 |
| 2015-01-01 | 0.0589582 | 0.0126339 |
| 2015-02-01 | 0.0294791 | 0.0042113 |
| 2015-03-01 | 0.0379017 | 0.0126339 |
| 2015-04-01 | 0.0713806 | 0.0167954 |
| 2015-05-01 | 0.0419886 | 0.00419886 |
| 2015-06-01 | 0.0461874 |  |
| 2015-07-01 | 0.0292835 | 0.00836671 |
| 2015-08-01 | 0.0292835 | 0.00418335 |
| 2015-09-01 | 0.0376502 | 0.00836671 |
| 2015-10-01 | 0.0250161 | 0.00416935 |
| 2015-11-01 | 0.0208467 | 0.00416935 |
| 2015-12-01 | 0.0416935 | 0.012508 |
| 2016-01-01 | 0.0414879 | 0.00414879 |
| 2016-02-01 | 0.078827 | 0.0124464 |
| 2016-03-01 | 0.0414879 |  |
| 2016-04-01 | 0.0248027 |  |
| 2016-05-01 | 0.0372041 | 0.00413378 |
| 2016-06-01 | 0.0289365 | 0.00826757 |
| 2016-07-01 | 0.069966 |  |
| 2016-08-01 | 0.0329252 |  |
| 2016-09-01 | 0.00823129 | 0.00823129 |
| 2016-10-01 | 0.032807 | 0.00820174 |
| 2016-11-01 | 0.0205044 | 0.00820174 |
| 2016-12-01 | 0.0410087 | 0.00820174 |
| 2017-01-01 | 0.0163189 | 0.00815947 |
| 2017-02-01 | 0.0163189 |  |
| 2017-03-01 | 0.0367176 | 0.00815947 |
| 2017-04-01 | 0.0487952 | 0.00406627 |
| 2017-05-01 | 0.0894578 | 0.00406627 |
| 2017-06-01 | 0.0406627 |  |
| 2017-07-01 | 0.076954 |  |
| 2017-08-01 | 0.0486025 | 0.00405021 |
| 2017-09-01 | 0.0486025 | 0.00405021 |
| 2017-10-01 | 0.0444283 |  |
| 2017-11-01 | 0.0525061 | 0.00403893 |
| 2017-12-01 | 0.0646229 | 0.00403893 |
| 2018-01-01 | 0.0241141 | 0.00401901 |
| 2018-02-01 | 0.0562661 |  |
| 2018-03-01 | 0.0281331 | 0.00401901 |
| 2018-04-01 | 0.0320471 |  |
| 2018-05-01 | 0.0520765 | 0.00400589 |
| 2018-06-01 | 0.036053 | 0.00801177 |
| 2018-07-01 | 0.0438817 | 0.00797849 |
| 2018-08-01 | 0.0438817 | 0.00398925 |
| 2018-09-01 | 0.0279247 |  |
| 2018-10-01 | 0.047721 | 0.00397675 |
| 2018-11-01 | 0.0516978 | 0.00795351 |
| 2018-12-01 | 0.047721 |  |
| 2019-01-01 | 0.0197903 |  |
| 2019-02-01 | 0.0197903 |  |
| 2019-03-01 | 0.0237483 | 0.00395806 |
| 2019-04-01 | 0.02763 | 0.00394714 |
| 2019-05-01 | 0.02763 |  |
| 2019-06-01 | 0.0315771 |  |
| 2019-07-01 | 0.0235867 | 0.00393112 |
| 2019-08-01 | 0.03538 | 0.00393112 |
| 2019-09-01 | 0.0235867 |  |
| 2019-10-01 | 0.0313474 | 0.00783685 |
| 2019-11-01 | 0.0431027 |  |
| 2019-12-01 | 0.0626948 | 0.0156737 |
| 2020-01-01 | 0.0390199 |  |
| 2020-02-01 | 0.0429219 |  |
| 2020-03-01 | 0.0507259 |  |
| 2020-04-01 | 0.0350887 | 0.0077975 |
| 2020-05-01 | 0.03119 | 0.0077975 |
| 2020-06-01 | 0.0272912 |  |
| 2020-07-01 | 0.0312094 | 0.00780234 |
| 2020-08-01 | 0.0390117 | 0.00390117 |
| 2020-09-01 | 0.023407 |  |
| 2020-10-01 | 0.0351141 | 0.00390157 |
| 2020-11-01 | 0.027311 | 0.00780314 |
| 2020-12-01 | 0.0312126 | 0.0156063 |
| 2021-01-01 | 0.0116945 | 0.00779636 |
| 2021-02-01 | 0.00389818 |  |
| 2021-03-01 | 0.0233891 |  |
| 2021-04-01 | 0.00778652 | 0.00389326 |
| 2021-05-01 | 0.015573 | 0.00389326 |
| 2021-06-01 | 0.0194663 | 0.00389326 |
| 2021-07-01 | 0.0311226 | 0.011671 |
| 2021-08-01 | 0.0194516 | 0.00389033 |
| 2021-09-01 | 0.011671 | 0.00389033 |
| 2021-10-01 | 0.0193995 | 0.00387991 |
| 2021-11-01 | 0.00387991 | 0.00387991 |
| 2021-12-01 | 0.0271594 | 0.00775982 |
| 2022-01-01 | 0.0231544 |  |
| 2022-02-01 | 0.0231544 | 0.0154363 |
| 2022-03-01 | 0.0231544 |  |
| 2022-04-01 | 0.0115302 | 0.00384339 |
| 2022-05-01 | 0.0192169 | 0.00768677 |
| 2022-06-01 | 0.0192169 |  |
| 2022-07-01 | 0.0191063 | 0.00764254 |
| 2022-08-01 | 0.0114638 | 0.00382127 |
| 2022-09-01 | 0.00382127 | 0.00382127 |
| 2022-10-01 | 0.0075987 |  |
| 2022-11-01 | 0.0189968 |  |
| 2022-12-01 | 0.0075987 |  |
| 2023-01-01 | 0.0150862 |  |
| 2023-02-01 | 0.00377155 | 0.00377155 |
| 2023-03-01 | 0.0188577 | 0.0075431 |
| 2023-04-01 | 0.0225057 |  |
| 2023-05-01 | 0.0075019 | 0.0112528 |
| 2023-06-01 | 0.0112528 | 0.0112528 |
| 2023-07-01 | 0.00372701 | 0.00372701 |
| 2023-08-01 | 0.00745403 |  |
| 2023-09-01 | 0.00372701 | 0.011181 |
| 2023-10-01 | 0.00741932 | 0.00370966 |
| 2023-11-01 | 0.0185483 |  |
| 2023-12-01 | 0.011129 |  |
| 2024-01-01 | 0.018441 | 0.0036882 |
| 2024-02-01 | 0.0110646 | 0.00737639 |
| 2024-03-01 | 0.00737639 |  |
| 2024-04-01 | 0.0110319 |  |
| 2024-05-01 | 0.0110319 | 0.00367731 |
| 2024-06-01 | 0.00367731 |  |
| 2024-07-01 | 0.0109876 | 0.00732507 |
| 2024-08-01 | 0.0146501 | 0.00366253 |
| 2024-09-01 | 0.0146501 |  |
| 2024-10-01 | 0.00365062 |  |
| 2024-11-01 | 0.0146025 |  |
| 2024-12-01 | 0.00730123 |  |
| 2025-01-01 | 0.00726299 | 0.00726299 |
| 2025-02-01 | 0.029052 |  |
| 2025-03-01 | 0.0108945 |  |
| 2025-04-01 | 0.0254205 |  |
| 2025-05-01 | 0.021789 | 0.00363149 |
| 2025-06-01 | 0.0181575 | 0.00363149 |
| 2025-07-01 | 0.0108945 |  |
| 2025-08-01 | 0.021789 |  |

Ketoprofen

|  | Ketoprofen capsule 200 mg (sustained release) 05136H |
| --- | --- |
| 2014-02-01 | 0.0811751 |
| 2014-03-01 | 0.0854475 |
| 2014-04-01 | 0.0638959 |
| 2014-05-01 | 0.0894543 |
| 2014-06-01 | 0.0766751 |
| 2014-07-01 | 0.0891232 |
| 2014-08-01 | 0.101855 |
| 2014-09-01 | 0.106099 |
| 2014-10-01 | 0.126902 |
| 2014-11-01 | 0.0507607 |
| 2014-12-01 | 0.0676809 |
| 2015-01-01 | 0.0589582 |
| 2015-02-01 | 0.0715921 |
| 2015-03-01 | 0.0547469 |
| 2015-04-01 | 0.0755795 |
| 2015-05-01 | 0.0629829 |
| 2015-06-01 | 0.125966 |
| 2015-07-01 | 0.196618 |
| 2015-08-01 | 0.104584 |
| 2015-09-01 | 0.104584 |
| 2015-10-01 | 0.137588 |
| 2015-11-01 | 0.0833869 |
| 2015-12-01 | 0.0583708 |
| 2016-01-01 | 0.0248927 |
| 2016-02-01 | 0.0871245 |
| 2016-03-01 | 0.0331903 |
| 2016-04-01 | 0.0785419 |
| 2016-05-01 | 0.057873 |
| 2016-06-01 | 0.0702743 |
| 2016-07-01 | 0.0493877 |
| 2016-08-01 | 0.0246939 |
| 2016-09-01 | 0.0493877 |
| 2016-10-01 | 0.0820174 |
| 2016-11-01 | 0.0738157 |
| 2016-12-01 | 0.0615131 |
| 2017-01-01 | 0.0571163 |
| 2017-02-01 | 0.0775149 |
| 2017-03-01 | 0.0203987 |
| 2017-04-01 | 0.0406627 |
| 2017-05-01 | 0.0365964 |
| 2017-06-01 | 0.0813253 |
| 2017-07-01 | 0.0648034 |
| 2017-08-01 | 0.0486025 |
| 2017-09-01 | 0.0729038 |
| 2017-10-01 | 0.0807786 |
| 2017-11-01 | 0.0767397 |
| 2017-12-01 | 0.0888565 |
| 2018-01-01 | 0.0522471 |
| 2018-02-01 | 0.0763612 |
| 2018-03-01 | 0.0602851 |
| 2018-04-01 | 0.0480706 |
| 2018-05-01 | 0.0881295 |
| 2018-06-01 | 0.0560824 |
| 2018-07-01 | 0.0438817 |
| 2018-08-01 | 0.0837741 |
| 2018-09-01 | 0.0518602 |
| 2018-10-01 | 0.0596513 |
| 2018-11-01 | 0.0596513 |
| 2018-12-01 | 0.0596513 |
| 2019-01-01 | 0.0435386 |
| 2019-02-01 | 0.0514547 |
| 2019-03-01 | 0.0593708 |
| 2019-04-01 | 0.0473656 |
| 2019-05-01 | 0.0434185 |
| 2019-06-01 | 0.0552599 |
| 2019-07-01 | 0.0275178 |
| 2019-08-01 | 0.0314489 |
| 2019-09-01 | 0.0235867 |
| 2019-10-01 | 0.0391842 |
| 2019-11-01 | 0.0235105 |
| 2019-12-01 | 0.0235105 |
| 2020-01-01 | 0.0429219 |
| 2020-02-01 | 0.0351179 |
| 2020-03-01 | 0.031216 |
| 2020-04-01 | 0.0233925 |
| 2020-05-01 | 0.046785 |
| 2020-06-01 | 0.0584812 |
| 2020-07-01 | 0.0624187 |
| 2020-08-01 | 0.0585175 |
| 2020-09-01 | 0.046814 |
| 2020-10-01 | 0.054622 |
| 2020-11-01 | 0.0624251 |
| 2020-12-01 | 0.0390157 |
| 2021-01-01 | 0.0506763 |
| 2021-02-01 | 0.0467781 |
| 2021-03-01 | 0.0506763 |
| 2021-04-01 | 0.0506124 |
| 2021-05-01 | 0.0506124 |
| 2021-06-01 | 0.0389326 |
| 2021-07-01 | 0.0427936 |
| 2021-08-01 | 0.0466839 |
| 2021-09-01 | 0.0311226 |
| 2021-10-01 | 0.0620786 |
| 2021-11-01 | 0.0310393 |
| 2021-12-01 | 0.0310393 |
| 2022-01-01 | 0.0270135 |
| 2022-02-01 | 0.0424498 |
| 2022-03-01 | 0.0385907 |
| 2022-04-01 | 0.0345905 |
| 2022-05-01 | 0.0307471 |
| 2022-06-01 | 0.0384339 |
| 2022-07-01 | 0.0343914 |
| 2022-08-01 | 0.042034 |
| 2022-09-01 | 0.0726041 |
| 2022-10-01 | 0.0379935 |
| 2022-11-01 | 0.0683883 |
| 2022-12-01 | 0.0455922 |
| 2023-01-01 | 0.0490301 |
| 2023-02-01 | 0.0301724 |
| 2023-03-01 | 0.0490301 |
| 2023-04-01 | 0.0525133 |
| 2023-05-01 | 0.0562642 |
| 2023-06-01 | 0.0600152 |
| 2023-07-01 | 0.0521782 |
| 2023-08-01 | 0.0409972 |
| 2023-09-01 | 0.0372701 |
| 2023-10-01 | 0.0408062 |
| 2023-11-01 | 0.0445159 |
| 2023-12-01 | 0.0333869 |
| 2024-01-01 | 0.0331938 |
| 2024-02-01 | 0.0442584 |
| 2024-03-01 | 0.0405702 |
| 2024-04-01 | 0.0625143 |
| 2024-05-01 | 0.0294185 |
| 2024-06-01 | 0.0404504 |
| 2024-07-01 | 0.0366253 |
| 2024-08-01 | 0.047613 |
| 2024-09-01 | 0.047613 |
| 2024-10-01 | 0.0438074 |
| 2024-11-01 | 0.0292049 |
| 2024-12-01 | 0.0146025 |
| 2025-01-01 | 0.021789 |
| 2025-02-01 | 0.014526 |
| 2025-03-01 | 0.0108945 |

Lincomycin

|  | Lincomycin injection 600 mg in 2 mL 05144R | Lincomycin injection 600 mg in 2 mL 11366F |
| --- | --- | --- |
| 2014-02-01 | 0.00427237 |  |
| 2014-04-01 | 0.0127792 |  |
| 2014-05-01 | 0.00425973 |  |
| 2014-07-01 | 0.00848792 |  |
| 2014-12-01 | 0.00423006 |  |
| 2015-03-01 | 0.0042113 |  |
| 2015-08-01 | 0.00836671 |  |
| 2015-09-01 | 0.00836671 |  |
| 2015-10-01 | 0.00416935 |  |
| 2015-12-01 | 0.00416935 |  |
| 2016-04-01 | 0.00413378 |  |
| 2016-05-01 | 0.00413378 |  |
| 2016-07-01 | 0.00411564 |  |
| 2016-09-01 | 0.00411564 |  |
| 2016-12-01 | 0.00410087 |  |
| 2017-06-01 | 0.00406627 |  |
| 2017-12-01 | 0.00403893 |  |
| 2018-06-01 | 0.00400589 |  |
| 2018-07-01 | 0.00398925 |  |
| 2019-04-01 | 0.00394714 |  |
| 2019-08-01 |  | 0.00393112 |
| 2020-07-01 | 0.00780234 |  |
| 2021-10-01 | 0.0116397 |  |
| 2022-01-01 |  | 0.00385907 |
| 2022-05-01 |  | 0.00384339 |
| 2023-06-01 |  | 0.00375095 |
| 2023-08-01 | 0.00372701 |  |
| 2024-02-01 | 0.0036882 |  |
| 2024-06-01 | 0.00367731 |  |
| 2024-10-01 | 0.00365062 |  |

Methylprednisolone

|  | Methylprednisolone injection 40 mg in 1 mL 05148Y |
| --- | --- |
| 2014-02-01 | 0.00427237 |
| 2014-04-01 | 0.0127792 |
| 2014-05-01 | 0.00425973 |
| 2014-06-01 | 0.00851945 |
| 2014-07-01 | 0.0169758 |
| 2014-08-01 | 0.00424396 |
| 2014-09-01 | 0.00848792 |
| 2014-10-01 | 0.0211503 |
| 2014-11-01 | 0.00846012 |
| 2014-12-01 | 0.00423006 |
| 2015-02-01 | 0.0084226 |
| 2015-03-01 | 0.0084226 |
| 2015-04-01 | 0.00839772 |
| 2015-05-01 | 0.00419886 |
| 2015-07-01 | 0.00418335 |
| 2015-08-01 | 0.00836671 |
| 2015-11-01 | 0.00416935 |
| 2015-12-01 | 0.00416935 |
| 2016-04-01 | 0.0124014 |
| 2016-07-01 | 0.00411564 |
| 2016-08-01 | 0.0123469 |
| 2016-09-01 | 0.00411564 |
| 2016-10-01 | 0.00820174 |
| 2017-01-01 | 0.00407973 |
| 2017-04-01 | 0.00406627 |
| 2017-05-01 | 0.00406627 |
| 2017-09-01 | 0.00405021 |
| 2018-01-01 | 0.00401901 |
| 2018-02-01 | 0.00803802 |
| 2018-07-01 | 0.00398925 |
| 2018-08-01 | 0.00398925 |
| 2018-10-01 | 0.00397675 |
| 2019-01-01 | 0.00395806 |
| 2019-02-01 | 0.00395806 |
| 2019-03-01 | 0.00395806 |
| 2019-05-01 | 0.00394714 |
| 2019-08-01 | 0.00393112 |
| 2019-09-01 | 0.00786223 |
| 2019-10-01 | 0.00783685 |
| 2019-11-01 | 0.00391842 |
| 2020-01-01 | 0.00780399 |
| 2020-03-01 | 0.00390199 |
| 2020-04-01 | 0.00389875 |
| 2020-07-01 | 0.00390117 |
| 2020-09-01 | 0.0195058 |
| 2020-11-01 | 0.00780314 |
| 2021-02-01 | 0.00389818 |
| 2021-03-01 | 0.00779636 |
| 2021-05-01 | 0.00778652 |
| 2021-06-01 | 0.00778652 |
| 2021-07-01 | 0.00389033 |
| 2021-08-01 | 0.00778066 |
| 2021-09-01 | 0.00778066 |
| 2021-10-01 | 0.00387991 |
| 2021-12-01 | 0.0116397 |
| 2022-02-01 | 0.00385907 |
| 2022-04-01 | 0.00384339 |
| 2022-05-01 | 0.00768677 |
| 2022-08-01 | 0.00382127 |
| 2022-10-01 | 0.00379935 |
| 2022-12-01 | 0.0075987 |
| 2023-02-01 | 0.00377155 |
| 2023-03-01 | 0.00377155 |
| 2023-04-01 | 0.0075019 |
| 2023-05-01 | 0.00375095 |
| 2023-06-01 | 0.0075019 |
| 2023-08-01 | 0.00745403 |
| 2023-09-01 | 0.00372701 |
| 2023-10-01 | 0.00370966 |
| 2023-11-01 | 0.00370966 |
| 2023-12-01 | 0.00370966 |
| 2024-01-01 | 0.0036882 |
| 2024-02-01 | 0.00737639 |
| 2024-03-01 | 0.0036882 |
| 2024-04-01 | 0.00367731 |
| 2024-05-01 | 0.00367731 |
| 2024-06-01 | 0.00735462 |
| 2024-07-01 | 0.00366253 |
| 2024-08-01 | 0.00366253 |
| 2024-09-01 | 0.00366253 |
| 2024-10-01 | 0.00730123 |
| 2024-11-01 | 0.00365062 |
| 2024-12-01 | 0.00730123 |
| 2025-05-01 | 0.00363149 |
| 2025-06-01 | 0.00363149 |

Metoclopramide

|  | Metoclopramide injection 10 mg in 2 mL 05153F | Metoclopramide tablet 10 mg 05151D |
| --- | --- | --- |
| 2014-02-01 | 0.00427237 | 0.183712 |
| 2014-03-01 |  | 0.123899 |
| 2014-04-01 |  | 0.187428 |
| 2014-05-01 |  | 0.183168 |
| 2014-06-01 |  | 0.140571 |
| 2014-07-01 |  | 0.216442 |
| 2014-08-01 |  | 0.127319 |
| 2014-09-01 |  | 0.195222 |
| 2014-10-01 |  | 0.160742 |
| 2014-11-01 | 0.00423006 | 0.173432 |
| 2014-12-01 | 0.00846012 | 0.156512 |
| 2015-01-01 | 0.0042113 | 0.164241 |
| 2015-02-01 | 0.0042113 | 0.214776 |
| 2015-03-01 |  | 0.176875 |
| 2015-04-01 |  | 0.167954 |
| 2015-05-01 | 0.00419886 | 0.14696 |
| 2015-06-01 | 0.00419886 | 0.176352 |
| 2015-07-01 | 0.00418335 | 0.133867 |
| 2015-08-01 |  | 0.158967 |
| 2015-09-01 |  | 0.163151 |
| 2015-10-01 | 0.00416935 | 0.108403 |
| 2015-11-01 | 0.0166774 | 0.145927 |
| 2015-12-01 |  | 0.187621 |
| 2016-01-01 |  | 0.112017 |
| 2016-02-01 |  | 0.141059 |
| 2016-03-01 |  | 0.174249 |
| 2016-04-01 |  | 0.144682 |
| 2016-05-01 |  | 0.165351 |
| 2016-06-01 |  | 0.161218 |
| 2016-07-01 |  | 0.16051 |
| 2016-08-01 | 0.00411564 | 0.131701 |
| 2016-09-01 |  | 0.209898 |
| 2016-10-01 |  | 0.0779165 |
| 2016-11-01 | 0.00410087 | 0.135329 |
| 2016-12-01 |  | 0.155833 |
| 2017-01-01 |  | 0.126472 |
| 2017-02-01 |  | 0.118312 |
| 2017-03-01 |  | 0.114233 |
| 2017-04-01 |  | 0.121988 |
| 2017-05-01 | 0.00813253 | 0.109789 |
| 2017-06-01 | 0.00406627 | 0.0691265 |
| 2017-07-01 | 0.00405021 | 0.105305 |
| 2017-08-01 |  | 0.129607 |
| 2017-09-01 |  | 0.0972051 |
| 2017-10-01 |  | 0.100973 |
| 2017-11-01 | 0.00403893 | 0.11309 |
| 2017-12-01 |  | 0.0969344 |
| 2018-01-01 |  | 0.116551 |
| 2018-02-01 | 0.00803802 | 0.0602851 |
| 2018-03-01 | 0.00401901 | 0.100475 |
| 2018-04-01 |  | 0.144212 |
| 2018-05-01 |  | 0.0961413 |
| 2018-06-01 |  | 0.124183 |
| 2018-07-01 |  | 0.123667 |
| 2018-08-01 |  | 0.155581 |
| 2018-09-01 |  | 0.131645 |
| 2018-10-01 |  | 0.0954421 |
| 2018-11-01 |  | 0.0954421 |
| 2018-12-01 |  | 0.111349 |
| 2019-01-01 |  | 0.118742 |
| 2019-02-01 |  | 0.130616 |
| 2019-03-01 |  | 0.14249 |
| 2019-04-01 | 0.00394714 | 0.11052 |
| 2019-05-01 | 0.00394714 | 0.106573 |
| 2019-06-01 |  | 0.149991 |
| 2019-07-01 |  | 0.121865 |
| 2019-08-01 |  | 0.149382 |
| 2019-09-01 |  | 0.10614 |
| 2019-10-01 |  | 0.121471 |
| 2019-11-01 |  | 0.144982 |
| 2019-12-01 | 0.00391842 | 0.141063 |
| 2020-01-01 |  | 0.0624319 |
| 2020-02-01 |  | 0.0429219 |
| 2020-03-01 |  | 0.0624319 |
| 2020-04-01 |  | 0.0272912 |
| 2020-05-01 |  | 0.0194937 |
| 2020-06-01 |  | 0.0662787 |
| 2020-07-01 |  | 0.0429129 |
| 2020-08-01 |  | 0.0312094 |
| 2020-09-01 |  | 0.0429129 |
| 2020-10-01 |  | 0.0507204 |
| 2020-11-01 |  | 0.0585236 |
| 2020-12-01 |  | 0.0663267 |
| 2021-01-01 |  | 0.0350836 |
| 2021-02-01 | 0.00389818 | 0.0701672 |
| 2021-03-01 |  | 0.0389818 |
| 2021-04-01 |  | 0.0583989 |
| 2021-05-01 |  | 0.0311461 |
| 2021-06-01 |  | 0.0934383 |
| 2021-07-01 |  | 0.0739162 |
| 2021-08-01 |  | 0.0661356 |
| 2021-09-01 |  | 0.0583549 |
| 2021-10-01 |  | 0.085358 |
| 2021-11-01 |  | 0.0349192 |
| 2021-12-01 |  | 0.042679 |
| 2022-01-01 |  | 0.0347316 |
| 2022-02-01 |  | 0.0270135 |
| 2022-03-01 |  | 0.0385907 |
| 2022-04-01 |  | 0.0422773 |
| 2022-05-01 |  | 0.0538074 |
| 2022-06-01 |  | 0.069181 |
| 2022-07-01 |  | 0.0343914 |
| 2022-08-01 |  | 0.042034 |
| 2022-09-01 |  | 0.0152851 |
| 2022-10-01 | 0.00379935 | 0.0227961 |
| 2022-11-01 |  | 0.0569903 |
| 2022-12-01 |  | 0.0379935 |
| 2023-01-01 |  | 0.0603448 |
| 2023-02-01 |  | 0.0226293 |
| 2023-03-01 |  | 0.0339439 |
| 2023-04-01 |  | 0.0262566 |
| 2023-05-01 |  | 0.0637661 |
| 2023-06-01 |  | 0.0412604 |
| 2023-07-01 |  | 0.0372701 |
| 2023-08-01 |  | 0.0372701 |
| 2023-09-01 |  | 0.0409972 |
| 2023-10-01 |  | 0.0370966 |
| 2023-11-01 |  | 0.0370966 |
| 2023-12-01 |  | 0.0482256 |
| 2024-01-01 |  | 0.0258174 |
| 2024-02-01 |  | 0.0442584 |
| 2024-03-01 |  | 0.0258174 |
| 2024-04-01 |  | 0.0367731 |
| 2024-05-01 |  | 0.0404504 |
| 2024-06-01 |  | 0.0257412 |
| 2024-07-01 |  | 0.0366253 |
| 2024-08-01 |  | 0.0219752 |
| 2024-09-01 |  | 0.0439504 |
| 2024-10-01 |  | 0.0328555 |
| 2024-11-01 |  | 0.0365062 |
| 2024-12-01 |  | 0.0328555 |
| 2025-01-01 |  | 0.0326834 |
| 2025-02-01 |  | 0.0399464 |
| 2025-03-01 |  | 0.0399464 |
| 2025-04-01 |  | 0.0653669 |
| 2025-05-01 |  | 0.014526 |
| 2025-06-01 |  | 0.0435779 |
| 2025-07-01 |  | 0.0472094 |
| 2025-08-01 |  | 0.0363149 |

Morphine

|  | Morphine injection 10 mg in 1 mL 10863R | Morphine injection 20 mg in 1 mL 10858L | Morphine injection 10 mg in 1 mL 05168B | Morphine injection 15 mg in 1 mL 05169C | Morphine injection 30 mg in 1 mL 05170D | Morphine oral solution 10 mg per mL, 1 mL 05239R | Morphine oral solution 2 mg per mL, 1 mL 05237P | Morphine oral solution 5 mg per mL, 1 mL 05238Q | Morphine tablet 30 mg 05163R |
| --- | --- | --- | --- | --- | --- | --- | --- | --- | --- |
| 2014-02-01 |  |  |  |  |  |  |  |  | 0.00427237 |
| 2014-05-01 |  |  |  |  | 0.00425973 |  |  |  |  |
| 2014-07-01 |  |  | 0.00424396 |  |  |  |  |  |  |
| 2014-08-01 |  |  |  |  |  |  | 0.00424396 |  |  |
| 2014-10-01 |  |  |  |  |  |  | 0.00423006 |  |  |
| 2015-01-01 |  |  |  |  |  |  | 0.0042113 |  |  |
| 2015-02-01 |  |  |  |  |  |  | 0.0042113 |  |  |
| 2015-04-01 |  |  |  |  |  |  | 0.00419886 | 0.00419886 |  |
| 2015-06-01 |  |  |  |  |  |  | 0.00839772 |  |  |
| 2015-07-01 |  |  |  |  |  | 0.00418335 |  |  | 0.00418335 |
| 2015-11-01 |  |  |  |  |  | 0.00416935 |  |  |  |
| 2015-12-01 |  |  | 0.00416935 |  |  |  | 0.00416935 | 0.00416935 |  |
| 2016-04-01 |  |  |  |  |  |  | 0.00413378 |  |  |
| 2016-06-01 |  |  | 0.00413378 |  |  |  |  |  |  |
| 2016-07-01 |  |  | 0.00411564 |  |  |  |  |  |  |
| 2016-10-01 |  |  |  |  |  | 0.00410087 |  |  |  |
| 2016-12-01 |  |  |  |  |  | 0.00410087 |  |  |  |
| 2017-01-01 |  |  |  |  |  | 0.00407973 |  |  |  |
| 2017-02-01 |  |  |  |  |  |  | 0.00407973 | 0.00407973 |  |
| 2017-03-01 |  |  |  |  |  |  |  | 0.00407973 |  |
| 2017-07-01 |  |  |  |  |  |  | 0.00810042 |  |  |
| 2017-09-01 |  |  |  |  |  | 0.00405021 |  |  |  |
| 2017-10-01 |  |  |  |  |  | 0.00403893 |  |  |  |
| 2017-11-01 |  |  |  |  | 0.00403893 | 0.00403893 |  | 0.00807786 |  |
| 2017-12-01 |  |  |  |  |  | 0.00807786 |  | 0.00403893 |  |
| 2018-01-01 |  |  |  |  |  |  | 0.00401901 |  |  |
| 2018-02-01 |  |  |  |  |  |  | 0.00803802 |  |  |
| 2018-12-01 |  |  |  |  |  |  | 0.00397675 |  |  |
| 2019-03-01 |  |  |  |  |  |  |  | 0.00395806 |  |
| 2019-06-01 |  |  |  |  |  |  |  |  | 0.00394714 |
| 2019-08-01 |  |  |  |  |  |  |  |  | 0.00393112 |
| 2020-02-01 |  |  |  |  |  |  | 0.00390199 |  |  |
| 2020-06-01 |  |  |  |  |  |  | 0.00389875 |  |  |
| 2020-11-01 |  |  | 0.00780314 |  |  |  |  |  |  |
| 2021-03-01 |  |  |  |  |  | 0.00389818 |  | 0.00389818 |  |
| 2021-04-01 |  |  |  |  |  |  | 0.00389326 |  |  |
| 2021-05-01 |  |  |  |  | 0.00389326 | 0.00389326 |  |  |  |
| 2021-06-01 |  |  | 0.00389326 |  |  | 0.00389326 |  |  |  |
| 2021-07-01 |  |  |  |  |  | 0.00389033 | 0.00778066 | 0.00389033 |  |
| 2021-09-01 |  |  |  |  |  |  | 0.00389033 |  |  |
| 2022-07-01 | 0.00382127 |  |  |  |  |  |  |  |  |
| 2022-08-01 |  |  |  |  |  |  | 0.00382127 |  |  |
| 2022-11-01 |  | 0.00379935 |  |  |  |  |  |  |  |
| 2023-07-01 |  |  |  | 0.00372701 |  |  |  |  |  |
| 2024-04-01 |  |  |  |  |  |  |  | 0.00367731 |  |
| 2025-07-01 |  |  |  |  |  |  |  | 0.00363149 |  |

Naloxone

|  | Naloxone injection 400 micrograms in 1 mL ampoule 10787R | Naloxone nasal spray 1.8 mg in 0.1 mL single dose unit, 2 11816X |
| --- | --- | --- |
| 2018-05-01 | 0.00400589 |  |
| 2020-01-01 | 0.00390199 |  |
| 2022-07-01 |  | 0.00382127 |
| 2022-08-01 | 0.00382127 |  |
| 2023-11-01 |  | 0.00370966 |
| 2024-03-01 |  | 0.0036882 |
| 2024-10-01 |  | 0.00365062 |

Naproxen

|  | Naproxen tablet 1 g (sustained release) 05179N | Naproxen tablet 250 mg 05176K | Naproxen tablet 500 mg 05177L | Naproxen tablet 750 mg (sustained release) 05178M | Naproxen tablet 550 mg 05186Y |
| --- | --- | --- | --- | --- | --- |
| 2014-02-01 | 0.0769027 | 0.128171 | 0.299066 | 0.0128171 | 0.183712 |
| 2014-03-01 | 0.0811751 | 0.102537 | 0.286249 | 0.00427237 | 0.17944 |
| 2014-04-01 | 0.0979737 | 0.110753 | 0.251324 | 0.0127792 | 0.191688 |
| 2014-05-01 | 0.0724153 | 0.174649 | 0.323739 | 0.0127792 | 0.195947 |
| 2014-06-01 | 0.123532 | 0.106493 | 0.366336 | 0.00851945 | 0.170389 |
| 2014-07-01 | 0.106099 | 0.0848792 | 0.356493 | 0.00848792 | 0.220686 |
| 2014-08-01 | 0.148539 | 0.123075 | 0.364981 | 0.0169758 | 0.18249 |
| 2014-09-01 | 0.106099 | 0.110343 | 0.42864 | 0.0127319 | 0.186734 |
| 2014-10-01 | 0.126902 | 0.0972914 | 0.520297 | 0.0423006 | 0.194583 |
| 2014-11-01 | 0.105751 | 0.114212 | 0.482227 | 0.0126902 | 0.207273 |
| 2014-12-01 | 0.0803711 | 0.109982 | 0.490687 | 0.0296104 | 0.160742 |
| 2015-01-01 | 0.0926486 | 0.122128 | 0.357961 | 0.0463243 | 0.160029 |
| 2015-02-01 | 0.126339 | 0.113705 | 0.362172 | 0.0210565 | 0.197931 |
| 2015-03-01 | 0.0800147 | 0.151607 | 0.429553 | 0.042113 | 0.164241 |
| 2015-04-01 | 0.104971 | 0.100773 | 0.419886 | 0.0377897 | 0.193148 |
| 2015-05-01 | 0.088176 | 0.117568 | 0.424085 | 0.0125966 | 0.18475 |
| 2015-06-01 | 0.0461874 | 0.151159 | 0.390494 | 0.0167954 | 0.155358 |
| 2015-07-01 | 0.0627503 | 0.158967 | 0.439252 | 0.0251001 | 0.192434 |
| 2015-08-01 | 0.117134 | 0.167334 | 0.405785 | 0.0167334 | 0.196618 |
| 2015-09-01 | 0.0920338 | 0.158967 | 0.313751 | 0.0418335 | 0.200801 |
| 2015-10-01 | 0.0792176 | 0.145927 | 0.387749 | 0.0291854 | 0.212637 |
| 2015-11-01 | 0.12508 | 0.12508 | 0.504491 | 0.0416935 | 0.170943 |
| 2015-12-01 | 0.133419 | 0.0875563 | 0.391918 | 0.0375241 | 0.179282 |
| 2016-01-01 | 0.0746782 | 0.0954221 | 0.37754 | 0.0248927 | 0.116166 |
| 2016-02-01 | 0.0705294 | 0.0912733 | 0.435623 | 0.0497855 | 0.153505 |
| 2016-03-01 | 0.0912733 | 0.10372 | 0.323605 | 0.0373391 | 0.165952 |
| 2016-04-01 | 0.132281 | 0.0785419 | 0.318301 | 0.0413378 | 0.095077 |
| 2016-05-01 | 0.111612 | 0.0702743 | 0.438181 | 0.057873 | 0.173619 |
| 2016-06-01 | 0.181887 | 0.0992108 | 0.49192 | 0.0785419 | 0.169485 |
| 2016-07-01 | 0.156394 | 0.0781972 | 0.374524 | 0.0617347 | 0.185204 |
| 2016-08-01 | 0.127585 | 0.111122 | 0.460952 | 0.107007 | 0.218129 |
| 2016-09-01 | 0.115238 | 0.127585 | 0.390986 | 0.0370408 | 0.152279 |
| 2016-10-01 | 0.106623 | 0.114824 | 0.446995 | 0.0615131 | 0.196842 |
| 2016-11-01 | 0.0738157 | 0.0984209 | 0.520811 | 0.032807 | 0.205044 |
| 2016-12-01 | 0.110724 | 0.0861183 | 0.451096 | 0.0205044 | 0.131228 |
| 2017-01-01 | 0.110153 | 0.0775149 | 0.448771 | 0.0285581 | 0.118312 |
| 2017-02-01 | 0.0775149 | 0.106073 | 0.432452 | 0.00407973 | 0.203987 |
| 2017-03-01 | 0.101993 | 0.0815947 | 0.318219 | 0.0326379 | 0.142791 |
| 2017-04-01 | 0.0731928 | 0.0894578 | 0.398494 |  | 0.121988 |
| 2017-05-01 | 0.178916 | 0.0853916 | 0.443223 | 0.0203313 | 0.130121 |
| 2017-06-01 | 0.158584 | 0.134187 | 0.398494 | 0.0203313 | 0.146386 |
| 2017-07-01 | 0.129607 | 0.109356 | 0.522477 | 0.0445523 | 0.210611 |
| 2017-08-01 | 0.121506 | 0.109356 | 0.518427 | 0.0162008 | 0.149858 |
| 2017-09-01 | 0.117456 | 0.0810042 | 0.546779 | 0.0162008 | 0.141757 |
| 2017-10-01 | 0.0686619 | 0.125207 | 0.5291 | 0.0282725 | 0.133285 |
| 2017-11-01 | 0.137324 | 0.109051 | 0.549295 | 0.00807786 | 0.161557 |
| 2017-12-01 | 0.100973 | 0.109051 | 0.488711 | 0.0282725 | 0.125207 |
| 2018-01-01 | 0.0964562 | 0.112532 | 0.426015 | 0.016076 | 0.100475 |
| 2018-02-01 | 0.152722 | 0.0964562 | 0.434053 | 0.0321521 | 0.116551 |
| 2018-03-01 | 0.12057 | 0.116551 | 0.458167 | 0.0241141 | 0.100475 |
| 2018-04-01 | 0.0921354 | 0.108159 | 0.388571 | 0.0160235 | 0.140206 |
| 2018-05-01 | 0.104153 | 0.172253 | 0.488718 | 0.0200294 | 0.140206 |
| 2018-06-01 | 0.100147 | 0.108159 | 0.364536 | 0.0200294 | 0.172253 |
| 2018-07-01 | 0.0997311 | 0.151591 | 0.398925 | 0.0199462 | 0.191484 |
| 2018-08-01 | 0.135634 | 0.195473 | 0.378978 | 0.0239355 | 0.155581 |
| 2018-09-01 | 0.127656 | 0.0957419 | 0.434828 | 0.0478709 | 0.163559 |
| 2018-10-01 | 0.107372 | 0.194861 | 0.433466 | 0.031814 | 0.171 |
| 2018-11-01 | 0.0715816 | 0.163047 | 0.425513 | 0.0278373 | 0.194861 |
| 2018-12-01 | 0.0795351 | 0.163047 | 0.469257 | 0.0357908 | 0.115326 |
| 2019-01-01 | 0.0910353 | 0.102909 | 0.26519 | 0.0395806 | 0.126658 |
| 2019-02-01 | 0.189987 | 0.114784 | 0.26519 | 0.0158322 | 0.126658 |
| 2019-03-01 | 0.178112 | 0.110826 | 0.423512 | 0.0277064 | 0.229567 |
| 2019-04-01 | 0.169727 | 0.118414 | 0.347348 | 0.0394714 | 0.161833 |
| 2019-05-01 | 0.169727 | 0.205251 | 0.410502 | 0.00789427 | 0.126308 |
| 2019-06-01 | 0.134203 | 0.146044 | 0.24867 | 0.0355242 | 0.142097 |
| 2019-07-01 | 0.165107 | 0.129727 | 0.393112 | 0.0432423 | 0.14152 |
| 2019-08-01 | 0.14152 | 0.149382 | 0.349869 | 0.0196556 | 0.161176 |
| 2019-09-01 | 0.161176 | 0.149382 | 0.38918 | 0.0471734 | 0.169038 |
| 2019-10-01 | 0.164574 | 0.160655 | 0.442782 | 0.027429 | 0.12539 |
| 2019-11-01 | 0.137145 | 0.133226 | 0.49764 | 0.0352658 | 0.156737 |
| 2019-12-01 | 0.12539 | 0.117553 | 0.391842 | 0.0352658 | 0.121471 |
| 2020-01-01 | 0.132668 | 0.179492 | 0.378493 | 0.0468239 | 0.124864 |
| 2020-02-01 | 0.148276 | 0.15608 | 0.409709 | 0.0741379 | 0.140472 |
| 2020-03-01 | 0.167786 | 0.120962 | 0.378493 | 0.0429219 | 0.1951 |
| 2020-04-01 | 0.06238 | 0.0545825 | 0.37428 | 0.0116962 | 0.132557 |
| 2020-05-01 | 0.210532 | 0.09357 | 0.428862 | 0.0233925 | 0.179342 |
| 2020-06-01 | 0.257317 | 0.163747 | 0.506837 | 0.0545825 | 0.132557 |
| 2020-07-01 | 0.257477 | 0.13264 | 0.526658 | 0.0780234 | 0.222367 |
| 2020-08-01 | 0.206762 | 0.140442 | 0.514954 | 0.0663199 | 0.249675 |
| 2020-09-01 | 0.195058 | 0.195058 | 0.522757 | 0.0741222 | 0.292588 |
| 2020-10-01 | 0.19898 | 0.167768 | 0.526712 | 0.0936377 | 0.308224 |
| 2020-11-01 | 0.191177 | 0.187275 | 0.526712 | 0.117047 | 0.257504 |
| 2020-12-01 | 0.156063 | 0.0507204 | 0.351141 | 0.0897361 | 0.308224 |
| 2021-01-01 | 0.132538 | 0.0779636 | 0.272873 | 0.101353 | 0.136436 |
| 2021-02-01 | 0.183214 | 0.0740654 | 0.366429 | 0.155927 | 0.218298 |
| 2021-03-01 | 0.253382 | 0.109149 | 0.409309 | 0.167622 | 0.268974 |
| 2021-04-01 | 0.210236 | 0.101225 | 0.307568 | 0.0856517 | 0.260848 |
| 2021-05-01 | 0.163517 | 0.101225 | 0.373753 | 0.17909 | 0.233596 |
| 2021-06-01 | 0.175197 | 0.105118 | 0.0389326 | 0.0817585 | 0.225809 |
| 2021-07-01 | 0.245091 | 0.0894775 | 0.011671 | 0.12449 | 0.283994 |
| 2021-08-01 | 0.167284 | 0.1206 | 0.00389033 | 0.132271 | 0.35402 |
| 2021-09-01 | 0.171174 | 0.0427936 |  | 0.0972582 | 0.400704 |
| 2021-10-01 | 0.225035 | 0.0387991 |  | 0.116397 | 0.333672 |
| 2021-11-01 | 0.170716 | 0.0504388 |  | 0.112517 | 0.318153 |
| 2021-12-01 | 0.143557 | 0.0737183 |  | 0.131917 | 0.240554 |
| 2022-01-01 | 0.150504 | 0.054027 |  | 0.127349 | 0.177517 |
| 2022-02-01 | 0.135067 | 0.0578861 |  | 0.135067 | 0.219967 |
| 2022-03-01 | 0.127349 | 0.111913 |  | 0.115772 | 0.239262 |
| 2022-04-01 | 0.134519 | 0.049964 |  | 0.111458 | 0.199856 |
| 2022-05-01 | 0.21523 | 0.0614942 |  | 0.0845545 | 0.199856 |
| 2022-06-01 | 0.230603 | 0.0845545 |  | 0.111458 | 0.199856 |
| 2022-07-01 | 0.187242 | 0.0649616 |  | 0.0878892 | 0.21017 |
| 2022-08-01 | 0.233097 | 0.0917105 |  | 0.137566 | 0.229276 |
| 2022-09-01 | 0.313344 | 0.0687829 |  | 0.099353 | 0.183421 |
| 2022-10-01 | 0.224162 | 0.0835857 |  | 0.140576 | 0.265955 |
| 2022-11-01 | 0.246958 | 0.0721877 |  | 0.0987831 | 0.269754 |
| 2022-12-01 | 0.220362 | 0.136777 |  | 0.113981 | 0.216563 |
| 2023-01-01 | 0.165948 | 0.113146 |  | 0.0867456 | 0.158405 |
| 2023-02-01 | 0.211207 | 0.0678879 |  | 0.0905172 | 0.196121 |
| 2023-03-01 | 0.207435 | 0.150862 |  | 0.150862 | 0.188577 |
| 2023-04-01 | 0.217555 | 0.142536 |  | 0.0600152 | 0.105027 |
| 2023-05-01 | 0.307578 | 0.108778 |  | 0.0825209 | 0.187547 |
| 2023-06-01 | 0.292574 | 0.105027 |  | 0.071268 | 0.172544 |
| 2023-07-01 | 0.216167 | 0.223621 |  | 0.0670863 | 0.17517 |
| 2023-08-01 | 0.242256 | 0.454696 |  | 0.0745403 | 0.201259 |
| 2023-09-01 | 0.190078 | 0.316796 |  | 0.0670863 | 0.190078 |
| 2023-10-01 | 0.370966 | 0.255966 |  | 0.0704835 | 0.204031 |
| 2023-11-01 | 0.244837 | 0.263386 |  | 0.10387 | 0.159515 |
| 2023-12-01 | 0.133548 | 0.148386 |  | 0.0593545 | 0.133548 |
| 2024-01-01 | 0.158592 | 0.158592 |  | 0.0700757 | 0.121711 |
| 2024-02-01 | 0.228668 | 0.14384 |  | 0.121711 | 0.136463 |
| 2024-03-01 | 0.136463 | 0.169657 |  | 0.0811403 | 0.165969 |
| 2024-04-01 | 0.209607 | 0.139738 |  | 0.0698689 | 0.165479 |
| 2024-05-01 | 0.143415 | 0.158124 |  | 0.0882554 | 0.143415 |
| 2024-06-01 | 0.198575 | 0.165479 |  | 0.0956101 | 0.125029 |
| 2024-07-01 | 0.205102 | 0.142839 |  | 0.113539 | 0.153826 |
| 2024-08-01 | 0.179464 | 0.131851 |  | 0.106214 | 0.168477 |
| 2024-09-01 | 0.135514 | 0.179464 |  | 0.0915634 | 0.183127 |
| 2024-10-01 | 0.160627 | 0.208085 |  | 0.102217 | 0.240941 |
| 2024-11-01 | 0.17523 | 0.11682 |  | 0.0985666 | 0.189832 |
| 2024-12-01 | 0.113169 | 0.0839642 |  | 0.102217 | 0.11682 |
| 2025-01-01 | 0.112576 | 0.152523 |  | 0.119839 | 0.116208 |
| 2025-02-01 | 0.141628 | 0.130734 |  | 0.0944189 | 0.148891 |
| 2025-03-01 | 0.167049 | 0.148891 |  | 0.0980503 | 0.174312 |
| 2025-04-01 | 0.108945 | 0.17068 |  | 0.0980503 | 0.21789 |
| 2025-05-01 | 0.206995 | 0.127102 |  | 0.119839 | 0.225153 |
| 2025-06-01 | 0.148891 | 0.192469 |  | 0.108945 | 0.181575 |
| 2025-07-01 | 0.134365 | 0.192469 |  | 0.127102 | 0.214258 |
| 2025-08-01 | 0.174312 | 0.174312 |  | 0.152523 | 0.163417 |

Nitrazepam

|  | Nitrazepam tablet 5 mg 05189D |
| --- | --- |
| 2014-02-01 | 0.0512685 |
| 2014-03-01 | 0.0427237 |
| 2014-04-01 | 0.0340778 |
| 2014-05-01 | 0.0638959 |
| 2014-06-01 | 0.0425973 |
| 2014-07-01 | 0.0339517 |
| 2014-08-01 | 0.0339517 |
| 2014-09-01 | 0.0212198 |
| 2014-10-01 | 0.0296104 |
| 2014-11-01 | 0.0253804 |
| 2014-12-01 | 0.0338405 |
| 2015-01-01 | 0.0336904 |
| 2015-02-01 | 0.042113 |
| 2015-03-01 | 0.0379017 |
| 2015-04-01 | 0.0167954 |
| 2015-05-01 | 0.0377897 |
| 2015-06-01 | 0.029392 |
| 2015-07-01 | 0.0251001 |
| 2015-08-01 | 0.0292835 |
| 2015-09-01 | 0.0292835 |
| 2015-10-01 | 0.0333548 |
| 2015-11-01 | 0.0375241 |
| 2015-12-01 | 0.0250161 |
| 2016-01-01 | 0.0414879 |
| 2016-02-01 | 0.0290415 |
| 2016-03-01 | 0.0165952 |
| 2016-04-01 | 0.0206689 |
| 2016-05-01 | 0.0206689 |
| 2016-06-01 | 0.0124014 |
| 2016-07-01 | 0.0205782 |
| 2016-08-01 | 0.0288095 |
| 2016-09-01 | 0.0164626 |
| 2016-10-01 | 0.0410087 |
| 2016-11-01 | 0.032807 |
| 2016-12-01 | 0.0164035 |
| 2017-01-01 | 0.0203987 |
| 2017-02-01 | 0.0203987 |
| 2017-03-01 | 0.0285581 |
| 2017-04-01 | 0.0325301 |
| 2017-05-01 | 0.0243976 |
| 2017-06-01 | 0.0203313 |
| 2017-07-01 | 0.0283515 |
| 2017-08-01 | 0.0324017 |
| 2017-09-01 | 0.0324017 |
| 2017-10-01 | 0.0161557 |
| 2017-11-01 | 0.0323115 |
| 2017-12-01 | 0.0242336 |
| 2018-01-01 | 0.0241141 |
| 2018-02-01 | 0.0281331 |
| 2018-03-01 | 0.0241141 |
| 2018-04-01 | 0.0280412 |
| 2018-05-01 | 0.0160235 |
| 2018-06-01 | 0.036053 |
| 2018-07-01 | 0.0239355 |
| 2018-08-01 | 0.031914 |
| 2018-09-01 | 0.0279247 |
| 2018-10-01 | 0.031814 |
| 2018-11-01 | 0.0198838 |
| 2018-12-01 | 0.0278373 |
| 2019-01-01 | 0.0197903 |
| 2019-02-01 | 0.0197903 |
| 2019-03-01 | 0.0395806 |
| 2019-04-01 | 0.0315771 |
| 2019-05-01 | 0.0197357 |
| 2019-06-01 | 0.0355242 |
| 2019-07-01 | 0.0157245 |
| 2019-08-01 | 0.0314489 |
| 2019-09-01 | 0.0196556 |
| 2019-10-01 | 0.0195921 |
| 2019-11-01 | 0.0313474 |
| 2019-12-01 | 0.0195921 |
| 2020-01-01 | 0.01951 |
| 2020-02-01 | 0.023412 |
| 2020-03-01 | 0.01951 |
| 2020-04-01 | 0.0272912 |
| 2020-05-01 | 0.0194937 |
| 2020-06-01 | 0.0272912 |
| 2020-07-01 | 0.0195058 |
| 2020-08-01 | 0.0351105 |
| 2020-09-01 | 0.023407 |
| 2020-10-01 | 0.0156063 |
| 2020-11-01 | 0.0312126 |
| 2020-12-01 | 0.0156063 |
| 2021-01-01 | 0.0155927 |
| 2021-02-01 | 0.0116945 |
| 2021-03-01 | 0.0233891 |
| 2021-04-01 | 0.015573 |
| 2021-05-01 | 0.0233596 |
| 2021-06-01 | 0.0233596 |
| 2021-07-01 | 0.00778066 |
| 2021-08-01 | 0.0194516 |
| 2021-09-01 | 0.011671 |
| 2021-10-01 | 0.0271594 |
| 2021-11-01 | 0.0193995 |
| 2021-12-01 | 0.0310393 |
| 2022-01-01 | 0.0154363 |
| 2022-02-01 | 0.0154363 |
| 2022-03-01 | 0.0231544 |
| 2022-04-01 | 0.0115302 |
| 2022-05-01 | 0.0115302 |
| 2022-06-01 | 0.0115302 |
| 2022-07-01 | 0.0305702 |
| 2022-08-01 | 0.0152851 |
| 2022-09-01 | 0.0191063 |
| 2022-10-01 | 0.0113981 |
| 2022-11-01 | 0.0151974 |
| 2022-12-01 | 0.0151974 |
| 2023-01-01 | 0.0113146 |
| 2023-02-01 | 0.0188577 |
| 2023-03-01 | 0.0113146 |
| 2023-04-01 | 0.0150038 |
| 2023-05-01 | 0.0187547 |
| 2023-07-01 | 0.00745403 |
| 2023-08-01 | 0.011181 |
| 2023-09-01 | 0.00745403 |
| 2023-10-01 | 0.00370966 |
| 2023-11-01 | 0.011129 |
| 2023-12-01 | 0.011129 |
| 2024-01-01 | 0.00737639 |
| 2024-02-01 | 0.0036882 |
| 2024-03-01 | 0.0110646 |
| 2024-04-01 | 0.0147092 |
| 2024-05-01 | 0.00367731 |
| 2024-06-01 | 0.00735462 |
| 2024-07-01 | 0.0146501 |
| 2024-08-01 | 0.00732507 |
| 2024-09-01 | 0.00732507 |
| 2024-10-01 | 0.0109518 |
| 2024-11-01 | 0.00730123 |
| 2024-12-01 | 0.00730123 |
| 2025-01-01 | 0.00726299 |
| 2025-02-01 | 0.00726299 |
| 2025-03-01 | 0.00726299 |
| 2025-04-01 | 0.00363149 |
| 2025-05-01 | 0.00726299 |
| 2025-06-01 | 0.00363149 |
| 2025-07-01 | 0.0108945 |
| 2025-08-01 | 0.00726299 |

Nystatin

|  | Nystatin capsule 500,000 units 03345C | Nystatin oral suspension 100,000 units per mL, 24 mL 03343Y | Nystatin tablet 500,000 units 03342X |
| --- | --- | --- | --- |
| 2014-02-01 | 0.0256342 | 0.286249 | 0.0128171 |
| 2014-03-01 | 0.0170895 | 0.299066 | 0.0213619 |
| 2014-04-01 | 0.00851945 | 0.272622 | 0.0212986 |
| 2014-05-01 | 0.0127792 | 0.276882 | 0.0298181 |
| 2014-06-01 | 0.0212986 | 0.31096 | 0.00851945 |
| 2014-07-01 | 0.0297077 | 0.212198 | 0.0127319 |
| 2014-08-01 | 0.0254638 | 0.280101 | 0.0212198 |
| 2014-09-01 | 0.0212198 | 0.352249 | 0.0169758 |
| 2014-10-01 | 0.0126902 | 0.249573 | 0.0169202 |
| 2014-11-01 | 0.00846012 | 0.236883 | 0.0253804 |
| 2014-12-01 | 0.00846012 | 0.308794 | 0.0253804 |
| 2015-01-01 | 0.0210565 | 0.202142 | 0.0126339 |
| 2015-02-01 | 0.0084226 | 0.277946 | 0.0252678 |
| 2015-03-01 | 0.0294791 | 0.320059 | 0.0126339 |
| 2015-04-01 | 0.0125966 | 0.239335 | 0.0167954 |
| 2015-05-01 | 0.0167954 | 0.285522 | 0.00839772 |
| 2015-06-01 | 0.00839772 | 0.205744 | 0.0125966 |
| 2015-07-01 | 0.0125501 | 0.267735 | 0.0292835 |
| 2015-08-01 | 0.0167334 | 0.330485 | 0.0292835 |
| 2015-09-01 | 0.0125501 | 0.301201 | 0.0125501 |
| 2015-10-01 | 0.0208467 | 0.275177 | 0.012508 |
| 2015-11-01 | 0.012508 | 0.304362 | 0.0250161 |
| 2015-12-01 | 0.0166774 | 0.154266 | 0.0208467 |
| 2016-01-01 | 0.00829758 |  | 0.0248927 |
| 2016-02-01 | 0.0373391 |  | 0.0290415 |
| 2016-03-01 | 0.0165952 |  | 0.0165952 |
| 2016-04-01 | 0.0248027 |  | 0.0165351 |
| 2016-05-01 | 0.0289365 |  | 0.0289365 |
| 2016-06-01 | 0.00413378 |  | 0.0165351 |
| 2016-07-01 | 0.0164626 |  | 0.0164626 |
| 2016-08-01 | 0.0246939 |  | 0.0164626 |
| 2016-09-01 | 0.0164626 |  | 0.0205782 |
| 2016-10-01 | 0.0123026 |  | 0.0287061 |
| 2016-11-01 | 0.00820174 |  | 0.00410087 |
| 2016-12-01 | 0.032807 |  | 0.0246052 |
| 2017-01-01 | 0.0203987 |  | 0.00815947 |
| 2017-02-01 | 0.0203987 |  | 0.0122392 |
| 2017-03-01 | 0.0203987 |  | 0.0244784 |
| 2017-04-01 |  |  | 0.0243976 |
| 2017-05-01 | 0.0447289 |  | 0.0284639 |
| 2017-06-01 | 0.0325301 |  | 0.0243976 |
| 2017-07-01 | 0.0405021 |  | 0.0243013 |
| 2017-08-01 | 0.0243013 |  | 0.0202511 |
| 2017-09-01 | 0.0364519 |  | 0.0162008 |
| 2017-10-01 | 0.0282725 |  | 0.0282725 |
| 2017-11-01 | 0.0242336 |  | 0.0161557 |
| 2017-12-01 | 0.0242336 |  | 0.0121168 |
| 2018-01-01 | 0.0321521 |  | 0.00401901 |
| 2018-02-01 | 0.0241141 |  | 0.012057 |
| 2018-03-01 | 0.0321521 |  | 0.00803802 |
| 2018-04-01 | 0.0200294 |  | 0.0160235 |
| 2018-05-01 | 0.0480706 |  | 0.0120177 |
| 2018-06-01 | 0.0400589 |  | 0.00400589 |
| 2018-07-01 | 0.015957 |  | 0.00398925 |
| 2018-08-01 | 0.0518602 |  | 0.0239355 |
| 2018-09-01 | 0.015957 |  | 0.0119677 |
| 2018-10-01 | 0.047721 |  | 0.0119303 |
| 2018-11-01 | 0.0278373 |  | 0.00397675 |
| 2018-12-01 | 0.0278373 |  | 0.0119303 |
| 2019-01-01 | 0.0118742 |  | 0.00395806 |
| 2019-02-01 | 0.0237483 |  | 0.0277064 |
| 2019-03-01 | 0.0197903 |  | 0.0197903 |
| 2019-04-01 | 0.0118414 |  | 0.00789427 |
| 2019-05-01 | 0.0394714 |  | 0.00789427 |
| 2019-06-01 | 0.0236828 |  | 0.0118414 |
| 2019-07-01 | 0.0275178 |  | 0.0275178 |
| 2019-08-01 | 0.0825534 |  | 0.00786223 |
| 2019-09-01 | 0.0157245 |  | 0.0157245 |
| 2019-10-01 | 0.0313474 |  | 0.00391842 |
| 2019-11-01 | 0.027429 |  | 0.0195921 |
| 2019-12-01 | 0.0391842 |  | 0.00391842 |
| 2020-01-01 | 0.011706 |  | 0.00780399 |
| 2020-02-01 | 0.0429219 |  | 0.011706 |
| 2020-03-01 | 0.01951 |  | 0.023412 |
| 2020-04-01 | 0.0077975 |  | 0.0116962 |
| 2020-05-01 | 0.0116962 |  |  |
| 2020-06-01 | 0.00389875 |  | 0.00389875 |
| 2020-07-01 |  |  | 0.0117035 |
| 2020-08-01 | 0.00780234 |  |  |
| 2020-09-01 | 0.0273082 |  | 0.023407 |
| 2020-10-01 | 0.0117047 |  | 0.00390157 |
| 2020-11-01 | 0.0156063 |  | 0.0117047 |
| 2020-12-01 | 0.00390157 |  | 0.0195079 |
| 2021-01-01 | 0.00779636 |  |  |
| 2021-02-01 | 0.0155927 |  | 0.00779636 |
| 2021-03-01 | 0.0233891 |  | 0.00779636 |
| 2021-04-01 | 0.00778652 |  |  |
| 2021-05-01 | 0.0116798 |  | 0.00778652 |
| 2021-06-01 | 0.015573 |  |  |
| 2021-07-01 | 0.0155613 |  | 0.00778066 |
| 2021-08-01 | 0.00778066 |  | 0.00389033 |
| 2021-09-01 | 0.00389033 |  | 0.011671 |
| 2021-10-01 | 0.00387991 |  | 0.0193995 |
| 2021-11-01 | 0.00775982 |  | 0.0155196 |
| 2021-12-01 | 0.00775982 |  | 0.0116397 |
| 2022-01-01 | 0.0115772 |  | 0.00385907 |
| 2022-02-01 | 0.00771814 |  |  |
| 2022-03-01 | 0.00385907 |  | 0.0154363 |
| 2022-04-01 | 0.00768677 |  | 0.00768677 |
| 2022-05-01 | 0.00384339 |  | 0.0192169 |
| 2022-06-01 | 0.00768677 |  | 0.00768677 |
| 2022-07-01 |  |  | 0.00764254 |
| 2022-08-01 | 0.00764254 |  | 0.00382127 |
| 2022-09-01 | 0.0152851 |  |  |
| 2022-10-01 | 0.0113981 |  | 0.00379935 |
| 2022-11-01 | 0.00379935 |  | 0.00379935 |
| 2022-12-01 | 0.0151974 |  |  |
| 2023-01-01 | 0.0188577 |  | 0.0075431 |
| 2023-02-01 | 0.0226293 |  |  |
| 2023-03-01 | 0.0075431 |  | 0.0264008 |
| 2023-04-01 | 0.0337585 |  | 0.00375095 |
| 2023-05-01 | 0.0150038 |  | 0.0187547 |
| 2023-06-01 | 0.0187547 |  | 0.0112528 |
| 2023-07-01 | 0.0260891 |  |  |
| 2023-08-01 | 0.011181 |  |  |
| 2023-09-01 | 0.00745403 |  | 0.00745403 |
| 2023-10-01 | 0.011129 |  | 0.011129 |
| 2023-11-01 | 0.022258 |  | 0.011129 |
| 2023-12-01 | 0.0148386 |  |  |
| 2024-01-01 | 0.0147528 |  | 0.0110646 |
| 2024-02-01 | 0.00737639 |  | 0.0036882 |
| 2024-03-01 | 0.00737639 |  | 0.00737639 |
| 2024-04-01 | 0.0147092 |  | 0.0110319 |
| 2024-05-01 | 0.00367731 |  | 0.00367731 |
| 2024-06-01 |  |  | 0.0147092 |
| 2024-07-01 | 0.00732507 |  | 0.0183127 |
| 2024-08-01 | 0.0109876 |  | 0.00732507 |
| 2024-09-01 | 0.0219752 |  | 0.0183127 |
| 2024-10-01 | 0.0292049 |  | 0.00365062 |
| 2024-11-01 | 0.0146025 |  |  |
| 2024-12-01 | 0.00365062 |  |  |
| 2025-01-01 | 0.00363149 |  |  |
| 2025-02-01 | 0.00363149 |  | 0.00363149 |
| 2025-03-01 |  |  | 0.00363149 |
| 2025-04-01 | 0.00726299 |  | 0.021789 |
| 2025-05-01 | 0.0181575 |  | 0.00726299 |
| 2025-06-01 | 0.0181575 |  | 0.00726299 |
| 2025-07-01 | 0.00363149 |  | 0.00726299 |
| 2025-08-01 |  |  | 0.00726299 |

Oxazepam

|  | Oxazepam tablet 15 mg 05192G | Oxazepam tablet 30 mg 05193H |
| --- | --- | --- |
| 2014-02-01 | 0.0384514 | 0.106809 |
| 2014-03-01 | 0.0213619 | 0.115354 |
| 2014-04-01 | 0.0127792 | 0.102233 |
| 2014-05-01 | 0.0170389 | 0.16187 |
| 2014-06-01 | 0.0212986 | 0.093714 |
| 2014-07-01 | 0.0381956 | 0.106099 |
| 2014-08-01 | 0.0636594 | 0.0848792 |
| 2014-09-01 | 0.0381956 | 0.106099 |
| 2014-10-01 | 0.0296104 | 0.0803711 |
| 2014-11-01 | 0.0169202 | 0.0972914 |
| 2014-12-01 | 0.0380705 | 0.148052 |
| 2015-01-01 | 0.0168452 | 0.0800147 |
| 2015-02-01 | 0.0252678 | 0.117916 |
| 2015-03-01 | 0.0252678 | 0.113705 |
| 2015-04-01 | 0.058784 | 0.130165 |
| 2015-05-01 | 0.029392 | 0.0965738 |
| 2015-06-01 | 0.0125966 | 0.113369 |
| 2015-07-01 | 0.0502002 | 0.158967 |
| 2015-08-01 | 0.0292835 | 0.0962171 |
| 2015-09-01 | 0.0376502 | 0.1004 |
| 2015-10-01 | 0.00833869 | 0.0792176 |
| 2015-11-01 | 0.012508 | 0.0958949 |
| 2015-12-01 | 0.00416935 | 0.104234 |
| 2016-01-01 | 0.0124464 | 0.0871245 |
| 2016-02-01 | 0.00829758 | 0.0497855 |
| 2016-03-01 | 0.0248927 | 0.0622318 |
| 2016-04-01 | 0.0124014 | 0.0537392 |
| 2016-05-01 | 0.0248027 | 0.0785419 |
| 2016-06-01 | 0.0330703 | 0.0537392 |
| 2016-07-01 | 0.0205782 | 0.0864285 |
| 2016-08-01 | 0.0288095 | 0.119354 |
| 2016-09-01 | 0.0288095 | 0.0411564 |
| 2016-10-01 | 0.0205044 | 0.09432 |
| 2016-11-01 | 0.0164035 | 0.102522 |
| 2016-12-01 | 0.0287061 | 0.0820174 |
| 2017-01-01 | 0.0367176 | 0.061196 |
| 2017-02-01 | 0.0326379 | 0.061196 |
| 2017-03-01 | 0.00407973 | 0.0856744 |
| 2017-04-01 | 0.0243976 | 0.0528615 |
| 2017-05-01 | 0.0162651 | 0.0935241 |
| 2017-06-01 | 0.0243976 | 0.0813253 |
| 2017-07-01 | 0.0364519 | 0.0850544 |
| 2017-08-01 | 0.0364519 | 0.0688536 |
| 2017-09-01 | 0.0324017 | 0.0729038 |
| 2017-10-01 | 0.0242336 | 0.0969344 |
| 2017-11-01 | 0.0323115 | 0.0727008 |
| 2017-12-01 | 0.0323115 | 0.0848176 |
| 2018-01-01 | 0.012057 | 0.0964562 |
| 2018-02-01 | 0.020095 | 0.0884182 |
| 2018-03-01 | 0.020095 | 0.0763612 |
| 2018-04-01 | 0.0520765 | 0.0560824 |
| 2018-05-01 | 0.0320471 | 0.0921354 |
| 2018-06-01 | 0.036053 | 0.0640942 |
| 2018-07-01 | 0.0558494 | 0.10771 |
| 2018-08-01 | 0.0279247 | 0.0718064 |
| 2018-09-01 | 0.0279247 | 0.0877634 |
| 2018-10-01 | 0.0397675 | 0.0954421 |
| 2018-11-01 | 0.0676048 | 0.0715816 |
| 2018-12-01 | 0.0397675 | 0.0636281 |
| 2019-01-01 | 0.0118742 | 0.0514547 |
| 2019-02-01 | 0.0593708 | 0.0791611 |
| 2019-03-01 | 0.0197903 | 0.0752031 |
| 2019-04-01 | 0.0355242 | 0.0749956 |
| 2019-05-01 | 0.0315771 | 0.0631542 |
| 2019-06-01 | 0.02763 | 0.0671013 |
| 2019-07-01 | 0.0393112 | 0.0786223 |
| 2019-08-01 | 0.0275178 | 0.0589667 |
| 2019-09-01 | 0.0117933 | 0.0471734 |
| 2019-10-01 | 0.0313474 | 0.0666132 |
| 2019-11-01 | 0.0313474 | 0.0862053 |
| 2019-12-01 | 0.0391842 | 0.0940422 |
| 2020-01-01 | 0.0429219 | 0.0702359 |
| 2020-02-01 | 0.0507259 | 0.0936479 |
| 2020-03-01 | 0.023412 | 0.0936479 |
| 2020-04-01 | 0.0194937 | 0.0233925 |
| 2020-05-01 | 0.0506837 | 0.0584812 |
| 2020-06-01 | 0.046785 | 0.148152 |
| 2020-07-01 | 0.0390117 | 0.113134 |
| 2020-08-01 | 0.0936281 | 0.0936281 |
| 2020-09-01 | 0.0741222 | 0.156047 |
| 2020-10-01 | 0.0780314 | 0.0702283 |
| 2020-11-01 | 0.0585236 | 0.144358 |
| 2020-12-01 | 0.0312126 | 0.0702283 |
| 2021-01-01 | 0.0233891 | 0.0623709 |
| 2021-02-01 | 0.0311854 | 0.0623709 |
| 2021-03-01 | 0.0311854 | 0.0974545 |
| 2021-04-01 | 0.0350393 | 0.0622922 |
| 2021-05-01 | 0.0311461 | 0.0583989 |
| 2021-06-01 | 0.0311461 | 0.089545 |
| 2021-07-01 | 0.023342 | 0.11282 |
| 2021-08-01 | 0.0272323 | 0.0739162 |
| 2021-09-01 | 0.0505743 | 0.0972582 |
| 2021-10-01 | 0.0116397 | 0.0814781 |
| 2021-11-01 | 0.0271594 | 0.0775982 |
| 2021-12-01 | 0.0155196 | 0.0775982 |
| 2022-01-01 | 0.0308726 | 0.0463088 |
| 2022-02-01 | 0.0308726 | 0.0656042 |
| 2022-03-01 | 0.0154363 | 0.0733223 |
| 2022-04-01 | 0.0269037 | 0.069181 |
| 2022-05-01 | 0.0345905 | 0.069181 |
| 2022-06-01 | 0.0653376 | 0.0807111 |
| 2022-07-01 | 0.0343914 | 0.042034 |
| 2022-08-01 | 0.0267489 | 0.0878892 |
| 2022-09-01 | 0.0382127 | 0.0458552 |
| 2022-10-01 | 0.0493916 | 0.0873851 |
| 2022-11-01 | 0.0189968 | 0.0683883 |
| 2022-12-01 | 0.0151974 | 0.0721877 |
| 2023-01-01 | 0.0264008 | 0.075431 |
| 2023-02-01 | 0.0301724 | 0.075431 |
| 2023-03-01 | 0.0301724 | 0.0905172 |
| 2023-04-01 | 0.0375095 | 0.108778 |
| 2023-05-01 | 0.0412604 | 0.105027 |
| 2023-06-01 | 0.0450114 | 0.075019 |
| 2023-07-01 | 0.0633592 | 0.0521782 |
| 2023-08-01 | 0.0335431 | 0.0633592 |
| 2023-09-01 | 0.0372701 | 0.0782673 |
| 2023-10-01 | 0.0741932 | 0.0964511 |
| 2023-11-01 | 0.0445159 | 0.100161 |
| 2023-12-01 | 0.0445159 | 0.0556449 |
| 2024-01-01 | 0.0590112 | 0.0700757 |
| 2024-02-01 | 0.0405702 | 0.114334 |
| 2024-03-01 | 0.0626994 | 0.0737639 |
| 2024-04-01 | 0.0625143 | 0.0809008 |
| 2024-05-01 | 0.0404504 | 0.110319 |
| 2024-06-01 | 0.0404504 | 0.0514823 |
| 2024-07-01 | 0.0586006 | 0.0659256 |
| 2024-08-01 | 0.054938 | 0.0769132 |
| 2024-09-01 | 0.0329628 | 0.054938 |
| 2024-10-01 | 0.0584099 | 0.0620605 |
| 2024-11-01 | 0.0401568 | 0.109518 |
| 2024-12-01 | 0.0255543 | 0.047458 |
| 2025-01-01 | 0.029052 | 0.0508409 |
| 2025-02-01 | 0.0508409 | 0.0617354 |
| 2025-03-01 | 0.0544724 | 0.0544724 |
| 2025-04-01 | 0.0472094 | 0.0617354 |
| 2025-05-01 | 0.0435779 | 0.0544724 |
| 2025-06-01 | 0.0399464 | 0.0399464 |
| 2025-07-01 | 0.0472094 | 0.0762614 |
| 2025-08-01 | 0.0544724 | 0.0980503 |

Paracetamol

|  | Paracetamol oral liquid 120 mg per 5 mL, 100 mL 03348F | Paracetamol oral liquid 240 mg per 5 mL, 200 mL 03349G | Paracetamol tablet 500 mg 05196L | Paracetamol tablet 500 mg 05224Y |
| --- | --- | --- | --- | --- |
| 2014-02-01 | 0.00427237 | 0.0213619 | 0.16235 | 0.0213619 |
| 2014-03-01 | 0.00854475 | 0.00854475 | 0.153805 | 0.0256342 |
| 2014-04-01 |  | 0.00425973 | 0.123532 | 0.00851945 |
| 2014-05-01 |  | 0.0340778 | 0.212986 | 0.0383375 |
| 2014-06-01 | 0.00425973 | 0.0127792 | 0.119272 | 0.0255584 |
| 2014-07-01 | 0.00424396 |  | 0.0976111 | 0.00848792 |
| 2014-08-01 | 0.00848792 |  | 0.135807 | 0.0169758 |
| 2014-09-01 |  | 0.0127319 | 0.127319 | 0.0297077 |
| 2014-10-01 | 0.00423006 | 0.0169202 | 0.135362 | 0.0380705 |
| 2014-11-01 |  | 0.0296104 | 0.126902 |  |
| 2014-12-01 |  | 0.00423006 | 0.0972914 | 0.0169202 |
| 2015-01-01 | 0.0042113 | 0.0084226 | 0.0715921 | 0.0294791 |
| 2015-02-01 |  | 0.0126339 | 0.0800147 | 0.0294791 |
| 2015-03-01 |  | 0.0126339 | 0.13055 | 0.0084226 |
| 2015-04-01 | 0.00419886 | 0.0167954 | 0.125966 | 0.00839772 |
| 2015-05-01 | 0.0125966 | 0.0167954 | 0.113369 |  |
| 2015-06-01 | 0.00839772 | 0.0125966 | 0.138562 | 0.0167954 |
| 2015-07-01 |  | 0.0125501 | 0.163151 | 0.0167334 |
| 2015-08-01 |  | 0.00836671 | 0.167334 | 0.0209168 |
| 2015-09-01 |  | 0.00418335 | 0.171517 | 0.0167334 |
| 2015-10-01 |  | 0.00416935 | 0.187621 | 0.00416935 |
| 2015-11-01 |  | 0.0166774 | 0.262669 | 0.0375241 |
| 2015-12-01 |  | 0.0166774 | 0.208467 | 0.0333548 |
| 2016-01-01 | 0.00414879 | 0.00829758 | 0.124464 | 0.00829758 |
| 2016-02-01 | 0.00414879 | 0.00829758 | 0.174249 | 0.0165952 |
| 2016-03-01 |  | 0.0165952 | 0.161803 | 0.00414879 |
| 2016-04-01 | 0.00413378 |  | 0.18602 | 0.00826757 |
| 2016-05-01 | 0.00826757 | 0.00413378 | 0.173619 | 0.0165351 |
| 2016-06-01 |  | 0.0124014 | 0.206689 | 0.0248027 |
| 2016-07-01 |  | 0.00411564 | 0.222245 | 0.0246939 |
| 2016-08-01 |  | 0.0164626 | 0.16051 | 0.00823129 |
| 2016-09-01 |  | 0.00823129 | 0.181088 | 0.0123469 |
| 2016-10-01 |  | 0.00820174 | 0.114824 | 0.00820174 |
| 2016-11-01 |  | 0.0205044 | 0.192741 | 0.00410087 |
| 2016-12-01 | 0.00410087 | 0.00410087 | 0.13943 | 0.00820174 |
| 2017-01-01 |  | 0.0163189 | 0.118312 |  |
| 2017-02-01 |  | 0.0122392 | 0.14687 | 0.0163189 |
| 2017-03-01 |  | 0.00815947 | 0.110153 | 0.0122392 |
| 2017-04-01 |  | 0.00406627 | 0.0731928 | 0.00406627 |
| 2017-05-01 |  |  | 0.0935241 | 0.0162651 |
| 2017-06-01 |  | 0.00813253 | 0.191114 | 0.00813253 |
| 2017-07-01 |  | 0.0121506 | 0.18226 | 0.00405021 |
| 2017-08-01 | 0.00810042 | 0.00405021 | 0.243013 | 0.0324017 |
| 2017-09-01 |  | 0.0243013 | 0.174159 | 0.0121506 |
| 2017-10-01 | 0.00403893 |  | 0.250414 | 0.00807786 |
| 2017-11-01 |  | 0.00403893 | 0.145402 | 0.00807786 |
| 2017-12-01 |  | 0.00807786 | 0.0807786 | 0.0161557 |
| 2018-01-01 |  | 0.0241141 | 0.140665 |  |
| 2018-02-01 | 0.012057 | 0.012057 | 0.192912 | 0.012057 |
| 2018-03-01 |  | 0.00401901 | 0.184874 | 0.152722 |
| 2018-04-01 |  | 0.0240353 | 0.196288 | 0.0120177 |
| 2018-05-01 | 0.0120177 | 0.0160235 | 0.160235 | 0.0280412 |
| 2018-06-01 |  |  | 0.192283 | 0.0240353 |
| 2018-07-01 |  | 0.015957 | 0.231376 | 0.0279247 |
| 2018-08-01 | 0.00797849 | 0.015957 | 0.183505 | 0.0279247 |
| 2018-09-01 | 0.00398925 | 0.0119677 | 0.143613 | 0.0119677 |
| 2018-10-01 |  | 0.015907 | 0.163047 | 0.047721 |
| 2018-11-01 |  | 0.00795351 | 0.250535 | 0.0119303 |
| 2018-12-01 |  | 0.015907 | 0.222698 | 0.015907 |
| 2019-01-01 |  | 0.0118742 | 0.0910353 | 0.0197903 |
| 2019-02-01 |  | 0.0158322 | 0.197903 | 0.0118742 |
| 2019-03-01 |  | 0.0158322 | 0.189987 | 0.0277064 |
| 2019-04-01 |  | 0.00394714 | 0.13815 | 0.0157885 |
| 2019-05-01 | 0.00394714 | 0.00789427 | 0.0907841 | 0.00394714 |
| 2019-06-01 |  | 0.00789427 | 0.0907841 | 0.0394714 |
| 2019-07-01 | 0.00393112 |  | 0.153313 | 0.0117933 |
| 2019-08-01 |  | 0.00393112 | 0.110071 | 0.03538 |
| 2019-09-01 | 0.00393112 | 0.00393112 | 0.14152 |  |
| 2019-10-01 |  | 0.00783685 | 0.0979606 | 0.0117553 |
| 2019-11-01 |  |  | 0.0587764 | 0.0431027 |
| 2019-12-01 |  | 0.00783685 | 0.141063 | 0.027429 |
| 2020-01-01 |  | 0.00390199 | 0.132668 | 0.015608 |
| 2020-02-01 |  |  | 0.11706 | 0.011706 |
| 2020-03-01 |  | 0.015608 | 0.13657 | 0.00780399 |
| 2020-04-01 |  | 0.00389875 | 0.077975 |  |
| 2020-05-01 |  |  | 0.077975 | 0.0233925 |
| 2020-06-01 |  | 0.0077975 | 0.077975 | 0.0077975 |
| 2020-07-01 | 0.00390117 | 0.0117035 | 0.175553 | 0.0117035 |
| 2020-08-01 |  |  | 0.187256 | 0.00780234 |
| 2020-09-01 |  | 0.00390117 | 0.218465 | 0.0156047 |
| 2020-10-01 |  | 0.00390157 | 0.245799 | 0.00780314 |
| 2020-11-01 | 0.00390157 |  | 0.34724 | 0.00780314 |
| 2020-12-01 |  | 0.00390157 | 0.101441 | 0.027311 |
| 2021-01-01 |  | 0.00779636 | 0.132538 | 0.0155927 |
| 2021-02-01 | 0.00779636 |  | 0.194909 | 0.0116945 |
| 2021-03-01 |  | 0.00779636 | 0.202705 | 0.00779636 |
| 2021-04-01 |  | 0.00389326 | 0.128478 | 0.0233596 |
| 2021-05-01 |  | 0.00778652 | 0.140157 | 0.00778652 |
| 2021-06-01 | 0.00389326 | 0.0116798 | 0.0934383 | 0.0116798 |
| 2021-07-01 |  | 0.00778066 | 0.175065 | 0.00778066 |
| 2021-08-01 |  |  | 0.1206 | 0.00389033 |
| 2021-09-01 |  | 0.00389033 | 0.136161 | 0.00389033 |
| 2021-10-01 | 0.00775982 | 0.00387991 | 0.174596 | 0.00387991 |
| 2021-11-01 |  | 0.00775982 | 0.159076 | 0.0193995 |
| 2021-12-01 |  | 0.0116397 | 0.135797 | 0.00387991 |
| 2022-01-01 | 0.00385907 | 0.00771814 | 0.0964768 |  |
| 2022-02-01 |  | 0.00385907 | 0.0771814 | 0.0154363 |
| 2022-03-01 |  | 0.00771814 | 0.104195 | 0.00385907 |
| 2022-04-01 |  |  | 0.103771 | 0.00768677 |
| 2022-05-01 |  |  | 0.111458 | 0.00384339 |
| 2022-06-01 |  |  | 0.0845545 | 0.0192169 |
| 2022-07-01 |  | 0.00382127 | 0.0726041 |  |
| 2022-08-01 |  |  | 0.24074 | 0.0191063 |
| 2022-09-01 |  |  | 0.129923 | 0.0152851 |
| 2022-10-01 |  | 0.00379935 | 0.151974 | 0.0151974 |
| 2022-11-01 |  |  | 0.193767 | 0.0265955 |
| 2022-12-01 |  |  | 0.178569 | 0.0151974 |
| 2023-01-01 |  | 0.0075431 | 0.0980603 | 0.00377155 |
| 2023-02-01 |  |  | 0.12069 | 0.0113146 |
| 2023-03-01 |  |  | 0.150862 | 0.00377155 |
| 2023-04-01 |  | 0.00375095 | 0.187547 | 0.0262566 |
| 2023-05-01 |  |  | 0.112528 | 0.0150038 |
| 2023-06-01 |  |  | 0.075019 | 0.00375095 |
| 2023-07-01 |  | 0.00372701 | 0.167716 | 0.011181 |
| 2023-08-01 |  | 0.00372701 | 0.163989 | 0.0298161 |
| 2023-09-01 |  | 0.00372701 | 0.1379 |  |
| 2023-10-01 |  | 0.00370966 | 0.129838 | 0.022258 |
| 2023-11-01 |  | 0.00370966 | 0.159515 | 0.0148386 |
| 2023-12-01 | 0.00370966 | 0.00370966 | 0.148386 | 0.00741932 |
| 2024-01-01 | 0.0036882 | 0.00737639 | 0.106958 |  |
| 2024-02-01 | 0.0036882 | 0.00737639 | 0.0958931 | 0.00737639 |
| 2024-03-01 | 0.0036882 | 0.0036882 | 0.136463 | 0.00737639 |
| 2024-04-01 |  | 0.0147092 | 0.110319 | 0.00367731 |
| 2024-05-01 |  |  | 0.158124 | 0.00735462 |
| 2024-06-01 |  |  | 0.0919328 |  |
| 2024-07-01 |  | 0.00366253 | 0.128189 | 0.0146501 |
| 2024-08-01 |  | 0.0109876 | 0.0988884 | 0.00366253 |
| 2024-09-01 |  | 0.0109876 | 0.150164 | 0.00366253 |
| 2024-10-01 |  | 0.00730123 | 0.138723 | 0.0109518 |
| 2024-11-01 |  | 0.00730123 | 0.146025 | 0.00365062 |
| 2024-12-01 |  | 0.00365062 | 0.167928 | 0.00365062 |
| 2025-01-01 |  |  | 0.0835244 | 0.00363149 |
| 2025-02-01 |  | 0.00363149 | 0.163417 | 0.00726299 |
| 2025-03-01 |  | 0.00363149 | 0.192469 | 0.00363149 |
| 2025-04-01 |  | 0.00363149 | 0.159786 | 0.0108945 |
| 2025-05-01 |  | 0.00726299 | 0.24331 | 0.00726299 |
| 2025-06-01 |  |  | 0.156154 | 0.0108945 |
| 2025-07-01 |  | 0.0108945 | 0.163417 | 0.00726299 |
| 2025-08-01 |  | 0.00726299 | 0.185206 | 0.00363149 |

Piroxicam

|  | Piroxicam capsule 10 mg 05203W | Piroxicam capsule 20 mg 05204X | Piroxicam dispersible tablet 10 mg 05201R | Piroxicam dispersible tablet 20 mg 05202T |
| --- | --- | --- | --- | --- |
| 2014-02-01 | 0.0982646 | 0.158078 |  | 0.0939922 |
| 2014-03-01 | 0.0811751 | 0.132444 |  | 0.0512685 |
| 2014-04-01 | 0.0553764 | 0.0851945 |  | 0.0979737 |
| 2014-05-01 | 0.0979737 | 0.132052 | 0.00425973 | 0.0724153 |
| 2014-06-01 | 0.0979737 | 0.178909 |  | 0.0809348 |
| 2014-07-01 | 0.0551715 | 0.118831 |  | 0.0848792 |
| 2014-08-01 | 0.0254638 | 0.165514 |  | 0.0297077 |
| 2014-09-01 | 0.0763913 | 0.131563 |  | 0.0679034 |
| 2014-10-01 | 0.0507607 | 0.173432 |  | 0.0846012 |
| 2014-11-01 | 0.0592208 | 0.156512 |  | 0.0803711 |
| 2014-12-01 | 0.0423006 | 0.173432 | 0.00423006 | 0.0592208 |
| 2015-01-01 | 0.0547469 | 0.105283 |  | 0.0631695 |
| 2015-02-01 | 0.0379017 | 0.151607 | 0.0084226 | 0.0673808 |
| 2015-03-01 | 0.0926486 | 0.189509 |  | 0.084226 |
| 2015-04-01 | 0.0545852 | 0.134363 |  | 0.0797783 |
| 2015-05-01 | 0.0503863 | 0.121767 |  | 0.0713806 |
| 2015-06-01 | 0.058784 | 0.167954 |  | 0.0671817 |
| 2015-07-01 | 0.0543836 | 0.146417 | 0.00418335 | 0.0334668 |
| 2015-08-01 | 0.0836671 | 0.133867 |  | 0.0794837 |
| 2015-09-01 | 0.0753004 | 0.138051 |  | 0.0418335 |
| 2015-10-01 | 0.0667095 | 0.166774 |  | 0.0375241 |
| 2015-11-01 | 0.0708789 | 0.19179 |  | 0.0583708 |
| 2015-12-01 | 0.0333548 | 0.133419 |  | 0.0458628 |
| 2016-01-01 | 0.0207439 | 0.1701 |  | 0.0207439 |
| 2016-02-01 | 0.0248927 | 0.112017 |  | 0.0456367 |
| 2016-03-01 | 0.0497855 | 0.107868 | 0.00414879 | 0.0497855 |
| 2016-04-01 | 0.0620068 | 0.111612 |  | 0.095077 |
| 2016-05-01 | 0.0289365 | 0.157084 |  | 0.103345 |
| 2016-06-01 | 0.0661406 | 0.148816 |  | 0.0868095 |
| 2016-07-01 | 0.0535034 | 0.131701 | 0.00411564 | 0.0617347 |
| 2016-08-01 | 0.0493877 | 0.152279 |  | 0.0823129 |
| 2016-09-01 | 0.00823129 | 0.18932 |  | 0.069966 |
| 2016-10-01 | 0.0574122 | 0.13943 | 0.00410087 | 0.09432 |
| 2016-11-01 | 0.0533113 | 0.118925 | 0.00410087 | 0.114824 |
| 2016-12-01 | 0.0164035 | 0.106623 | 0.00410087 | 0.0697148 |
| 2017-01-01 | 0.0448771 | 0.142791 | 0.00407973 | 0.0530365 |
| 2017-02-01 | 0.0367176 | 0.138711 |  | 0.0367176 |
| 2017-03-01 | 0.00407973 | 0.183588 |  | 0.0652757 |
| 2017-04-01 | 0.0203313 | 0.178916 |  | 0.0447289 |
| 2017-05-01 | 0.0203313 | 0.223645 |  | 0.0487952 |
| 2017-06-01 | 0.0406627 | 0.158584 |  | 0.0528615 |
| 2017-07-01 | 0.0162008 | 0.170109 | 0.00405021 | 0.0607532 |
| 2017-08-01 | 0.0202511 | 0.19036 |  | 0.056703 |
| 2017-09-01 | 0.0445523 | 0.19846 | 0.00405021 | 0.076954 |
| 2017-10-01 | 0.0323115 | 0.141363 |  | 0.0888565 |
| 2017-11-01 | 0.0484672 | 0.214063 | 0.00403893 | 0.121168 |
| 2017-12-01 | 0.0444283 | 0.137324 |  | 0.109051 |
| 2018-01-01 | 0.0321521 | 0.136646 |  | 0.0522471 |
| 2018-02-01 | 0.0321521 | 0.104494 |  | 0.116551 |
| 2018-03-01 | 0.0723422 | 0.148703 |  | 0.140665 |
| 2018-04-01 | 0.0160235 | 0.112165 |  | 0.0640942 |
| 2018-05-01 | 0.0200294 | 0.15623 |  | 0.072106 |
| 2018-06-01 | 0.0240353 | 0.116171 | 0.00400589 | 0.100147 |
| 2018-07-01 | 0.0598387 | 0.143613 |  | 0.0917526 |
| 2018-08-01 | 0.0199462 | 0.167548 |  | 0.0877634 |
| 2018-09-01 | 0.0279247 | 0.119677 |  | 0.0638279 |
| 2018-10-01 | 0.0278373 | 0.167024 |  | 0.0874886 |
| 2018-11-01 | 0.0238605 | 0.14714 |  | 0.123279 |
| 2018-12-01 | 0.0437443 | 0.119303 |  | 0.0874886 |
| 2019-01-01 | 0.0118742 | 0.106867 | 0.00395806 | 0.0672869 |
| 2019-02-01 | 0.0118742 | 0.114784 |  | 0.0633289 |
| 2019-03-01 | 0.00791611 | 0.0910353 |  | 0.0791611 |
| 2019-04-01 | 0.00394714 | 0.0710484 | 0.00394714 | 0.0710484 |
| 2019-05-01 | 0.00394714 | 0.13815 |  | 0.0749956 |
| 2019-06-01 | 0.0157885 | 0.106573 | 0.00789427 | 0.059207 |
| 2019-07-01 | 0.0196556 | 0.125796 |  | 0.121865 |
| 2019-08-01 | 0.0275178 | 0.161176 |  | 0.0550356 |
| 2019-09-01 | 0.0117933 | 0.172969 | 0.00393112 | 0.0393112 |
| 2019-10-01 | 0.027429 | 0.121471 |  | 0.0391842 |
| 2019-11-01 | 0.0352658 | 0.192003 |  | 0.0548579 |
| 2019-12-01 | 0.027429 | 0.1489 | 0.00391842 | 0.0391842 |
| 2020-01-01 | 0.015608 | 0.124864 | 0.00390199 | 0.0429219 |
| 2020-02-01 | 0.015608 | 0.120962 |  | 0.0468239 |
| 2020-03-01 | 0.00780399 | 0.128766 |  | 0.0429219 |
| 2020-04-01 | 0.0077975 | 0.0584812 |  | 0.0077975 |
| 2020-05-01 | 0.00389875 | 0.120861 |  | 0.0662787 |
| 2020-06-01 | 0.015595 | 0.140355 |  | 0.0818737 |
| 2020-07-01 | 0.0156047 | 0.191157 |  | 0.0936281 |
| 2020-08-01 | 0.0156047 | 0.195058 |  | 0.0741222 |
| 2020-09-01 | 0.0390117 | 0.140442 | 0.00390117 | 0.046814 |
| 2020-10-01 | 0.00780314 | 0.152161 | 0.00390157 | 0.0507204 |
| 2020-11-01 | 0.0195079 | 0.191177 |  | 0.0741299 |
| 2020-12-01 | 0.0234094 | 0.140457 |  | 0.0312126 |
| 2021-01-01 | 0.0194909 | 0.12864 |  | 0.0506763 |
| 2021-02-01 | 0.0311854 | 0.116945 |  | 0.0545745 |
| 2021-03-01 | 0.0233891 | 0.109149 |  | 0.0584727 |
| 2021-04-01 | 0.0194663 | 0.147944 |  | 0.0583989 |
| 2021-05-01 | 0.0194663 | 0.15573 |  | 0.0778652 |
| 2021-06-01 | 0.0545056 | 0.136264 |  | 0.0739719 |
| 2021-07-01 | 0.023342 | 0.175065 |  | 0.0700259 |
| 2021-08-01 | 0.0155613 | 0.178955 |  | 0.0894775 |
| 2021-09-01 | 0.035013 | 0.11671 |  | 0.0427936 |
| 2021-10-01 | 0.042679 | 0.170716 | 0.00387991 | 0.0775982 |
| 2021-11-01 | 0.0232795 | 0.166836 | 0.00387991 | 0.120277 |
| 2021-12-01 | 0.0116397 | 0.112517 |  | 0.0504388 |
| 2022-01-01 | 0.0347316 | 0.0694633 | 0.00385907 | 0.0231544 |
| 2022-02-01 | 0.0385907 | 0.100336 |  | 0.0848995 |
| 2022-03-01 | 0.0270135 | 0.0964768 |  | 0.119631 |
| 2022-04-01 | 0.0461206 | 0.0883979 |  | 0.0653376 |
| 2022-05-01 | 0.0307471 | 0.119145 |  | 0.119145 |
| 2022-06-01 | 0.0269037 | 0.099928 |  | 0.0768677 |
| 2022-07-01 | 0.0229276 | 0.0611403 |  | 0.0496765 |
| 2022-08-01 | 0.00764254 | 0.103174 |  | 0.0840679 |
| 2022-09-01 | 0.0152851 | 0.0802467 | 0.00382127 | 0.0726041 |
| 2022-10-01 | 0.0189968 | 0.125379 |  | 0.064589 |
| 2022-11-01 | 0.0379935 | 0.0949838 |  | 0.0417929 |
| 2022-12-01 | 0.0227961 | 0.0569903 | 0.00379935 | 0.0417929 |
| 2023-01-01 | 0.0301724 | 0.101832 |  | 0.041487 |
| 2023-02-01 | 0.0075431 | 0.0905172 | 0.0075431 | 0.0377155 |
| 2023-03-01 | 0.00377155 | 0.101832 |  | 0.0641163 |
| 2023-04-01 | 0.0112528 | 0.0600152 |  | 0.0675171 |
| 2023-05-01 |  | 0.0937737 |  | 0.075019 |
| 2023-06-01 |  | 0.0825209 |  | 0.0675171 |
| 2023-07-01 | 0.00745403 | 0.122991 |  | 0.0484512 |
| 2023-08-01 | 0.0149081 | 0.119264 |  | 0.0633592 |
| 2023-09-01 | 0.00372701 | 0.104356 |  | 0.0708133 |
| 2023-10-01 | 0.00741932 | 0.126128 |  | 0.0630642 |
| 2023-11-01 | 0.011129 | 0.114999 |  | 0.0519352 |
| 2023-12-01 |  | 0.0408062 |  | 0.0370966 |
| 2024-01-01 | 0.00737639 | 0.106958 |  | 0.0295056 |
| 2024-02-01 | 0.0036882 | 0.0811403 |  | 0.0922049 |
| 2024-03-01 | 0.0036882 | 0.0774521 |  | 0.0958931 |
| 2024-04-01 |  | 0.0882554 |  | 0.0514823 |
| 2024-05-01 | 0.00735462 | 0.0992874 |  | 0.0882554 |
| 2024-06-01 |  | 0.106642 |  | 0.0551597 |
| 2024-07-01 | 0.00732507 | 0.0659256 |  | 0.0659256 |
| 2024-08-01 | 0.0183127 | 0.102551 |  | 0.0732507 |
| 2024-09-01 | 0.00732507 | 0.120864 |  | 0.102551 |
| 2024-10-01 | 0.0219037 | 0.094916 |  | 0.0839642 |
| 2024-11-01 | 0.0146025 | 0.12047 |  | 0.0985666 |
| 2024-12-01 | 0.0255543 | 0.047458 |  | 0.0328555 |
| 2025-01-01 | 0.0108945 | 0.0980503 |  | 0.0798929 |
| 2025-02-01 | 0.029052 | 0.0653669 |  | 0.0726299 |
| 2025-03-01 | 0.00726299 | 0.105313 |  | 0.0944189 |
| 2025-04-01 | 0.014526 | 0.0689984 |  |  |
| 2025-05-01 | 0.0254205 | 0.105313 |  |  |
| 2025-06-01 | 0.00726299 | 0.0726299 |  |  |
| 2025-07-01 | 0.0326834 | 0.101682 |  |  |
| 2025-08-01 | 0.014526 | 0.108945 |  |  |

Procaine benzylpenicillin

|  | Procaine benzylpenicillin injection 1.5 g in disposable syringe 03371K |
| --- | --- |
| 2014-03-01 | 0.00854475 |
| 2014-04-01 | 0.00425973 |
| 2014-05-01 | 0.00425973 |
| 2014-10-01 | 0.00423006 |
| 2014-11-01 | 0.00846012 |
| 2014-12-01 | 0.00423006 |
| 2015-01-01 | 0.0084226 |
| 2015-08-01 | 0.00418335 |
| 2015-10-01 | 0.00416935 |
| 2015-12-01 | 0.00833869 |
| 2016-01-01 | 0.0124464 |
| 2016-03-01 | 0.00414879 |
| 2016-08-01 | 0.00411564 |
| 2016-10-01 | 0.00410087 |
| 2016-12-01 | 0.00410087 |
| 2017-03-01 | 0.00407973 |
| 2017-12-01 | 0.00403893 |
| 2018-01-01 | 0.00401901 |
| 2018-03-01 | 0.00401901 |
| 2018-06-01 | 0.00801177 |
| 2018-08-01 | 0.00398925 |
| 2018-12-01 | 0.00397675 |
| 2019-02-01 | 0.00791611 |
| 2019-05-01 | 0.00394714 |
| 2019-07-01 | 0.00786223 |
| 2019-10-01 | 0.00391842 |
| 2019-11-01 | 0.00391842 |
| 2020-04-01 | 0.00389875 |
| 2021-10-01 | 0.00775982 |
| 2021-11-01 | 0.00387991 |

Prochlorperazine

|  | Prochlorperazine injection 12.5 mg in 1 mL 05206B | Prochlorperazine suppositories 25 mg, 5 05208D | Prochlorperazine tablet 5 mg 05205Y |
| --- | --- | --- | --- |
| 2014-02-01 | 0.00427237 |  | 0.149533 |
| 2014-03-01 | 0.00427237 |  | 0.0939922 |
| 2014-04-01 |  | 0.00425973 | 0.0766751 |
| 2014-05-01 |  |  | 0.093714 |
| 2014-06-01 | 0.00425973 | 0.00425973 | 0.0766751 |
| 2014-07-01 | 0.00424396 |  | 0.0381956 |
| 2014-08-01 |  |  | 0.0636594 |
| 2014-09-01 |  |  | 0.0848792 |
| 2014-10-01 |  |  | 0.105751 |
| 2014-11-01 |  |  | 0.0592208 |
| 2014-12-01 |  |  | 0.114212 |
| 2015-01-01 |  |  | 0.0505356 |
| 2015-02-01 |  |  | 0.0589582 |
| 2015-03-01 |  |  | 0.0589582 |
| 2015-04-01 |  |  | 0.0671817 |
| 2015-05-01 | 0.00419886 |  | 0.0797783 |
| 2015-06-01 | 0.00839772 |  | 0.100773 |
| 2015-07-01 | 0.00418335 |  | 0.0627503 |
| 2015-08-01 |  |  | 0.0627503 |
| 2015-09-01 |  |  | 0.071117 |
| 2015-10-01 |  |  | 0.0958949 |
| 2015-11-01 |  |  | 0.0542015 |
| 2015-12-01 | 0.00416935 |  | 0.0958949 |
| 2016-01-01 |  |  | 0.0622318 |
| 2016-02-01 |  |  | 0.0539342 |
| 2016-03-01 | 0.00414879 |  | 0.078827 |
| 2016-04-01 | 0.00413378 |  | 0.0413378 |
| 2016-05-01 |  |  | 0.0372041 |
| 2016-06-01 |  |  | 0.0785419 |
| 2016-07-01 |  |  | 0.0535034 |
| 2016-08-01 | 0.00411564 |  | 0.069966 |
| 2016-09-01 |  |  | 0.0535034 |
| 2016-10-01 | 0.00410087 |  | 0.0451096 |
| 2016-11-01 |  |  | 0.0533113 |
| 2016-12-01 |  |  | 0.0492105 |
| 2017-01-01 |  |  | 0.0367176 |
| 2017-02-01 |  |  | 0.0326379 |
| 2017-03-01 |  |  | 0.0448771 |
| 2017-04-01 |  |  | 0.0447289 |
| 2017-05-01 |  |  | 0.0325301 |
| 2017-06-01 |  |  | 0.0650603 |
| 2017-07-01 |  |  | 0.0526527 |
| 2017-08-01 |  |  | 0.0405021 |
| 2017-09-01 |  |  | 0.0607532 |
| 2017-10-01 |  |  | 0.0525061 |
| 2017-11-01 |  |  | 0.0727008 |
| 2017-12-01 |  |  | 0.0444283 |
| 2018-01-01 |  |  | 0.0562661 |
| 2018-02-01 |  |  | 0.0241141 |
| 2018-03-01 |  |  | 0.0602851 |
| 2018-04-01 |  |  | 0.0480706 |
| 2018-05-01 |  |  | 0.0640942 |
| 2018-06-01 |  |  | 0.0480706 |
| 2018-07-01 |  |  | 0.0518602 |
| 2018-08-01 |  |  | 0.0398925 |
| 2018-09-01 |  |  | 0.0478709 |
| 2018-10-01 |  |  | 0.047721 |
| 2018-11-01 |  |  | 0.0357908 |
| 2018-12-01 |  |  | 0.0556745 |
| 2019-01-01 |  |  | 0.0752031 |
| 2019-02-01 |  |  | 0.0514547 |
| 2019-03-01 |  |  | 0.0474967 |
| 2019-04-01 |  |  | 0.0315771 |
| 2019-05-01 |  |  | 0.0671013 |
| 2019-06-01 |  |  | 0.0828899 |
| 2019-07-01 |  |  | 0.0471734 |
| 2019-08-01 |  |  | 0.0864845 |
| 2019-09-01 |  |  | 0.0707601 |
| 2019-10-01 |  |  | 0.0626948 |
| 2019-11-01 |  |  | 0.0705316 |
| 2019-12-01 |  |  | 0.0940422 |
| 2020-01-01 |  |  | 0.0702359 |
| 2020-02-01 |  |  | 0.0624319 |
| 2020-03-01 |  |  | 0.0858439 |
| 2020-04-01 |  |  | 0.0272912 |
| 2020-05-01 |  |  | 0.0545825 |
| 2020-06-01 |  |  | 0.0545825 |
| 2020-07-01 |  |  | 0.0546164 |
| 2020-08-01 |  |  | 0.0819246 |
| 2020-09-01 |  |  | 0.070221 |
| 2020-10-01 |  |  | 0.0624251 |
| 2020-11-01 |  |  | 0.0858346 |
| 2020-12-01 |  |  | 0.105342 |
| 2021-01-01 |  |  | 0.0623709 |
| 2021-02-01 |  |  | 0.0935563 |
| 2021-03-01 |  |  | 0.0623709 |
| 2021-04-01 |  |  | 0.0622922 |
| 2021-05-01 |  |  | 0.0934383 |
| 2021-06-01 |  |  | 0.0739719 |
| 2021-07-01 |  |  | 0.0816969 |
| 2021-08-01 |  |  | 0.035013 |
| 2021-09-01 |  |  | 0.0972582 |
| 2021-10-01 |  |  | 0.0698384 |
| 2021-11-01 |  |  | 0.085358 |
| 2021-12-01 |  |  | 0.0698384 |
| 2022-01-01 |  |  | 0.0501679 |
| 2022-02-01 |  |  | 0.0424498 |
| 2022-03-01 |  |  | 0.0733223 |
| 2022-04-01 |  |  | 0.0614942 |
| 2022-05-01 |  |  | 0.0807111 |
| 2022-06-01 |  |  | 0.049964 |
| 2022-07-01 |  |  | 0.0305702 |
| 2022-08-01 | 0.00382127 |  | 0.0534978 |
| 2022-09-01 |  |  | 0.0649616 |
| 2022-10-01 | 0.00379935 |  | 0.0341942 |
| 2022-11-01 |  |  | 0.0455922 |
| 2022-12-01 |  |  | 0.0683883 |
| 2023-01-01 |  |  | 0.0490301 |
| 2023-02-01 |  |  | 0.0528017 |
| 2023-03-01 |  |  | 0.0528017 |
| 2023-04-01 |  |  | 0.0337585 |
| 2023-05-01 |  |  | 0.0487623 |
| 2023-06-01 |  |  | 0.071268 |
| 2023-07-01 |  |  | 0.0260891 |
| 2023-08-01 |  |  | 0.0372701 |
| 2023-09-01 |  |  | 0.0708133 |
| 2023-10-01 |  |  | 0.0408062 |
| 2023-11-01 |  |  | 0.0482256 |
| 2023-12-01 |  |  | 0.0853221 |
| 2024-01-01 |  |  | 0.018441 |
| 2024-02-01 |  |  | 0.018441 |
| 2024-03-01 |  |  | 0.0147528 |
| 2024-04-01 |  |  | 0.0441277 |
| 2024-05-01 |  |  | 0.0294185 |
| 2024-06-01 |  |  | 0.0367731 |
| 2024-07-01 |  |  | 0.0329628 |
| 2024-08-01 |  |  | 0.0439504 |
| 2024-09-01 |  |  | 0.0329628 |
| 2024-10-01 |  |  | 0.047458 |
| 2024-11-01 |  |  | 0.0328555 |
| 2024-12-01 |  |  | 0.047458 |
| 2025-01-01 |  |  | 0.0581039 |
| 2025-02-01 |  |  | 0.0254205 |
| 2025-03-01 |  |  | 0.0435779 |
| 2025-04-01 |  |  | 0.0326834 |
| 2025-05-01 |  |  | 0.0363149 |
| 2025-06-01 |  |  | 0.00726299 |
| 2025-07-01 | 0.00363149 |  | 0.0326834 |
| 2025-08-01 |  |  | 0.029052 |

Promethazine

|  | Promethazine injection 50 mg in 2 mL 03374N |
| --- | --- |
| 2014-04-01 | 0.00425973 |
| 2014-05-01 | 0.00425973 |
| 2014-08-01 | 0.00424396 |
| 2016-02-01 | 0.00414879 |
| 2019-06-01 | 0.00394714 |
| 2022-09-01 | 0.00382127 |
| 2023-01-01 | 0.00377155 |
| 2023-07-01 | 0.00372701 |
| 2024-04-01 | 0.00367731 |
| 2024-11-01 | 0.00365062 |
| 2025-04-01 | 0.00363149 |

Roxithromycin

|  | Roxithromycin tablet 150 mg 05260W | Roxithromycin tablet 300 mg 05261X | Roxithromycin tablet for oral suspension 50 mg 05259T |
| --- | --- | --- | --- |
| 2014-02-01 | 0.303339 | 0.222163 | 0.00427237 |
| 2014-03-01 | 0.26916 | 0.388786 | 0.00427237 |
| 2014-04-01 | 0.323739 | 0.31522 | 0.00851945 |
| 2014-05-01 | 0.383375 | 0.276882 | 0.00425973 |
| 2014-06-01 | 0.391895 | 0.349298 | 0.0212986 |
| 2014-07-01 | 0.513519 | 0.377712 | 0.0169758 |
| 2014-08-01 | 0.420152 | 0.3862 | 0.0169758 |
| 2014-09-01 | 0.441372 | 0.394688 | 0.0169758 |
| 2014-10-01 | 0.355325 | 0.317254 | 0.00846012 |
| 2014-11-01 | 0.325715 | 0.181893 | 0.0126902 |
| 2014-12-01 | 0.380705 | 0.224193 | 0.0169202 |
| 2015-01-01 | 0.277946 | 0.185297 | 0.0210565 |
| 2015-02-01 | 0.29058 | 0.214776 | 0.0084226 |
| 2015-03-01 | 0.320059 | 0.299002 | 0.0126339 |
| 2015-04-01 | 0.365301 | 0.289721 | 0.029392 |
| 2015-05-01 | 0.348505 | 0.298119 | 0.0209943 |
| 2015-06-01 | 0.365301 | 0.390494 | 0.0125966 |
| 2015-07-01 | 0.334668 | 0.297018 | 0.00418335 |
| 2015-08-01 | 0.343035 | 0.288651 | 0.00836671 |
| 2015-09-01 | 0.288651 | 0.301201 | 0.0167334 |
| 2015-10-01 | 0.291854 | 0.245991 | 0.012508 |
| 2015-11-01 | 0.266838 | 0.233483 | 0.00833869 |
| 2015-12-01 | 0.296024 | 0.212637 | 0.0166774 |
| 2016-01-01 | 0.265522 | 0.161803 | 0.00414879 |
| 2016-02-01 | 0.248927 | 0.165952 | 0.00829758 |
| 2016-03-01 | 0.290415 | 0.236481 | 0.0124464 |
| 2016-04-01 | 0.343104 | 0.243893 | 0.00826757 |
| 2016-05-01 | 0.413378 | 0.281097 | 0.00826757 |
| 2016-06-01 | 0.3059 | 0.281097 | 0.00826757 |
| 2016-07-01 | 0.25517 | 0.271633 |  |
| 2016-08-01 | 0.325136 | 0.358061 | 0.0164626 |
| 2016-09-01 | 0.242823 | 0.362177 | 0.00823129 |
| 2016-10-01 | 0.241951 | 0.291162 | 0.00410087 |
| 2016-11-01 | 0.319868 | 0.205044 | 0.00820174 |
| 2016-12-01 | 0.303464 | 0.237851 | 0.00820174 |
| 2017-01-01 | 0.187668 | 0.15911 |  |
| 2017-02-01 | 0.314139 | 0.15911 | 0.00407973 |
| 2017-03-01 | 0.289661 | 0.203987 | 0.00407973 |
| 2017-04-01 | 0.199247 | 0.174849 |  |
| 2017-05-01 | 0.333434 | 0.178916 |  |
| 2017-06-01 | 0.288705 | 0.223645 |  |
| 2017-07-01 | 0.234912 | 0.283515 |  |
| 2017-08-01 | 0.291615 | 0.267314 | 0.0162008 |
| 2017-09-01 | 0.295665 | 0.283515 | 0.00810042 |
| 2017-10-01 | 0.210024 | 0.210024 | 0.00403893 |
| 2017-11-01 | 0.238297 | 0.201947 | 0.00403893 |
| 2017-12-01 | 0.262531 | 0.230219 | 0.00807786 |
| 2018-01-01 | 0.20095 | 0.176836 |  |
| 2018-02-01 | 0.192912 | 0.140665 |  |
| 2018-03-01 | 0.321521 | 0.16076 | 0.00401901 |
| 2018-04-01 | 0.300442 | 0.15623 | 0.00801177 |
| 2018-05-01 | 0.280412 | 0.192283 |  |
| 2018-06-01 | 0.280412 | 0.200294 | 0.00801177 |
| 2018-07-01 | 0.311161 | 0.191484 |  |
| 2018-08-01 | 0.343075 | 0.26329 | 0.00398925 |
| 2018-09-01 | 0.26329 | 0.235365 |  |
| 2018-10-01 | 0.246559 | 0.198838 | 0.00397675 |
| 2018-11-01 | 0.254512 | 0.214745 | 0.00397675 |
| 2018-12-01 | 0.230652 | 0.218721 | 0.00397675 |
| 2019-01-01 | 0.170196 | 0.166238 | 0.00791611 |
| 2019-02-01 | 0.205819 | 0.201861 | 0.00395806 |
| 2019-03-01 | 0.26519 | 0.174154 | 0.00395806 |
| 2019-04-01 | 0.260511 | 0.181568 |  |
| 2019-05-01 | 0.288141 | 0.189463 | 0.00394714 |
| 2019-06-01 | 0.209198 | 0.19341 | 0.00394714 |
| 2019-07-01 | 0.204418 | 0.267316 |  |
| 2019-08-01 | 0.251591 | 0.235867 | 0.00393112 |
| 2019-09-01 | 0.1769 | 0.165107 |  |
| 2019-10-01 | 0.168492 | 0.1489 |  |
| 2019-11-01 | 0.19984 | 0.117553 |  |
| 2019-12-01 | 0.235105 | 0.192003 |  |
| 2020-01-01 | 0.210708 | 0.191198 |  |
| 2020-02-01 | 0.199002 | 0.152178 | 0.00390199 |
| 2020-03-01 | 0.17559 | 0.288748 | 0.011706 |
| 2020-04-01 | 0.128659 | 0.0974687 |  |
| 2020-05-01 | 0.15595 | 0.116962 |  |
| 2020-06-01 | 0.163747 | 0.15595 |  |
| 2020-07-01 | 0.148244 | 0.171651 |  |
| 2020-08-01 | 0.19896 | 0.156047 |  |
| 2020-09-01 | 0.13264 | 0.148244 |  |
| 2020-10-01 | 0.187275 | 0.140457 |  |
| 2020-11-01 | 0.128752 | 0.0975393 |  |
| 2020-12-01 | 0.179472 | 0.128752 | 0.0117047 |
| 2021-01-01 | 0.152029 | 0.0974545 |  |
| 2021-02-01 | 0.206603 | 0.116945 |  |
| 2021-03-01 | 0.241687 | 0.132538 | 0.00389818 |
| 2021-04-01 | 0.128478 | 0.124584 |  |
| 2021-05-01 | 0.17909 | 0.186877 |  |
| 2021-06-01 | 0.249169 | 0.171303 | 0.00389326 |
| 2021-07-01 | 0.245091 | 0.140052 | 0.00389033 |
| 2021-08-01 | 0.202297 | 0.147832 |  |
| 2021-09-01 | 0.194516 | 0.108929 | 0.00389033 |
| 2021-10-01 | 0.147437 | 0.104758 |  |
| 2021-11-01 | 0.209515 | 0.116397 |  |
| 2021-12-01 | 0.197875 | 0.112517 |  |
| 2022-01-01 | 0.158222 | 0.127349 |  |
| 2022-02-01 | 0.158222 | 0.0771814 |  |
| 2022-03-01 | 0.181376 | 0.127349 |  |
| 2022-04-01 | 0.103771 | 0.099928 |  |
| 2022-05-01 | 0.165266 | 0.103771 |  |
| 2022-06-01 | 0.180639 | 0.149892 |  |
| 2022-07-01 | 0.217812 | 0.164315 |  |
| 2022-08-01 | 0.248383 | 0.164315 |  |
| 2022-09-01 | 0.233097 | 0.110817 |  |
| 2022-10-01 | 0.212764 | 0.159573 |  |
| 2022-11-01 | 0.193767 | 0.0949838 |  |
| 2022-12-01 | 0.262155 | 0.125379 |  |
| 2023-01-01 | 0.16972 | 0.075431 |  |
| 2023-02-01 | 0.14709 | 0.101832 |  |
| 2023-03-01 | 0.181034 | 0.128233 |  |
| 2023-04-01 | 0.131283 | 0.112528 |  |
| 2023-05-01 | 0.206302 | 0.142536 |  |
| 2023-06-01 | 0.202551 | 0.0975247 |  |
| 2023-07-01 | 0.152808 | 0.104356 |  |
| 2023-08-01 | 0.186351 | 0.130445 |  |
| 2023-09-01 | 0.171443 | 0.108083 |  |
| 2023-10-01 | 0.129838 | 0.100161 |  |
| 2023-11-01 | 0.100161 | 0.10758 |  |
| 2023-12-01 | 0.200322 | 0.0853221 |  |
| 2024-01-01 | 0.162281 | 0.0848285 |  |
| 2024-02-01 | 0.121711 | 0.0811403 |  |
| 2024-03-01 | 0.118022 | 0.106958 |  |
| 2024-04-01 | 0.154447 | 0.102965 |  |
| 2024-05-01 | 0.158124 | 0.121351 |  |
| 2024-06-01 | 0.158124 | 0.0845781 |  |
| 2024-07-01 | 0.150164 | 0.109876 |  |
| 2024-08-01 | 0.139176 | 0.0988884 |  |
| 2024-09-01 | 0.21609 | 0.0732507 |  |
| 2024-10-01 | 0.182531 | 0.0693617 |  |
| 2024-11-01 | 0.12047 | 0.0693617 |  |
| 2024-12-01 | 0.146025 | 0.0693617 |  |
| 2025-01-01 | 0.108945 | 0.0798929 |  |
| 2025-02-01 | 0.105313 | 0.0689984 |  |
| 2025-03-01 | 0.116208 | 0.0689984 |  |
| 2025-04-01 | 0.163417 | 0.0581039 |  |
| 2025-05-01 | 0.0762614 | 0.105313 |  |
| 2025-06-01 | 0.127102 | 0.0617354 |  |
| 2025-07-01 | 0.119839 | 0.0689984 |  |
| 2025-08-01 | 0.141628 | 0.0835244 |  |

Sodium chloride

|  | Sodium chloride I.V. infusion 154 mmol per L, 1 L 05212H |
| --- | --- |
| 2014-05-01 | 0.00851945 |
| 2014-06-01 | 0.00425973 |
| 2015-06-01 | 0.00419886 |
| 2015-07-01 | 0.00418335 |
| 2015-11-01 | 0.00416935 |
| 2015-12-01 | 0.00416935 |
| 2016-02-01 | 0.00414879 |

Temazepam

|  | Temazepam tablet 10 mg 05221T |
| --- | --- |
| 2014-02-01 | 0.495595 |
| 2014-03-01 | 0.568226 |
| 2014-04-01 | 0.660258 |
| 2014-05-01 | 0.651738 |
| 2014-06-01 | 0.562284 |
| 2014-07-01 | 0.729961 |
| 2014-08-01 | 0.577179 |
| 2014-09-01 | 0.738449 |
| 2014-10-01 | 0.579518 |
| 2014-11-01 | 0.520297 |
| 2014-12-01 | 0.642969 |
| 2015-01-01 | 0.437975 |
| 2015-02-01 | 0.450609 |
| 2015-03-01 | 0.475877 |
| 2015-04-01 | 0.503863 |
| 2015-05-01 | 0.566846 |
| 2015-06-01 | 0.743198 |
| 2015-07-01 | 0.727903 |
| 2015-08-01 | 0.585669 |
| 2015-09-01 | 0.548019 |
| 2015-10-01 | 0.554523 |
| 2015-11-01 | 0.533676 |
| 2015-12-01 | 0.646249 |
| 2016-01-01 | 0.439772 |
| 2016-02-01 | 0.522747 |
| 2016-03-01 | 0.564235 |
| 2016-04-01 | 0.475385 |
| 2016-05-01 | 0.533258 |
| 2016-06-01 | 0.661406 |
| 2016-07-01 | 0.687313 |
| 2016-08-01 | 0.596768 |
| 2016-09-01 | 0.629694 |
| 2016-10-01 | 0.529012 |
| 2016-11-01 | 0.570021 |
| 2016-12-01 | 0.619232 |
| 2017-01-01 | 0.563003 |
| 2017-02-01 | 0.579322 |
| 2017-03-01 | 0.677236 |
| 2017-04-01 | 0.569277 |
| 2017-05-01 | 0.666868 |
| 2017-06-01 | 0.683133 |
| 2017-07-01 | 0.672335 |
| 2017-08-01 | 0.615632 |
| 2017-09-01 | 0.607532 |
| 2017-10-01 | 0.674502 |
| 2017-11-01 | 0.670463 |
| 2017-12-01 | 0.650268 |
| 2018-01-01 | 0.598832 |
| 2018-02-01 | 0.554623 |
| 2018-03-01 | 0.60687 |
| 2018-04-01 | 0.616907 |
| 2018-05-01 | 0.777142 |
| 2018-06-01 | 0.697024 |
| 2018-07-01 | 0.642268 |
| 2018-08-01 | 0.698118 |
| 2018-09-01 | 0.678172 |
| 2018-10-01 | 0.660141 |
| 2018-11-01 | 0.556745 |
| 2018-12-01 | 0.556745 |
| 2019-01-01 | 0.506631 |
| 2019-02-01 | 0.625373 |
| 2019-03-01 | 0.593708 |
| 2019-04-01 | 0.540758 |
| 2019-05-01 | 0.596018 |
| 2019-06-01 | 0.596018 |
| 2019-07-01 | 0.648634 |
| 2019-08-01 | 0.609323 |
| 2019-09-01 | 0.452078 |
| 2019-10-01 | 0.548579 |
| 2019-11-01 | 0.576008 |
| 2019-12-01 | 0.509395 |
| 2020-01-01 | 0.565789 |
| 2020-02-01 | 0.479945 |
| 2020-03-01 | 0.604809 |
| 2020-04-01 | 0.272912 |
| 2020-05-01 | 0.448356 |
| 2020-06-01 | 0.647192 |
| 2020-07-01 | 0.752926 |
| 2020-08-01 | 0.686606 |
| 2020-09-01 | 0.60078 |
| 2020-10-01 | 0.577433 |
| 2020-11-01 | 0.663267 |
| 2020-12-01 | 0.600842 |
| 2021-01-01 | 0.55744 |
| 2021-02-01 | 0.522356 |
| 2021-03-01 | 0.756247 |
| 2021-04-01 | 0.564523 |
| 2021-05-01 | 0.646281 |
| 2021-06-01 | 0.673534 |
| 2021-07-01 | 0.622452 |
| 2021-08-01 | 0.618562 |
| 2021-09-01 | 0.544646 |
| 2021-10-01 | 0.616906 |
| 2021-11-01 | 0.659585 |
| 2021-12-01 | 0.508268 |
| 2022-01-01 | 0.575001 |
| 2022-02-01 | 0.54027 |
| 2022-03-01 | 0.547988 |
| 2022-04-01 | 0.622629 |
| 2022-05-01 | 0.591882 |
| 2022-06-01 | 0.687966 |
| 2022-07-01 | 0.745148 |
| 2022-08-01 | 0.726041 |
| 2022-09-01 | 0.619046 |
| 2022-10-01 | 0.76367 |
| 2022-11-01 | 0.683883 |
| 2022-12-01 | 0.577501 |
| 2023-01-01 | 0.599676 |
| 2023-02-01 | 0.690194 |
| 2023-03-01 | 0.780711 |
| 2023-04-01 | 0.63391 |
| 2023-05-01 | 0.761443 |
| 2023-06-01 | 0.768944 |
| 2023-07-01 | 0.704406 |
| 2023-08-01 | 0.82367 |
| 2023-09-01 | 0.7864 |
| 2023-10-01 | 0.76419 |
| 2023-11-01 | 0.675158 |
| 2023-12-01 | 0.600965 |
| 2024-01-01 | 0.645435 |
| 2024-02-01 | 0.726575 |
| 2024-03-01 | 0.693381 |
| 2024-04-01 | 0.720753 |
| 2024-05-01 | 0.783267 |
| 2024-06-01 | 0.768558 |
| 2024-07-01 | 0.776457 |
| 2024-08-01 | 0.853371 |
| 2024-09-01 | 0.640944 |
| 2024-10-01 | 0.865196 |
| 2024-11-01 | 0.821389 |
| 2024-12-01 | 0.631557 |
| 2025-01-01 | 0.624617 |
| 2025-02-01 | 0.653669 |
| 2025-03-01 | 0.733562 |
| 2025-04-01 | 0.642774 |
| 2025-05-01 | 0.820718 |
| 2025-06-01 | 0.708141 |
| 2025-07-01 | 0.809823 |
| 2025-08-01 | 0.835244 |

|  | Ticarcillin Powder for injection 3 g-100 mg (solvent required) 05230G |
| --- | --- |
| 2014-06-01 | 0.00427237 |
| 2014-07-01 | 0.00427237 |

Tramadol

|  | Tramadol capsule 50 mg 05232J | Tramadol capsule 50 mg 12024W | Tramadol injection 100 mg in 2 mL 05231H | Tramadol oral drops 100 mg per mL, 10 mL 05150C |
| --- | --- | --- | --- | --- |
| 2014-02-01 | 0.52123 |  |  | 0.00427237 |
| 2014-03-01 | 0.602405 |  |  |  |
| 2014-04-01 | 0.523946 |  |  |  |
| 2014-05-01 | 0.62192 |  |  |  |
| 2014-06-01 | 0.575063 |  |  |  |
| 2014-07-01 | 0.58991 |  |  |  |
| 2014-08-01 | 0.509275 |  |  |  |
| 2014-09-01 | 0.61113 |  |  |  |
| 2014-10-01 | 0.68527 |  |  |  |
| 2014-11-01 | 0.604898 |  |  |  |
| 2014-12-01 | 0.587978 |  |  |  |
| 2015-01-01 | 0.505356 |  |  |  |
| 2015-02-01 | 0.547469 |  |  |  |
| 2015-03-01 | 0.64854 |  |  |  |
| 2015-04-01 | 0.558448 |  |  |  |
| 2015-05-01 | 0.545852 |  |  |  |
| 2015-06-01 | 0.621431 |  |  |  |
| 2015-07-01 | 0.644236 |  |  |  |
| 2015-08-01 | 0.694437 |  |  |  |
| 2015-09-01 | 0.63587 |  | 0.00418335 |  |
| 2015-10-01 | 0.558692 |  |  |  |
| 2015-11-01 | 0.596216 |  |  |  |
| 2015-12-01 | 0.546184 |  |  |  |
| 2016-01-01 | 0.481259 |  |  |  |
| 2016-02-01 | 0.667955 |  |  | 0.00414879 |
| 2016-03-01 | 0.601574 |  |  |  |
| 2016-04-01 | 0.582864 |  |  |  |
| 2016-05-01 | 0.669673 |  |  |  |
| 2016-06-01 | 0.624201 |  |  |  |
| 2016-07-01 | 0.662619 |  |  |  |
| 2016-08-01 | 0.769626 |  |  | 0.00411564 |
| 2016-09-01 | 0.662619 |  |  | 0.00411564 |
| 2016-10-01 | 0.533113 |  |  |  |
| 2016-11-01 | 0.606929 |  |  |  |
| 2016-12-01 | 0.697148 |  |  |  |
| 2017-01-01 | 0.644598 |  |  |  |
| 2017-02-01 | 0.620119 |  |  |  |
| 2017-03-01 | 0.726192 |  |  |  |
| 2017-04-01 | 0.768524 |  | 0.00406627 |  |
| 2017-05-01 | 0.715663 |  |  |  |
| 2017-06-01 | 0.691265 |  |  |  |
| 2017-07-01 | 0.830293 |  |  |  |
| 2017-08-01 | 0.866745 |  |  | 0.00405021 |
| 2017-09-01 | 0.724988 |  |  |  |
| 2017-10-01 | 0.658346 |  |  | 0.00403893 |
| 2017-11-01 | 0.872409 |  |  |  |
| 2017-12-01 | 0.686619 |  |  | 0.00403893 |
| 2018-01-01 | 0.723422 |  |  | 0.00401901 |
| 2018-02-01 | 0.675194 |  |  |  |
| 2018-03-01 | 0.843992 |  |  |  |
| 2018-04-01 | 0.70103 |  |  | 0.00400589 |
| 2018-05-01 | 0.849248 |  |  |  |
| 2018-06-01 | 0.817201 |  |  |  |
| 2018-07-01 | 0.95343 |  |  | 0.00398925 |
| 2018-08-01 | 1.04119 |  |  | 0.00398925 |
| 2018-09-01 | 0.718064 |  |  |  |
| 2018-10-01 | 1.02203 |  |  | 0.00397675 |
| 2018-11-01 | 0.851025 |  |  |  |
| 2018-12-01 | 0.735699 |  |  |  |
| 2019-01-01 | 0.755989 |  |  |  |
| 2019-02-01 | 0.771821 |  |  | 0.00395806 |
| 2019-03-01 | 0.926185 |  |  | 0.00395806 |
| 2019-04-01 | 0.75785 |  |  |  |
| 2019-05-01 | 0.947313 |  |  |  |
| 2019-06-01 | 0.765744 |  |  |  |
| 2019-07-01 | 0.872708 |  |  | 0.00393112 |
| 2019-08-01 | 0.91595 |  |  |  |
| 2019-09-01 | 0.860914 |  |  |  |
| 2019-10-01 | 0.897319 |  |  | 0.00391842 |
| 2019-11-01 | 0.72099 |  |  | 0.00391842 |
| 2019-12-01 | 0.81895 |  |  |  |
| 2020-01-01 | 0.780399 |  |  |  |
| 2020-02-01 | 0.862341 |  |  |  |
| 2020-03-01 | 0.776497 |  |  |  |
| 2020-04-01 | 0.783649 |  |  |  |
| 2020-05-01 | 1.03707 |  |  |  |
| 2020-06-01 | 0.924004 | 0.175444 |  | 0.00389875 |
| 2020-07-01 | 1.13524 | 0.171651 |  | 0.00390117 |
| 2020-08-01 | 1.17035 | 0.136541 |  |  |
| 2020-09-01 | 1.24057 | 0.0975292 |  |  |
| 2020-10-01 | 1.21729 | 0.152161 |  |  |
| 2020-11-01 | 1.17047 | 0.14826 |  |  |
| 2020-12-01 | 0.987098 | 0.156063 |  |  |
| 2021-01-01 | 0.842007 | 0.105251 |  |  |
| 2021-02-01 | 0.799127 | 0.144233 |  |  |
| 2021-03-01 | 1.13437 | 0.136436 |  |  |
| 2021-04-01 | 0.899343 | 0.15573 |  |  |
| 2021-05-01 | 1.11347 | 0.268635 |  |  |
| 2021-06-01 | 1.13294 | 0.253062 |  |  |
| 2021-07-01 | 1.10096 | 0.311226 |  | 0.00778066 |
| 2021-08-01 | 1.19433 | 0.136161 |  |  |
| 2021-09-01 | 1.0465 | 0.206187 |  |  |
| 2021-10-01 | 1.03982 | 0.228915 |  |  |
| 2021-11-01 | 1.00878 | 0.166836 |  |  |
| 2021-12-01 | 0.8691 | 0.120277 |  |  |
| 2022-01-01 | 0.74866 | 0.131208 |  |  |
| 2022-02-01 | 0.864432 | 0.142786 |  |  |
| 2022-03-01 | 0.876009 | 0.162081 |  |  |
| 2022-04-01 | 0.864762 | 0.188326 |  |  |
| 2022-05-01 | 0.995437 | 0.126832 |  |  |
| 2022-06-01 | 0.926256 | 0.165266 |  | 0.00768677 |
| 2022-07-01 | 0.920926 | 0.252204 |  |  |
| 2022-08-01 | 1.10817 | 0.194885 |  |  |
| 2022-09-01 | 0.985888 | 0.1796 |  |  |
| 2022-10-01 | 1.00303 | 0.197566 | 0.00379935 |  |
| 2022-11-01 | 1.07142 | 0.208964 |  |  |
| 2022-12-01 | 0.942239 | 0.167171 |  | 0.00379935 |
| 2023-01-01 | 0.946659 | 0.184806 |  |  |
| 2023-02-01 | 1.0334 | 0.192349 |  |  |
| 2023-03-01 | 1.12015 | 0.173491 |  | 0.00377155 |
| 2023-04-01 | 0.787699 | 0.172544 |  |  |
| 2023-05-01 | 1.17405 | 0.183796 |  |  |
| 2023-06-01 | 1.02401 | 0.210053 |  |  |
| 2023-07-01 | 1.0622 | 0.141627 |  | 0.00372701 |
| 2023-08-01 | 1.07338 | 0.216167 |  |  |
| 2023-09-01 | 0.890756 | 0.182624 |  |  |
| 2023-10-01 | 0.942253 | 0.122419 |  |  |
| 2023-11-01 | 0.994188 | 0.122419 |  |  |
| 2023-12-01 | 0.830963 | 0.0704835 |  |  |
| 2024-01-01 | 0.737639 | 0.114334 |  |  |
| 2024-02-01 | 0.840909 | 0.136463 |  |  |
| 2024-03-01 | 0.81878 | 0.106958 |  |  |
| 2024-04-01 | 1.03332 | 0.165479 |  |  |
| 2024-05-01 | 0.926682 | 0.19122 |  |  |
| 2024-06-01 | 0.731785 | 0.102965 |  |  |
| 2024-07-01 | 0.937609 | 0.194114 |  |  |
| 2024-08-01 | 0.959584 | 0.131851 |  |  |
| 2024-09-01 | 0.908309 | 0.124526 |  | 0.00366253 |
| 2024-10-01 | 0.967413 | 0.164278 |  |  |
| 2024-11-01 | 0.810437 | 0.17523 |  | 0.00365062 |
| 2024-12-01 | 0.82869 | 0.135073 |  |  |
| 2025-01-01 | 0.755351 | 0.108945 |  |  |
| 2025-02-01 | 0.80256 | 0.163417 |  |  |
| 2025-03-01 | 0.842507 | 0.192469 |  |  |
| 2025-04-01 | 0.842507 | 0.130734 |  |  |
| 2025-05-01 | 0.918768 | 0.185206 |  |  |
| 2025-06-01 | 0.904242 | 0.181575 |  |  |
| 2025-07-01 | 0.944189 | 0.203364 |  | 0.00363149 |
| 2025-08-01 | 0.864296 | 0.174312 |  |  |

Triamcinolone

|  | Triamcinolone injection 10 mg in 1 mL 05233K |
| --- | --- |
| 2014-02-01 | 0.0128171 |
| 2014-03-01 | 0.00427237 |
| 2014-05-01 | 0.00425973 |
| 2014-06-01 | 0.00425973 |
| 2014-07-01 | 0.00424396 |
| 2014-08-01 | 0.00424396 |
| 2014-09-01 | 0.00848792 |
| 2014-10-01 | 0.00846012 |
| 2014-11-01 | 0.00846012 |
| 2015-01-01 | 0.0042113 |
| 2015-02-01 | 0.0084226 |
| 2015-04-01 | 0.00839772 |
| 2015-07-01 | 0.00418335 |
| 2015-08-01 | 0.00418335 |
| 2015-09-01 | 0.00418335 |
| 2015-11-01 | 0.00416935 |
| 2015-12-01 | 0.00833869 |
| 2016-02-01 | 0.00414879 |
| 2016-03-01 | 0.0124464 |
| 2016-06-01 | 0.00826757 |
| 2016-07-01 | 0.00823129 |
| 2016-09-01 | 0.00823129 |
| 2016-10-01 | 0.00820174 |
| 2016-12-01 | 0.00820174 |
| 2017-01-01 | 0.00815947 |
| 2017-02-01 | 0.00815947 |
| 2017-03-01 | 0.00407973 |
| 2017-04-01 | 0.00406627 |
| 2017-05-01 | 0.00406627 |
| 2017-07-01 | 0.00405021 |
| 2017-09-01 | 0.00810042 |
| 2017-10-01 | 0.00403893 |
| 2017-11-01 | 0.00403893 |
| 2017-12-01 | 0.00403893 |
| 2018-01-01 | 0.00401901 |
| 2018-03-01 | 0.00401901 |
| 2018-04-01 | 0.00400589 |
| 2018-05-01 | 0.00400589 |
| 2018-06-01 | 0.00400589 |
| 2018-07-01 | 0.00398925 |
| 2018-08-01 | 0.00797849 |
| 2018-09-01 | 0.00398925 |
| 2018-11-01 | 0.00397675 |
| 2018-12-01 | 0.00397675 |
| 2019-02-01 | 0.00395806 |
| 2019-03-01 | 0.00791611 |
| 2019-04-01 | 0.00394714 |
| 2019-06-01 | 0.00394714 |
| 2019-07-01 | 0.00393112 |
| 2019-10-01 | 0.00391842 |
| 2020-06-01 | 0.00389875 |
| 2020-09-01 | 0.00390117 |
| 2021-03-01 | 0.00389818 |
| 2021-04-01 | 0.00389326 |
| 2021-05-01 | 0.00778652 |
| 2021-07-01 | 0.011671 |
| 2021-09-01 | 0.00389033 |
| 2021-10-01 | 0.00775982 |
| 2022-02-01 | 0.00771814 |
| 2022-06-01 | 0.00384339 |
| 2022-09-01 | 0.00764254 |
| 2022-11-01 | 0.00379935 |
| 2022-12-01 | 0.00379935 |
| 2023-01-01 | 0.00377155 |
| 2023-04-01 | 0.0075019 |
| 2023-06-01 | 0.00375095 |
| 2023-09-01 | 0.00745403 |
| 2023-10-01 | 0.00370966 |
| 2023-11-01 | 0.00741932 |
| 2023-12-01 | 0.00741932 |
| 2024-02-01 | 0.0147528 |
| 2024-04-01 | 0.00367731 |
| 2024-05-01 | 0.00367731 |
| 2024-06-01 | 0.00735462 |
| 2024-07-01 | 0.0109876 |
| 2024-09-01 | 0.00732507 |
| 2024-10-01 | 0.00365062 |
| 2024-11-01 | 0.00365062 |
| 2024-12-01 | 0.00730123 |
| 2025-03-01 | 0.0181575 |
| 2025-04-01 | 0.00726299 |
| 2025-06-01 | 0.00726299 |
| 2025-07-01 | 0.00726299 |

Trimethoprim with sulfamethoxazole

|  | Trimethoprim with sulfamethoxazole oral suspension 200 mg with 40mg per 5 mL, 100 mL 03391L | Trimethoprim with sulfamethoxazole tablet 800 mg with 160 mg 03390K |
| --- | --- | --- |
| 2014-02-01 | 0.0213619 | 0.316156 |
| 2014-03-01 | 0.00854475 | 0.311883 |
| 2014-04-01 | 0.0340778 | 0.285402 |
| 2014-05-01 | 0.0255584 | 0.293921 |
| 2014-06-01 | 0.0127792 | 0.357817 |
| 2014-07-01 | 0.0212198 | 0.348005 |
| 2014-08-01 | 0.0212198 | 0.3862 |
| 2014-09-01 | 0.0169758 | 0.326785 |
| 2014-10-01 |  | 0.359555 |
| 2014-11-01 | 0.0126902 | 0.287644 |
| 2014-12-01 | 0.0169202 | 0.401856 |
| 2015-01-01 | 0.0084226 | 0.32427 |
| 2015-02-01 | 0.0336904 | 0.320059 |
| 2015-03-01 | 0.0252678 | 0.336904 |
| 2015-04-01 | 0.0167954 | 0.398892 |
| 2015-05-01 | 0.0377897 | 0.235136 |
| 2015-06-01 | 0.00839772 | 0.314914 |
| 2015-07-01 | 0.00418335 | 0.343035 |
| 2015-08-01 | 0.00836671 | 0.263551 |
| 2015-09-01 | 0.00836671 | 0.359768 |
| 2015-10-01 | 0.012508 | 0.245991 |
| 2015-11-01 | 0.0208467 | 0.245991 |
| 2015-12-01 | 0.0416935 | 0.312701 |
| 2016-01-01 | 0.0248927 | 0.352647 |
| 2016-02-01 | 0.0207439 | 0.344349 |
| 2016-03-01 | 0.0165952 | 0.356796 |
| 2016-04-01 | 0.0248027 | 0.343104 |
| 2016-05-01 | 0.0124014 | 0.285231 |
| 2016-06-01 | 0.0289365 | 0.264562 |
| 2016-07-01 | 0.0205782 | 0.222245 |
| 2016-08-01 | 0.0205782 | 0.296326 |
| 2016-09-01 | 0.0493877 | 0.279864 |
| 2016-10-01 | 0.00820174 | 0.250153 |
| 2016-11-01 | 0.00820174 | 0.278859 |
| 2016-12-01 | 0.0123026 | 0.200943 |
| 2017-01-01 |  | 0.208066 |
| 2017-02-01 | 0.00407973 | 0.248864 |
| 2017-03-01 | 0.0203987 | 0.240704 |
| 2017-04-01 | 0.0162651 | 0.191114 |
| 2017-05-01 | 0.0406627 | 0.195181 |
| 2017-06-01 | 0.00813253 | 0.20738 |
| 2017-07-01 | 0.0162008 | 0.153908 |
| 2017-08-01 | 0.0162008 | 0.174159 |
| 2017-09-01 | 0.00810042 | 0.202511 |
| 2017-10-01 | 0.0161557 | 0.210024 |
| 2017-11-01 | 0.0201947 | 0.18983 |
| 2017-12-01 |  | 0.210024 |
| 2018-01-01 |  | 0.12057 |
| 2018-02-01 | 0.016076 | 0.124589 |
| 2018-03-01 | 0.016076 | 0.168798 |
| 2018-04-01 | 0.00400589 | 0.172253 |
| 2018-05-01 | 0.0200294 | 0.1362 |
| 2018-06-01 | 0.00801177 | 0.188277 |
| 2018-07-01 | 0.00797849 | 0.179516 |
| 2018-08-01 | 0.0199462 | 0.183505 |
| 2018-09-01 | 0.00797849 | 0.131645 |
| 2018-10-01 |  | 0.186907 |
| 2018-11-01 |  | 0.186907 |
| 2018-12-01 |  | 0.198838 |
| 2019-01-01 |  | 0.154364 |
| 2019-02-01 |  | 0.134574 |
| 2019-03-01 | 0.00395806 | 0.16228 |
| 2019-04-01 |  | 0.13815 |
| 2019-05-01 | 0.00394714 | 0.118414 |
| 2019-06-01 |  | 0.126308 |
| 2019-07-01 |  | 0.157245 |
| 2019-08-01 |  | 0.133658 |
| 2019-09-01 | 0.00393112 | 0.129727 |
| 2019-10-01 |  | 0.101879 |
| 2019-11-01 |  | 0.0783685 |
| 2019-12-01 |  | 0.117553 |
| 2020-01-01 |  | 0.109256 |
| 2020-02-01 |  | 0.0897459 |
| 2020-03-01 | 0.00780399 | 0.132668 |
| 2020-04-01 |  | 0.09357 |
| 2020-05-01 |  | 0.0701775 |
| 2020-06-01 |  | 0.0740762 |
| 2020-07-01 |  | 0.136541 |
| 2020-08-01 | 0.00390117 | 0.105332 |
| 2020-09-01 |  | 0.109233 |
| 2020-10-01 |  | 0.136555 |
| 2020-11-01 | 0.00390157 | 0.152161 |
| 2020-12-01 |  | 0.144358 |
| 2021-01-01 |  | 0.0818618 |
| 2021-02-01 |  | 0.0935563 |
| 2021-03-01 | 0.0116945 | 0.116945 |
| 2021-04-01 | 0.0116798 | 0.0817585 |
| 2021-05-01 | 0.00389326 | 0.147944 |
| 2021-06-01 | 0.0116798 | 0.116798 |
| 2021-07-01 | 0.00778066 | 0.132271 |
| 2021-08-01 | 0.00389033 | 0.0778066 |
| 2021-09-01 |  | 0.101149 |
| 2021-10-01 | 0.00387991 | 0.131917 |
| 2021-11-01 | 0.00387991 | 0.0931178 |
| 2021-12-01 | 0.00387991 | 0.0931178 |
| 2022-01-01 |  | 0.108054 |
| 2022-02-01 | 0.00771814 | 0.0964768 |
| 2022-03-01 |  | 0.131208 |
| 2022-04-01 |  | 0.103771 |
| 2022-05-01 |  | 0.130675 |
| 2022-06-01 | 0.00384339 | 0.0883979 |
| 2022-07-01 |  | 0.0840679 |
| 2022-08-01 |  | 0.110817 |
| 2022-09-01 |  | 0.126102 |
| 2022-10-01 |  | 0.125379 |
| 2022-11-01 | 0.0113981 | 0.110181 |
| 2022-12-01 | 0.0075987 | 0.0721877 |
| 2023-01-01 | 0.0113146 | 0.0301724 |
| 2023-02-01 | 0.0150862 | 0.0452586 |
| 2023-03-01 | 0.00377155 | 0.075431 |
| 2023-04-01 |  | 0.0787699 |
| 2023-05-01 |  | 0.138785 |
| 2023-06-01 |  | 0.131283 |
| 2023-07-01 |  | 0.11181 |
| 2023-08-01 |  | 0.0969024 |
| 2023-09-01 | 0.00372701 | 0.115537 |
| 2023-10-01 |  | 0.100161 |
| 2023-11-01 |  | 0.10758 |
| 2023-12-01 |  | 0.0704835 |
| 2024-01-01 | 0.0036882 | 0.0516348 |
| 2024-02-01 |  | 0.110646 |
| 2024-03-01 |  | 0.121711 |
| 2024-04-01 |  | 0.113997 |
| 2024-05-01 | 0.00735462 | 0.139738 |
| 2024-06-01 |  | 0.110319 |
| 2024-07-01 |  | 0.117201 |
| 2024-08-01 | 0.00366253 | 0.0988884 |
| 2024-09-01 |  | 0.153826 |
| 2024-10-01 | 0.00365062 | 0.0730123 |
| 2024-11-01 |  | 0.0985666 |
| 2024-12-01 | 0.00365062 | 0.113169 |
| 2025-01-01 |  | 0.0907874 |
| 2025-02-01 | 0.00726299 | 0.105313 |
| 2025-03-01 | 0.00363149 | 0.0871559 |
| 2025-04-01 |  | 0.116208 |
| 2025-05-01 |  | 0.101682 |
| 2025-06-01 | 0.00363149 | 0.141628 |
| 2025-07-01 |  | 0.105313 |
| 2025-08-01 | 0.00363149 | 0.0726299 |

Vancomycin

|  | Vancomycin powder for injection 1 g (1,000,000 I.U.) 05083M |
| --- | --- |
| 2023-08-01 | 0.00372701 |
| 2023-09-01 | 0.00372701 |
| 2023-10-01 | 0.00370966 |
